# Peripheral nerve repair is associated with augmented cross-tissue inflammation following vascularized composite allotransplantation

**DOI:** 10.3389/fimmu.2023.1151824

**Published:** 2023-05-11

**Authors:** Ashti M. Shah, Ali Mubin Aral, Ruben Zamora, Nitin Gharpure, Fayten El-Dehaibi, Fatih Zor, Yalcin Kulahci, Huseyin Karagoz, Derek A. Barclay, Jinling Yin, Warren Breidenbach, Dmitry Tuder, Vijay S. Gorantla, Yoram Vodovotz

**Affiliations:** ^1^ Department of Surgery, University of Pittsburgh, Pittsburgh, PA, United States; ^2^ Department of Surgery, Wake Forest Institute for Regenerative Medicine, Wake Forest Baptist Medical Center, Winston Salem, NC, United States; ^3^ Department of Surgery, University of Arizona, Tucson, AZ, United States; ^4^ Plastic Surgery, San Antonio Military Medical Center, Fort Sam Houston, San Antonio, TX, United States; ^5^ Center for Inflammation and Regeneration Modeling, McGowan Institute for Regenerative Medicine, University of Pittsburgh, Pittsburgh, PA, United States

**Keywords:** transplantation, nerve-repair, inflammation, cross-tissue, systems biology

## Abstract

**Introduction:**

Vascularized composite allotransplantation (VCA), with nerve repair/coaptation (NR) and tacrolimus (TAC) immunosuppressive therapy, is used to repair devastating traumatic injuries but is often complicated by inflammation spanning multiple tissues. We identified the parallel upregulation of transcriptional pathways involving chemokine signaling, T-cell receptor signaling, Th17, Th1, and Th2 pathways in skin and nerve tissue in complete VCA rejection compared to baseline in 7 human hand transplants and defined increasing complexity of protein-level dynamic networks involving chemokine, Th1, and Th17 pathways as a function of rejection severity in 5 of these patients. We next hypothesized that neural mechanisms may regulate the complex spatiotemporal evolution of rejection-associated inflammation post-VCA.

**Methods:**

For mechanistic and ethical reasons, protein-level inflammatory mediators in tissues from Lewis rats (8 per group) receiving either syngeneic (Lewis) or allogeneic (Brown-Norway) orthotopic hind limb transplants in combination with TAC, with and without sciatic NR, were compared to human hand transplant samples using computational methods.

**Results:**

In cross-correlation analyses of these mediators, VCA tissues from human hand transplants (which included NR) were most similar to those from rats undergoing VCA + NR. Based on dynamic hypergraph analyses, NR following either syngeneic or allogeneic transplantation in rats was associated with greater trans-compartmental localization of early inflammatory mediators vs. no-NR, and impaired downregulation of mediators including IL-17A at later times.

**Discussion:**

Thus, NR, while considered necessary for restoring graft function, may also result in dysregulated and mis-compartmentalized inflammation post-VCA and therefore necessitate mitigation strategies. Our novel computational pipeline may also yield translational, spatiotemporal insights in other contexts.

## 1 Introduction

Vascularized composite allotransplantation (VCA) is a complex reconstruction option that offers patients the potential to regain structural and functional use of multiple damaged tissues in non-salvageable injuries in both combat and civilian settings. Though over 200 successful VCAs have been performed over the past decade, these procedures are performed only in select cases due to the subsequent need for lifelong immunosuppression to prevent transplant rejection (1). Transplant rejection is driven by immune dysregulation and long-term tissue damage, a significant complication even when immunosuppressant drugs are administered (2). Rejection in VCA is closely associated with innate and adaptive immune activation (1). When regulated properly, these integrated responses allow for timely healing following the surgical stress of VCA. However, when insufficient or self-sustaining, they can lead to long-lasting immune dysregulation (2, 3). It remains unknown how inflammatory mediators are expressed across skin and muscle as well as the systemic circulation, and how this compartmentally dysregulated inflammation might ultimately impact the process of transplant rejection.

Nerve repair/coaptation (NR), a major component of VCA, is necessary for peripheral nerve regeneration to restore function to the transplanted tissues, but the effect of nerve coaptation on the inflammatory cascade that causes transplant rejection is presently unknown (4–7). Nerve coaptation during VCA surgery initiates peripheral nervous system regeneration and ultimately facilitates motor control from recipient to graft and sensory input from graft to recipient, two functions that are crucial to functional transplant outcomes (8). Tacrolimus (FK506; TAC), the standard-of-care immunosuppressive agent used in VCA (9), can also enhance nerve regeneration (10, 11). Immuno-inflammation is regulated by multiple neural mechanisms, and this mode of regulation may play a role in the context of transplantation (12). However, the relevance and impact of NR and downstream nerve regeneration as contributors to, or as targets of, the inflammatory immune response after VCA are unknown.

The time scale on which transplant rejection occurs differentiates acute from chronic rejection. In the context of human hand transplantation as a primary example of VCA (13, 14), we define acute rejection (AR) as occurring within the first 2.5 years following transplant and chronic rejection (CR) as occurring anytime thereafter.

Here, we assessed the transcriptomic response to VCA in tissue samples from human hand transplants that had undergone chronic transplant rejection. These tissues were studied at the transcriptomic and proteomic levels to identify inflammatory pathways and genes that were significantly upregulated within specific tissues and across multiple sets of tissues, in combination with a reverse translational approach in a clinically realistic rat model and novel computational methods aimed at defining cross-compartment interactions.

## 2 Materials and methods

### 2.1 Overview of human hand transplant studies

Mixed skin and muscle samples were obtained at three medical centers between 2009 and 2011 from seven hand transplant patients following Institutional Review Board approval and informed consent from the patients or their legally appointed representatives. At the time of transplantation, recipient age ranged from 24 to 59 years and included 3 female and 4 male subjects. The causes of primary amputation requiring transplantation were either trauma or sepsis. The follow up duration across all patients ranged from 3.5 years to 13 years. Four of the seven patients experienced CR requiring amputation of the transplanted limbs between 3.5 years to 10 years after transplantation. Three patients died during follow up. All patients experienced multiple episodes of AR during follow up. Mixed skin and superficial muscle samples from 11 upper extremity transplants derived from 5 patients demonstrating Grade 2 or greater degree of AR as confirmed by Banff scale on blinded histopathological evaluation were used for proteomic evaluation. These patients and the samples utilized for transcriptomic and proteomic analyses are summarized in **Table 1**. Details on these patients and the biopsy samples are given below under the specific studies for which the samples were utilized.

**Table 1:** Summary of human hand transplant patients and the samples obtained from them for transcriptomic and proteomic analyses (Pt: Patient).

Pt	Transplant Type	Samples for Proteomics	Samples for Genomics	Grade 2+ AR (total number)	Samples (n) for Proteomics (AR)	Samples (n) for Genomics (CR)
1	Unilateral	Skin, Muscle	Skin, Muscle, Bone, Tendon, Vessel, Nerve	27	28	12
2	Bilateral			5	33	
3	Bilateral			5	61	
4	Unilateral	–	–	15	14	–
5	Bilateral			9	27	
6	Bilateral	–	Skin, Muscle, Bone, Tendon, Vessel, Nerve	8	–	12
7	Unilateral			14	–	12

“-”: Not available.

### 2.2 Transcriptomic studies of AR and CR in human transplant samples

To define unique gene signatures in CR after upper extremity transplantation, we carried out transcriptomic analysis of pooled, formalin-fixed, paraffin-embedded (FFPE) samples. All samples were derived from chronically rejecting hand allografts that were amputated between 3.5 to 10 years after transplantation. This included Patient 1 (also in the first group used for proteomic analysis of AR; see below) and Patients 6 and 7, each transplanted at a different center. A total of 2 unilateral and 1 bilateral upper extremity transplants derived from two male and one female recipient were used for the study of CR. The total number of Grade 2 + AR episodes in patients were as follows: Patient 1 (27 episodes), Patient 6 (8 episodes) and Patient 7 (14 episodes). Skin, muscle, bone, tendon, vessel, and nerve samples were obtained from each graft at the time of explanation, ensuring 2 samples for each tissue type (6 x 2 = 12 samples) spread across each limb to capture any differential immune rejection. These samples were pooled by tissue type due to the small mass/volume of sample

and to account for variability among the tissue. Normal (baseline) tissue samples were obtained for comparison from the recipient limb tissues (either proximal to suture line or opposite limb in case of unilateral recipients – Patient 1 and 7).

The NanoString^®^ nCounter^®^ (NanoString Technologies, Seattle, WA) (15) is a novel gene expression platform for FFPE tissue, which in the present study was used to assess the expression of a total of 782 genes in pooled samples (due to paucity of sample material). Gene expression analysis (GEA) was performed on skin, muscle, artery, nerve, and other graft tissue homogenates from pooled FFPE samples (due to the small amount of available archived FFPE tissue). Similar tissues were pooled and homogenized with a rotary homogenizer using either tissue extraction buffer (Biosource) for protein isolation or Ultraspec RNA reagent (RNA isolation). RNA was isolated and processed for GEA on a NanoString^®^ nCounter Sprint platform. Gene expression was evaluated at baseline and chronic rejection. The samples were classified by group (chronic rejection or baseline) and are also classified by tissue origin (skin, muscle, bone, tendon, vessel, and nerve). Differential gene expression analysis was conducted to understand overall and tissue-specific genetic changes occurring during CR.

Importing and centralizing the Nanostring^®^ Raw assay yielded a *m x n* raw count matrix of 782 genes by 12 samples. Artery and vein samples were grouped together under the “Vessel” group. This helped reduce our degrees of freedom when fitting the generalized linear model (GLM; see below). The *edgeR* package was used for differential expression analysis.

Of the 782 genes, 20 were “housekeeping” genes as designated by Nanostring^®^. The geNorm algorithm was used to rank the housekeeping genes by their average pairwise variation (M value) of one gene compared to the gene of the next lowest rank. An M value cutoff of.5 was used; 12 housekeeping genes were selected.

Removal of unwanted variance (RUV) was conducted to estimate factors of unwanted variance (i.e differences in run conditions between the 2 Nanostring^®^ panels, differences in sample type distribution across the 2 panels, and the impact of sampling error in tissue biopsies due to heterogeneity of chronic rejection. K = 2 factors of unwanted variance were computed using the selected housekeeping genes which were determined *a priori* to have stable expression values, therefore meeting the negative control assumption. The calculated factors of unwanted variance were 2 weight vectors of length 12 corresponding to sample specific weights. The raw count matrix was regressed onto the 2 weight vectors to obtain a RUV-normalized count matrix. The 759x12 raw count matrix was filtered to remove the selected housekeeping genes, positive controls, and negative controls. This yielded a 759x12 filtered count matrix.

### 2.3 Bioinformatics analysis of human transcriptomic data

We first carried out an analysis testing differentially expressed genes in CR samples vs. baseline samples, broken down by specific tissues (**Supplementary Figures 1A-F**). The filtered count matrix was upper quartile-normalized to minimize technical variability. The common negative binomial dispersion parameter was estimated (**Supplementary Figures 1G-H**, red line). This is the mean dispersion across all genes; the square-root of dispersion corresponds to the biological coefficient of variation (BCV). The BCV is the coefficient of variation with which the (unknown) true abundance of the gene varies between samples. Removal of Unwanted Variance is a well-accepted procedure for normalizing RNA sequencing data and removing effects of batch size and library size and uses the following formula:., where W_1_/W_2_ are the RUV weights. This formula is a quasi-likelihood negative binomial GLM using a log link function. Y(E) corresponds to the expected counts of genes, and B_0_ is the first regression coefficient. Status corresponds to whether a sample was gathered at chronic rejection or baseline. W_1_ and W_2_ are weight vectors corresponding to factors of unwanted variation (e.g., heterogeneity between Nanostring^®^ runs, differences in tissue composition within each sample). These weight vectors are calculated by regressing upper quartile normalized housekeeping genes against the known covariate of interest (status) and the factors of unknown variation *via* log-linked GLM. Since the housekeeping genes should have similar expression in samples with the same tissue status, the coefficients (nx2 matrix) corresponding to unwanted variance account for the difference between ideal and actual expression variance. This nx2 matrix is transformed into the 2 weight vectors W_1_ and W_2_ which are input into our formula. The package *edgeR* was used for the GLM model, the package *RUVSeq* was used for computation of the RUV vectors, and the package *pathfindR* was used for pathway identification/scoring.

Next, a model of the form: $Y\left(E\right)=B_{0}\times tissue\times status$ was created, where Y(E) is the upper quartile normalized filtered count matrix. This formula is a quasi-likelihood negative binomial GLM using a log-link function. Y(E) corresponds to the expected counts of genes, and B_0_ is the first regression coefficient. Status corresponds to whether a sample was gathered at chronic rejection or baseline, and tissue corresponds to the tissue type of the sample. This formula was fit to a gene-wise negative binomial generalized linear model using the common dispersion calculated previously. A likelihood ratio test was conducted to reflect the comparison: Chronic Rejection vs. Baseline. The p-values and Log2 Fold Change (log_2_ of CR count/Baseline count) were extracted. The p values were adjusted using the Benjamini-Hochberg method, and a cutoff of 0.05 was implemented.

Samples were coded as baseline (BL) or chronic rejection (CR). The RUV weight vectors calculated in the preprocessing step was used for this analysis.

### 2.4 Proteomic (Luminex™) studies of AR in human transplant samples

Samples utilized for proteomic (Luminex™) analyses are summarized in **Table 1**. The total number of samples used for protein-level expression analysis in moderate-to-severe AR were as follows: Patient 1 (n=28, 27 episodes of Grade 2 + AR), Patient 2 (n=33, 5 episodes of Grade 2 + AR), Patient 3 (n=61 samples, 5 episodes of Grade 2 + AR), Patient 4 (14 samples 15 episodes of Grade 2 + AR) and Patient 5 (27, 9 episodes of Grade 2 + AR).

### 2.5 Rat hind limb transplantation and nerve repair model

The study was approved by the Institutional Animal care and Use Committee (IACUC) of the University of Pittsburgh and by the Department of Defense Animal Care and Utilization Review Office (ACURO). Full MHC mismatched, male Lewis (LEW) and Brown-Norway (BN) rats (Charles River Laboratories, OH), between the ages of 10-12 weeks and 300-325 grams, were used in this study. Isoflurane anesthesia was administered prior to surgery. An intraperitoneal injection of Sodium Pentobarbital (Nembutal) at 50mg/kg was given to the rats prior to surgical manipulation. Isoflurane inhalation (2-3%) was administered during the surgical procedures.

A circumferential incision at the level of the inguinal limit in a LEW or BN rat was made to expose the femoral artery and vein. The limbs in the LEW and BN rats were amputated at the level of middle femur. Bone fixation was performed with an 18-gauge needle and muscle groups were repaired with 4-0 Vicryl (Ethicon Inc., Sommerville, NJ). Femoral artery and vein anastomosis were performed with 10-0 Nylon stiches (Aros, Somerville, NJ). A longitudinal incision along the femoral axis was made, the sciatic nerve was visualized, and the sciatic nerve was repaired in one group with 10-0 Nylon epineural sutures under a microscope (Aros, Somerville, NJ) but left unrepaired in the other group. The skin was closed with 4-0 Nylon suture (Aros Surgical Inc, CA) (16).

The following experimental groups were utilized (**Table 2**):

**Table 2:** Experimental groups indicating permutations of transplant type (syngeneic and allogeneic), TAC administration, and peripheral nerve repair.

Group 1 (Syngeneic -Control):	LEW recipients received MHC-matched LEW limbs (D0-D31) (n=8)
Group 2 (Syngeneic with Nerve Repair, Syngeneic + NR):	LEW recipients received MHC-matched LEW limbs with Nerve Repair (D0-D31) (n=8)
Group 3 (Allogeneic with TAC, VCA + TAC):	LEW recipients received full MHC- mismatched BN limbs, 1mg/kg of TAC was administered for 20 days (Group3a D0-D11 (n=8), Group 3b D20-D31 (n=8))
Group 4 (Allogeneic with TAC with Nerve Repair, VCA + TAC + NR):	LEW recipients received full MHC- mismatched BN limbs, nerve coaptation was performed, and 1mg/kg of TAC was administered for 20 days (Group4a D0-D11 (n=8), Group 4b D20-D31 (n=8))
Group 5 (Allogeneic - Control):	LEW recipients received full MHC- mismatched BN limbs, rats were euthanized on day 11.
Group 6 (Allogeneic with Nerve Repair, VCA + NR):	LEW recipients received full MHC- mismatched BN limbs, nerve coaptation was performed, and rats were euthanized on day 11.

Skin and muscle tissue samples were collected from hind limbs on days 0, 3, 5, 7, 9, 11, 20, 23, 25, 27, and 31 (5-6 samples from any given animal due to animal care regulations) and placed in RNA*later*™ solution (Sigma-Aldrich, St. Louis, MO). Peripheral blood samples were also collected at the same time.

### 2.6 Analysis of protein-level inflammatory mediators in human and rat tissues

All specimens were stored at -80°C until analysis. Total protein isolation and determination were carried out using the BCA protein assay kit from Pierce (Rockford, IL) with bovine serum albumin as standard as previously described (17). Human hand transplant tissues were analyzed using a Luminex™ MAGPIX^®^ apparatus (Luminex, Austin, TX) and human-specific Luminex™ beadsets (R&D Systems, Minneapolis, MN), which quantified the following analytes: ENA-78, Eotaxin (CCL11), Epidermal Growth Factor (EGF), Fractalkine, Granulocyte Colony-Stimulating Factor (G-CSF), Granulocyte Macrophage Colony-Stimulating factor (GM-CSF), GRO-α, GRO-β, Interferon-γ (IFN-γ), Interleukin (IL)-1α, IL-1β, IL-2, IL-4, IL-6, IL-10, IL-12p70, IL-13, IL-17A, IL-18, IFN-γ-inducible Protein 10 (IP-10CXCL10), Leptin, Monocyte Chemoattractant Protein (MCP-1/CCL2), Macrophage Inflammatory Protein-1α (MIP-1α/CCL3), Regulated on Activation Normal T cell Expressed and Secreted (RANTES/CCL5), TNF-α, and VEGF-A. Rat inflammatory mediators were using a rat-specific Luminex™ bead-set (EMD Millipore Kit, Billerica, MA) as per manufacturer’s specifications.

The antibody bead kit comprised a similar panel to that used for the human studies, including: Eotaxin, EGF, Fractalkine, G-CSF, GM-CSF, Keratinocyte-derived Cytokine (Gro-α/KC/CXCL1), IFN-γ, IL-1α, IL-1ß, IL-2, IL-4, IL-5, IL-6, IL-10, IL-12p70, IL-13, IL-17A, IL-18, IP-10, Leptin, LIX, MCP-1, MIP-1α, MIP-2/Gro-ß, RANTES, TNF, and Vascular Endothelial Growth Factor (VEGF). The final mediator concentrations are expressed in pg/mg protein (skin and muscle) or pg/ml (peripheral blood) as indicated. Experimental data are presented as mean ± SEM.

### 2.7 Statistical and computational analyses of proteomic data

#### 2.7.1 Statistical analyses

All statistics conducted are rooted in traditional statistics. As such, the output of the following analyses and computational models is deterministic and describes the definitive state of the inflammatory mediator dataset collected. Time-dependent changes of inflammatory mediator levels were assessed for significance using One-Way Analysis of Variance (ANOVA). Comparison between experimental groups (Syngeneic-Control vs. Allogeneic TAC) was carried out by Two-Way ANOVA using Sigma Plot (Systat Software, San Jose, CA) as indicated.

#### 2.7.2 Cross-correlation analysis

Spearman rank cross-correlation analysis was carried out to measure the strength of association among tissue-specific inflammatory mediator data in skin and muscle in both rat and human tissues using MetaboAnalyst (https://www.metaboanalyst.ca). The results were clustered and the heatmap color distribution was fixed [-1,1] in order to compare the correlation patterns among the different experimental groups.

#### 2.7.3 Volcano plot analysis

For generation of Volcano plots of statistical significance vs. magnitude of change in the inflammatory mediator data (Luminex™), we employed MetaboAnalyst (https://www.metaboanalyst.ca) using a fold change threshold set at 2.0 and significance set at *p*< 0.05.

#### 2.7.4 Dynamic network analysis

Dynamic Network Analysis (DyNA) was carried out to define, in a granular fashion, the dynamic changes of inflammatory network nodes as a function of transplant rejection severity in human hand transplant samples, essentially as described previously (16, 18). Using inflammatory mediator measurements, networks were created over four consecutive transplant severity intervals (G0-G1, G1-G2, G2-G3, and G3-G4) using MATLAB^®^ software. Network edges/connections, defined as the trajectories of tissue inflammatory mediators that move in parallel were generated if the absolute value of the Pearson correlation coefficient between any two nodes (inflammatory mediators) at the same rejection severity interval was greater or equal to a threshold of 0.90. The network complexity for each time interval was calculated using the following formula: sum (N_1_ + N_2_ +… + N_n_)/(n-1), where N represents the number of connections for each mediator and n is the total number of mediators analyzed.

#### 2.7.5 Dynamic hypergraph analysis

Dynamic hypergraphs were utilized to understand the spatiotemporal movement of inflammatory mediators over the 31 days post-VCA. Dynamic hypergraphs were inspired by the open-source package for generating static hypergraphs, HyperNetX (https://github.com/pnnl/HyperNetX ), and developed in house in python (https://anaconda.com ) (19). Hypergraphs are a form of biological network analysis in which an edge can connect more than two vertices. Specifically, graph edges were set to be inflammatory mediators and vertices were set to be tissues/compartments (peripheral blood, muscle, or skin). Dynamic hypergraphs were created for 9 overlapping dynamic time intervals of three consecutive time points at which tissue samples were collected (ex. d0, d3, d5 or d3, d5, d7) (https://github.com/ashti-shah/DynamicHypergraphs_VCA.git). Within each tissue compartment, the Pearson’s correlation coefficient was calculated for each inflammatory mediator across every dynamic time interval (3 time points). If the mediator was found to be significantly increasing (r>0.95) or significantly decreasing (r<-0.95) over that time, then the mediator was marked as an edge surrounding its respective vertex. If the same inflammatory mediator was found to be increasing or decreasing in more than one tissue compartment over the same period of time, an edge was drawn around the set of tissue compartments containing the significant mediator. Here, a hypergraph is a visual depiction of the inflammatory mediators that are significantly increasing or decreasing in strength over an interval of time.

In addition to the edge weight, or Pearson’s correlation coefficient, edge distribution was used as a second quantitative metric of this network tool. Edge distribution is a tally of the number of edges that surround 1, 2, and 3 nodes (tissue compartments), respectively and its graphical distribution demonstrates the changes in cross-compartmental intensity of inflammation.

## 3 Results

### 3.1 Transcriptomic data analysis of inflammatory pathways in skin and bone from human hand allotransplantation

To define multi-tissue responses to clinical VCA, 12 mixed-tissue samples were obtained from 7 separate vascularized composite hand transplantations that underwent chronic transplant rejection. We hypothesized that distinct inflammatory pathways would be induced in different tissues involved in VCA and would be differentially associated with transplant rejection. Analysis of gene expression in grafted skin, muscle, artery, nerve, and bone tissue homogenates was conducted to understand local genetic changes that may be associated with transplant rejection.

**Figure 1:**
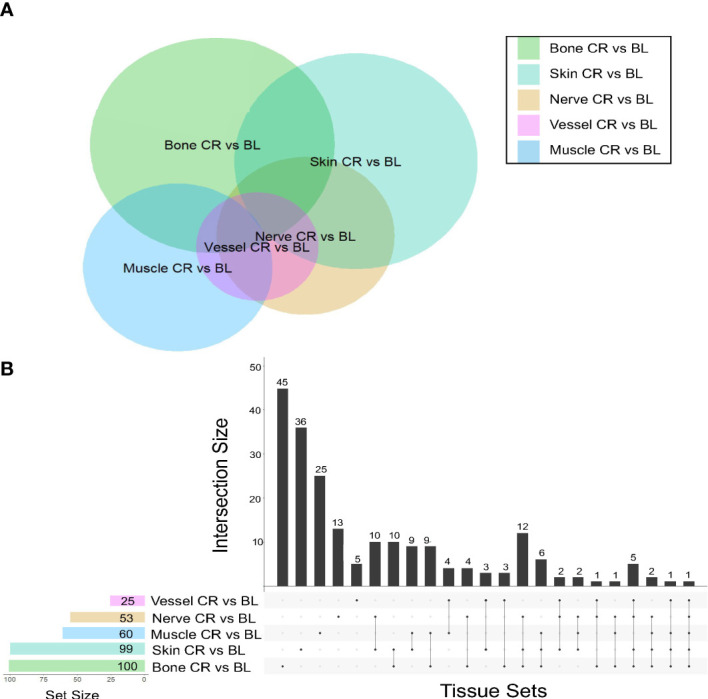
Co-expression of upregulated differentially expressed genes between tissues in human hand transplants. **(A)** Venn diagram depicting the number of differentially expressed genes between baseline and chronic rejection. The area of each circle is proportionate to the number of differentially expressed genes in the respective tissue. The area of the intersection between two or more circles is proportionate to the number of shared differentially expressed genes between two or more tissues. **(B)** Upset plot diagram depicting the number of upregulated differentially expressed genes in each tissue and sets of tissues. Each shaded dot depicts a single tissue. Lines connecting two or more dots depict a group of tissues. The height of the corresponding bar graph above each set of dots graphically illustrates the number of upregulated differentially expressed genes shared between the respective set of tissues.

This analysis demonstrated that skin samples had the greatest number of significantly differentially expressed (DE) genes between baseline and chronic rejection. Differential gene expression was conducted using a generalized linear model (GLM). The 5-way Venn diagram (**Figure 1A**) pictorially depicts the number of significantly differentially expressed (DE) genes (*p*< 0.05) that were common among different tissue types, and this is quantified in the upset plot diagram depicted in **Figure 1B**. Skin and bone demonstrated the greatest number of changes in gene expression and arteries/veins exhibited the fewest changes in gene expression (**Figure 1B**).

A subset of pro-inflammatory genes was upregulated across most sampled tissues. Significant DE genes in four or more tissues included *ccl9*, *arg-1*, *cd68*, *cxcl9*, and *ido1*. Differentially expressed genes in both skin and nerve tissue include *ccl9*, *ccl4*, and *cd36*, all of which are involved in various processes including macrophage recruitment.

A further analysis of pooled tissue was conducted to identify DE genes in CR vs. baseline across all tissues using Removal of Unwanted Variances (RUV; **Supplementary Figures 1A-F**) to remove identify pathways present in multiple tissues while correcting for batch effects and library size (**Supplementary Figures 1A-F**). This analysis pointed to genes involved in T-cell activation or T-cell-mediated cytokine secretion (e.g., *cd-247*, *cd-3d*, *cd-247*, *irf4*, and *ctss*).

**Figure 2:**
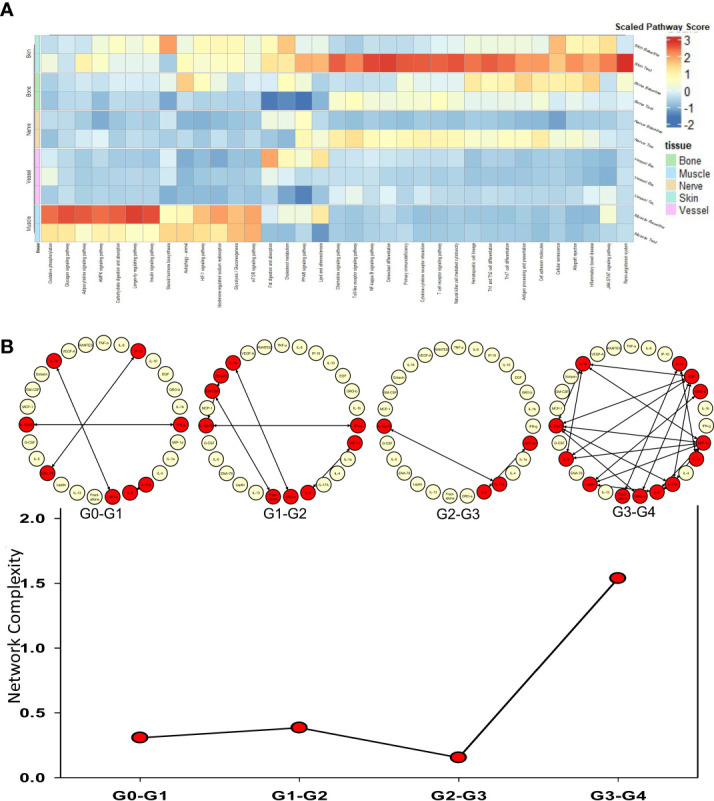
Upregulated pathways and significant inflammatory networks in tissues from human hand transplant rejection. **(A)** Heatmap showing the scaled pathway score (agglomerated z score using expression level of genes within respective pathway) depicting the most upregulated pathways in each tissue sample from human hand transplants that underwent chronic transplant rejection. **(B)** Dynamic network complexity of protein-level inflammatory mediators in human hand transplant tissue undergoing AR as a function of transplant rejection grade. Superimposed on the graph are a detailed view of the dynamic networks.

The tissues from human hand transplants that underwent CR were further analyzed to define pathways upregulated significantly at the point of transplant rejection, and pointed to upregulation of chemokine signaling, T-cell receptor signaling, Th17, Th1, and Th2 pathways in the skin in the context of CR vs. baseline (**Figure 2A**). The upregulation of these pathways was most significant in the skin, and to a lesser extent in the nerve tissue. Bone tissue samples displayed an upregulation in mTOR, antigen processing, and adipocytokine signaling pathways in CR as compared to baseline samples.

We next sought to examine the process of AR and to validate the transcriptomic findings related to CR at the protein level by carrying out multiplexed Luminex™ assays in human hand transplant samples consisting mostly of skin and muscle tissue. We also sought to define the dynamic inflammatory program accompanying VCA rejection using Dynamic Network Analysis as a function of rejection severity grade. Quantification of network complexity (20) suggested a threshold of inflammation at a rejection grade of G2-G3, after which the inflammatory response becomes more complex (**Figure 2B**). In agreement with the transcriptomic CR data (**Figure 2A**), analysis of the protein-level inflammatory mediators involved in these dynamic networks suggested the presence of an initial Th1- and Th17-cell-mediated inflammatory response (based on network connections between IFN-γ and IL-12 and IL-2 and IL-17A). This analysis further suggested the emergence of a Th2 phenotype at a rejection grade of G3-G4, as inferred by connectivity among IL-4, IL-5, and IL-10 (**Figure 2B**).

### 3.2 Reverse translation combined with cross-correlation analysis suggests that human hand transplant samples exhibit inflammatory responses similar to that of rats undergoing NR

The results of the differential gene expression analysis suggested a degree of similarity between immune-inflammatory pathways in the nerve tissue and those same pathways in the skin of rejecting human VCA tissues, namely Th1-, Th2-, and Th17-mediated inflammation (**Figure 2**; **Supplementary Figures 2**, **3**). Given the unique sets of enriched pathways depending on tissue type, we hypothesized that nerve repair (NR) might impact the dynamic process of cross-tissue inflammation. Given the ethical and practical limitations of testing this hypothesis in human VCA, we carried out a reverse-translational study of experimental VCA in rats in which Lewis rats (n= 8 per group) received either syngeneic (Lewis) or allogeneic (Brown-Norway) orthotopic hind limb transplants including TAC ± sciatic NR as described previously (12). We studied the following experimental groups: syngeneic transplant, syngeneic transplant + NR, allogeneic (i.e., VCA) +TAC, and allogeneic +TAC + NR all studied to day 31. We also examined tissues from rats undergoing VCA without TAC, which had to be euthanized at day 11 due to animal welfare considerations associated with extensive AR. We obtained skin, muscle, and plasma samples from these animals and carried out Luminex™ and network analyses.

**Figure 3:**
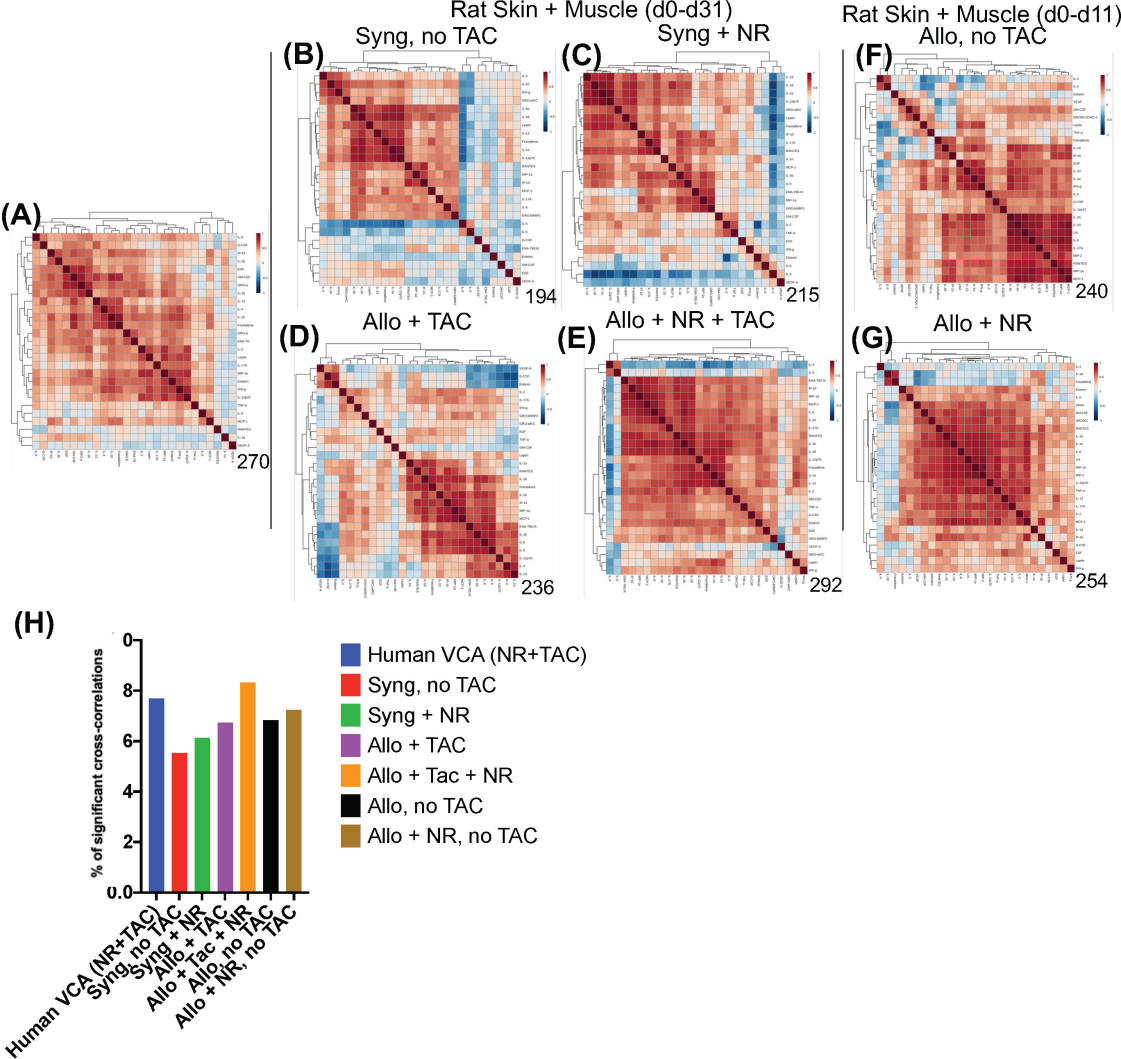
Cross-correlation analysis suggests more extensive inflammation in rats receiving NR, and VCA tissues from human hand transplants that included NR are similar to rats undergoing VCA + NR. **(A–G)** Spearman cross-correlation analyses were carried out on Luminex™ data on skin and muscle samples from patients (n = 5 patients, with 14-61 samples from each patient) who underwent hand transplantation (Panel **(A)**; rats (n=8) undergoing syngeneic transplantation and followed up to day 31 (Panel **(B)**; rats (n=8) undergoing syngeneic transplantation + NR and followed up to day 31 (n = 8; Panel **(C)** rats (n=8) undergoing allogeneic transplantation + TAC and followed up to day 31 (n=8; Panel **(D)**; rats (n=8) undergoing allogeneic transplantation + NR + TAC and followed up to day 31 (Panel **(E)**; rats (n=8) undergoing allogeneic transplantation and followed up to day 11 (given the degree of rejection observed due to the lack of TAC) (Panel **(F)**; and rats (n=8) undergoing allogeneic transplantation + NR (no TAC) and followed up to day 11 (Panel **(G)**. Panels **(A–G)**: The number of statistically significant (*p*< 0.05) cross-correlations for each clinical or experimental group is indicated on the bottom right of each panel. **(H)** The percent of statistically significant (*p*< 0.05) cross-correlations in each experimental condition calculated as such: Number of significant correlations/Maximum number of potential significant correlations.

Spearman cross-correlation analysis of time courses of protein-level inflammatory mediators from 5 human hand transplant patients (**Figure 3A**, same data as in **Figures 2B–C**) was compared to cross-correlation patterns in rat VCA (**Figures 3B–G**, same data as in **Supplementary Figure 3A** **)**. This analysis suggested a more robust inflammatory program in the presence of NR in rats undergoing either syngeneic (**Figure 3C**) or allogeneic (**Figure 3E**) transplantation as compared to syngeneic transplant controls (**Figure 3B**) or animals undergoing allogeneic transplantation + TAC (**Figure 3D**). In the (allogeneic) VCA samples, this exaggerated inflammatory program was observed despite the presence of TAC. In rats undergoing VCA in the absence of TAC (**Figures 3F–G**), a similar pattern was observed (allogeneic, no TAC, no NR having 240 significant cross-correlations vs. 254 in the allogeneic + TAC + NR group). Notably, human hand transplant samples (**Figure 3A**) exhibited 270 significant cross-correlations, closest to the number observed in rats undergoing VCA + NR (292 and 254 in **Figures 3E**, **G**, respectively). Further analysis showed that human VCA and rat allogeneic transplant with NR and TAC have the most similar ratios of significantly correlated (p<0.05) mediators (**Figure 3H**).

In support of the cross-correlation analyses, Two-Way ANOVA of inflammatory mediator time-courses showed significantly different (*p*<0.05) dynamic trajectories across the four main experimental VCA groups (**Supplementary Figure 3A**), with 26/27 significantly altered mediators in the skin, 24/27 mediators in muscle, and 22/27 mediators in peripheral blood (**Supplementary Figure 3A**). Notably, M2/Th2 cytokines associated with reduced inflammation and improved wound repair (e.g., IL-4) were significantly (*p*< 0.05) different in the allogeneic + TAC + NR group as compared to the allogeneic + TAC group (**Table 3**). This analysis was further supported by volcano plot analysis comparing the expression of inflammatory mediators between syngeneic ± NR and allogeneic + TAC ± NR, which highlighted prominent differences in the upregulation of several inflammatory mediators, particularly in the skin and muscle, following NR (**Supplementary Figure 3B**).

**Table 3:** Statistically significant changes in inflammatory mediators (syngeneic vs. syngeneic + NR and allogeneic + TAC vs. allogeneic + TAC + NR) using Two-Way Analysis of Variance (ANOVA) followed by Holm-Sidak *post hoc* test.

	SKIN		MUSCLE		PLASMA	
Mediator	syng vs. syng+NR	allo+TAC vs. allo+TAC+NR	syng vs. syng+NR	allo+TAC vs. allo+TAC+NR	syng vs. syng+NR	allo+TAC vs. allo+TAC+NR
G-CSF Eotaxin GM-CSF IL-1α Leptin MIP-1α IL-4 IL-1ß IL-2 IL-6 EGF IL-13 IL-10 IL-12p70 IFN-γ IL-5 IL-17A IL-18 MCP-1 IP-10 GRO/KC VEGF Fractalkine LIX MIP-2 TNF-α RANTES	*p*< 0.05 p< 0.05 p< 0.05 - - - - - - - - - - - - p< 0.05 - - - - - - - - - p< 0.05 p< 0.05	p< 0.05 p< 0.05 - - - - p< 0.05 - p< 0.05 - p< 0.05 p< 0.05 p< 0.05 p< 0.05 p< 0.05 p< 0.05 p< 0.05 p< 0.05 - p< 0.05 - p< 0.05 - p< 0.05 - p< 0.05 -	- - - - p< 0.05 - - - - - - - - - p< 0.05 - p< 0.05 - - - - - - p< 0.05 - - -	p< 0.05 p< 0.05 - p< 0.05 p< 0.05 p< 0.05 p< 0.05 p< 0.05 p< 0.05 - - - - p< 0.05 p< 0.05 - - p< 0.05 - - p< 0.05 p< 0.05 p< 0.05 - - - p< 0.05	- - - p< 0.05 p< 0.05 p< 0.05 p< 0.05 p< 0.05 - p< 0.05 - - p< 0.05 - p< 0.05 - p< 0.05 - p< 0.05 - - - - p< 0.05 p< 0.05 p< 0.05 -	p< 0.05 p< 0.05 - - p< 0.05 - p< 0.05 - p< 0.05 - - p< 0.05 - - p< 0.05 p< 0.05 - p< 0.05 p< 0.05 - p< 0.05 p< 0.05 p< 0.05 p< 0.05 - - -

Each individual column shows statistically significant differences (*p*< 0.05) between two experimental groups as indicated.

"-" denotes no statistical significance difference between two experimental groups, as indicated.

### 3.3 Dynamic hypergraph analysis suggests increased cross-compartment inflammation following nerve repair in rats undergoing composite tissue transplantation

While Dynamic Network Analysis and cross-correlation analysis allowed us to gain initial, broad insights into rejection-associated inflammation, we sought to define inflammatory changes that are coordinated across tissues to determine if NR impacted the cross-tissue spread of inflammation. To do so, we utilized Dynamic hypergraph (DyHyp) analysis, a graphical computational model that represents trends in inflammatory mediators across multiple tissue compartments. Here, we interpret mediators that have a statistically significant (*p*<0.05) positive correlation with themselves across a dynamic time interval (three consecutive time points) to be mediators whose response is increasing with time. In contrast, mediators that have a statistically significant negative correlation with themselves across a dynamic time interval are those whose response is decreasing with time. Graphs of edge distribution highlight trends in the localization of growing or attenuating inflammatory responses over time.

**Figure 4:**
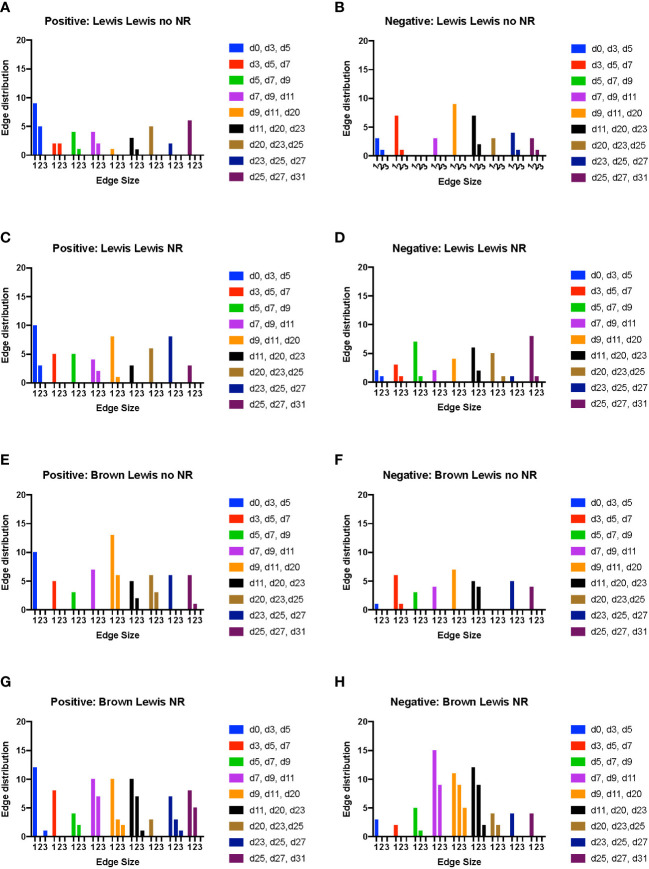
Dynamic hypergraph analysis reveals that VCA with NR results in greater trans-compartmental inflammation than VCA without NR. Dynamic hypergraphs were constructed for Luminex™ data obtained from each of the four indicated experimental groups of rats for all dynamic time intervals (n = 8 rats per group). **(A–H)** a sum of the number of edges, or inflammatory mediators, that surrounded just 1, 2, or all 3 nodes, or tissue compartments, was calculated and plotted as a bar graph for each time interval. The labels ‘positive’ and ‘negative’ refer to the upregulation and downregulation of inflammatory mediators respectively.

In rats receiving a syngeneic transplant (without TAC), NR was associated with earlier cross-compartmental inflammation and reduced downregulation of inflammation (**Figures 4A–D** **)**. Compared to rats receiving syngeneic transplant without NR, rats with NR had greater inflammation in nearly all compartments. In addition, cross-compartmental inflammation was apparent again over the time interval spanning d7-d20 in rats with NR, in contrast with d11-d23 in rats without NR (**Figures 4A, C** **)**. We interpret the cross-compartmental inflammation at much earlier time points (e.g., d0-d11) to be due to the VCA procedure itself rather than the effect of NR. Furthermore, the resolution of inflammation over the early intervals spanning d0-d7 was inferred to be severely reduced in rats undergoing syngeneic transplantation + NR *vs*. rats without NR (**Figures 4B, D** **)**. Together, a study of the edge distribution in rats receiving syngeneic transplantation ± NR suggests that NR facilitates earlier cross-compartmental inflammation and impairs early downregulation of inflammation.

Examination of tissue compartments in which inflammation either peaked or diminished over time suggested that NR facilitates greater cross-compartmental inflammation between the muscle and skin as well as between skin and plasma, but not between muscle and plasma (**Supplementary Figures 4A, C** **)**. In nearly all sets of tissue compartments across all time points, inflammation was inferred to be downregulated in a greater set of inflammatory mediators in the absence of NR compared to settings in which NR was performed (**Supplementary Figures 4B, 4D** **)**.

In rats receiving allotransplantation, NR was also associated with much greater cross compartmental inflammation compared to allotransplantation without NR. However, unlike -syngeneic transplants, allogeneic transplants with NR also exhibited much greater downregulation of inflammatory mediators compared to allogeneic transplants without NR (**Figure 4F, H** **)**. In animals undergoing allogeneic transplant + NR, a number of mediators across two later sets of dynamic time intervals (d23-d27 and d25-d31) spanned subsets of skin, muscle, and plasma, suggesting a coordinated pro-inflammatory response associated with NR (**Figures 4E, G** **).** Specifically, CXCL10/IP-10 was upregulated in plasma, skin, and muscle in parallel during the interval between d23-d27. Furthermore, IL-17A was upregulated in both plasma and muscle, and TNF α and VEGF were upregulated in both skin and plasma during the interval between d25-d31.

We next sought to define the impact of NR without the confounding role of major histocompatibility complex (MHC) mismatch. Dynamic hypergraphs for each time interval for syngeneic transplant ± NR are depicted in **Supplementary Figures 5**, **6**. Of note, Interleukin (IL)-6 was upregulated consistently during the first 11 days in the skin of animals that underwent syngeneic transplantation with NR, but only increased in the plasma on d5-d9 and the plasma and skin over d7-d11. IL-17A was a significant cross-compartment mediator at early time points in syngeneic transplant with and without NR. The eventual downregulation of IL-17A was only evident in NR samples. Thus, we conclude that nerve coaptation may be necessary for the eventual downregulation of the VCA rejection-associated (21) pro-inflammatory mediator IL-17A.

We then examined the impact of NR in the context of full MHC mismatch; dynamic hypergraphs for each time interval for allogeneic transplant ± NR are depicted in **Supplementary Figures 7**, **8**. Nerve coaptation appears to facilitate early cross-compartmental inflammation in allogeneic transplant that does not appear otherwise until days 9-11, resulting in cross-compartmental elevations in eotaxin, MIP-1α, MIP-2, EGF, VEGF, leptin, GRO/KC, LIX, and RANTES at various intervals up until the interval day 9-11. Interleukin-10 was downregulated in all tissue compartments during the interval day 9-20, but not in the absence of NR. Overall, NR was inferred to facilitate cross-compartmental downregulation of inflammation between days 7-25, but allogeneic transplant without NR only exhibited a brief downregulation of inflammation during the narrow interval between days 11-23. Thus, we infer that, at multiple time points, NR facilitates downregulation of inflammation in allogeneic transplant to a greater extent than transplant without NR.

**Figure 5:**
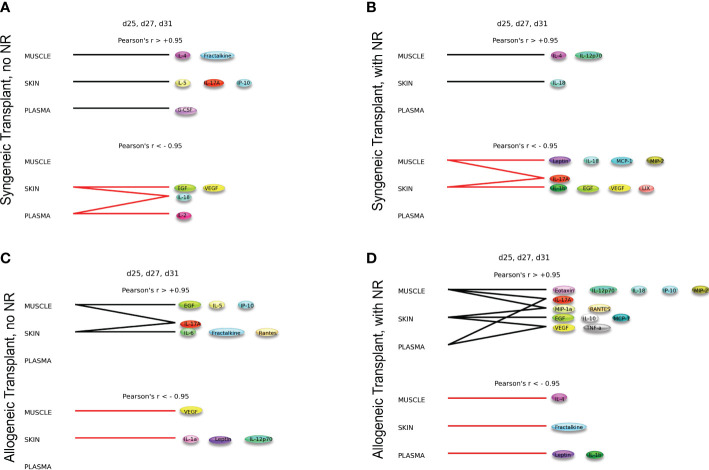
Dynamic hypergraphs depict cross-compartmental trends in inflammation. The following graphs model trends in inflammation across all four experimental conditions over the time interval d25, d27, and d31. Summary of hypergraph analysis detailed in **Supplementary Figures 6**,–**8**. **(A)** Syngeneic transplant no NR results in the downregulation of four inflammatory mediators in the skin and/or plasma. **(B)** Syngeneic transplant performed with NR results in downregulation of inflammation in the muscle and skin. **(C)** Allogeneic transplant without NR results in cross-compartmental inflammation between the muscle and skin. **(D)** Allogeneic Transplant with NR results in severe inflammation and multiple cross-compartmental trends in inflammation.

The syngeneic and allogeneic transplant hypergraph analyses are summarized in **Figure 5**. Syngeneic transplant with no NR was associated with the downregulation of four inflammatory mediators in the skin and/or plasma. In contrast, syngeneic transplant performed with NR was associated with greater downregulation of inflammatory mediators compared to the syngeneic transplant without NR. Notably, NR appeared to facilitate the downregulation of IL-17A in both muscle and skin, ultimately downregulating a greater set of pro-inflammatory mediators in both those tissues. Additionally, the circulating compartment contained no inflammatory mediators that were either increasing or decreasing with time in syngeneic transplants. Allogeneic transplant without NR was associated with lesser overall and cross-compartmental inflammation as compared to inflammation observed when NR was performed. In allogeneic transplant with NR, IL-17A was jointly elevated in both the muscle and plasma, VEGF and TNF-α were jointly elevated in the skin and plasma, and MIP-1α and RANTES were jointly elevated in the muscle and skin. 4 Discussion

In the present study, we sought to better understand the mechanisms regulating the spatiotemporal dynamics of immuno-inflammation associated with transplant rejection, expanding on our recent computational modeling work in experimental VCA (12) and trauma/hemorrhagic shock (22). Taken together, these analyses suggest that NR is both detrimental (as this procedure is associated with increased early cross-compartmental inflammation) but also advantageous, since NR helps drive resolution of inflammation at later time points. To our knowledge, this is the first holistic study involving data from both humans and rodents in which multi-tissue transplant data have been analyzed using novel transcriptomics; multiplexed assays of key cytokines, chemokines, and growth factors; and novel, spatiotemporally oriented computational modeling methods. Using this workflow, we studied up to five different tissue types, in the process implicating peripheral neural pathways in the cross-tissue modulation of Th1-, Th2-, and Th17-related pathways.

To derive these insights, we leveraged the strengths of the NanoString^®^ system. Here, we applied this technology for the first time in FFPE samples from human hand transplants that developed CR occurring due either non-responsiveness to, or non-compliance with, immunosuppression therapy. Protocol and for-cause biopsies from these recipients have identified both AR and CR that was correlated with clinical presentation of these conditions (Gorantla et al, submitted). The utilization of FFPE material allows for molecular-histological correlation on the same tissue even in archived samples dating several years. In this case, some samples were over a decade old, thus allowing for better integration with techniques requiring separately processed tissue. Our studies therefore raise the prospect of systems-level insights from archival tissue using similar transcriptomic approaches.

Both skin and nerve tissues from human hand transplant patients showed similar transcriptomic patterns of upregulated inflammatory pathways in CR tissues as compared to baseline, and this insight was foundational in driving the hypothesis that nerve coaptation might in some way impact the cross-tissue spread of immuno-inflammation. In line with prior observations by others (21), key upregulated pathways in VCA include the T-cell receptor signaling pathway, cytokine-cytokine receptor interaction, Th1/Th2 cell differentiation, and Th17 cell differentiation. Notably, these pathways were also inferred at the protein level based on analysis of dynamic networks. These pathways are related to immuno-inflammation and are associated with several of the inflammatory mediators found to be at higher concentrations in both human and rat tissues. The Th1/2 differentiation pathway is associated broadly with immuno-inflammation (23) and transplant rejection (21, 24, 25). The type 17 immune pathway, whose elevation in the skin of rats undergoing VCA likely involves IL-17A secreted by γδ T cells (26), has been implicated in transplant rejection in multiple contexts including VCA (21, 27–29). Th1, Th2, and Th17 pathways are upregulated and correlations among inflammatory mediators secreted by these three cell types suggests a complex interplay among these three inflammatory circuits. Notably, there appears to be a threshold of inflammatory complexity that differentiates a rejection severity of G2-G3 and G3-G4.

Individual differentially expressed genes of interest include *arg-1*, *cd68*, *cxcl9*, and *ido1*. Arg-1 is expressed by M2 macrophages (30) and is also responsible for the induction of immunosuppressive function by myeloid-derived suppressor cells (31). The upregulation of regulatory macrophages by Arg-1 has been correlated with increased survival of skin allografts in mice (32). Differentially expressed proinflammatory genes included CD-68, which is expressed by macrophages, cxcl9, a central inflammatory chemokine whose expression is often associated with inflammation in the dermis, and idol1, which positively regulates the immune response of mesenchymal stem cells (33–35). Increased expression of CD-68 has been correlated with decreased patient and graft survival in patients receiving kidney transplants (36). The differential expression of immune-suppressive Arg-1 contrasts with the expression of pro-inflammatory genes such as *cd69*, *cxcl1*, and *ido1*. The differential expression of both pro- and anti-inflammatory genes sheds light on the complex inflammatory cascade that ultimately leads to chronic rejection.

The initial observation of potential similarity in nerve and skin of human VCA tissues led us to hypothesize that some aspect of nerve coaptation might impact the cross-tissue, dynamic propagation of immuno-inflammation. Given that nerve coaptation is the standard of practice in VCA and therefore could not be modified in the clinical setting due to ethical considerations, we tested this hypothesis *via* a reverse translation approach using an animal model that we have utilized previously to derive novel insights into the dynamics of immuno-inflammation in VCA (12, 37).

These studies led us to conclude that NR was associated with elevations in multiple cytokines and chemokines, and computational modeling suggested that NR was associated with a higher degree of cross-compartment immuno-inflammation. This conclusion is consistent with the concept of a ‘brain-skin axis’ in which inflammatory mediators in the skin and systemic cortisol and cortisol-releasing hormones are upregulated in response to psychological stress (38, 39). We hypothesize that the skin serves as a rheostat of the extent of tissue damage in the context of VCA, through the regulation of immuno-inflammation *via* this mechanism.

These insights would not be possible without the use of novel computational modeling approaches. Systems and computational biology offer a new lens by which the spread of cytokines and chemokines post-VCA throughout muscle, skin, and plasma can be modeled, as we have shown in recent studies (12). In the hypergraph formalism, inflammatory mediators could be viewed as network edges and biological compartments could be treated as network nodes. Here, we augmented this modeling framework (22) by examining hypergraphs over time intervals (DyHyp), which highlighted the striking impact of NR on augmenting cross-tissue immuno-inflammation. For example, one quantitative metric of hypergraphs, edge distribution, demonstrated that NR facilitates earlier cross-compartmental inflammation and impaired downregulation of inflammatory mediators even in the context of syngeneic limb transplantation, and this was further augmented in the allogeneic setting.

The core insight that emerged from this modeling analysis is that cross-compartmental inflammation is facilitated by NR and that nerve coaptation connects multiple tissue compartments, thereby preventing localized decreases in inflammatory mediators. In the context of Wallerian degeneration following peripheral nerve injury, multiple inflammatory mediators are upregulated early following injury, but inflammation is attenuated at later time points (40). Furthermore, peripheral nerves can release immunomodulatory peptides in the skin, such as substance P, which leads to the increased production of IL-6 and TNFα by local macrophages (38). Additionally, T-lymphocyte proliferation can also be induced by nanomolar concentrations of substance P (41). The upregulation of pathways involving T-cell proliferation in human VCA with NR and the increased production of IL-6 and TNF−α in rat VCA with NR further supports the notion that NR, while functionally useful, perpetuates widespread inflammation beginning in the skin (42). In a literature review of healing of pre-existing inflammatory cutaneous lesions after injury to the respective innervating peripheral nerves, 19/23 reported cases experienced an improvement or complete resolution of the inflammatory cutaneous wounds (39). Thus, the peripheral nervous system may inhibit the attenuation/resolution of the immuno-inflammation.

Our studies therefore present a paradox. Peripheral nerve regeneration is essential for the motor and sensory recovery of the graft, thus ultimately driving successful transplantation, and yet our studies suggest that nerve coaptation promotes the cross-tissue propagation of potentially harmful immuno-inflammation (43). We therefore suggest the need to derive novel adjunct treatments that would minimize the pro-inflammatory impact of nerve coaptation while promoting functional innervation.

One approach might center around Tacrolimus (FK506), a widely used immunosuppressant in solid organ (44) and composite tissue transplantation (45) that was utilized in our clinical and experimental studies. The beneficial effects of Tacrolimus on nerve regeneration were demonstrated in multiple studies (46). The neuroprotective effects FK506 may be due to its pharmacologic effects as a calcineurin inhibitor (47) or *via* the 52-kD FK506-binding protein receptors (48, 49). Another possible avenue could involve IL-6, which was elevated in a manner associated with NR. IL-6 levels are increased in injured nerve as contrast to those seen in healthy nerves (50–52). Interestingly, Koulaxouzidis et al. reported in a mouse model of sciatic injury that blocking antibodies against the IL-6 receptor (IL-6Rα) at the coaptation site were beneficial with regard to peripheral nerve regeneration (53).

There are multiple limitations of our study. The first, as noted above, is that mechanistically testing in humans the hypothesis that neural circuits underlie the cross-tissue propagation of immuno-inflammation is likely unethical, which is the reason we turned to a reverse-translation approach using a rodent model of VCA. As such, the time frame of the human data is on the order of years, while for practical reasons (including the rate of growth of rats) the experimental timeline is on the order of a month. Rat hind limb transplantation is a widely used surgical model for VCA, mostly utilized in transplant immunology but also preferred for studies of nerve regeneration and function to evaluate outcomes (54–56). However, this experimental model lacks evaluation of specific functional outcomes. Functional tests were not in scope of this study, but the model allowed us to analyze the outcomes in the beginning of transplant, which is crucial to predict long term graft survival. Further studies should be planned to predict outcomes with these preliminary results.

Another limitation is that we did not directly assess cell populations and their transcriptomic or other functional programs, and thus we can only infer a role for specific immune cell populations based on bulk transcriptomics and multiplexed analysis of a subset of protein-level mediators. We notably also observed the upregulation of a unique subset of pro-inflammatory signaling pathways in bone tissue, which suggests that the bone tissues may be one of the tissues responsible for the inflammatory cascade that is characteristic of transplant rejection. The expression of the osteoclast differentiation pathway was also notably reduced in bone tissue in chronic rejection compared to baseline. This is an important aspect of VCA that could not be studied here and thus requires further investigation.

Additional work is needed to expand the conclusions of this study. The number of human hand transplant subjects could be increased. Additionally, it may be possible to obtain skin and plasma samples longitudinally before CR ensues to better model how CR develops in the skin and spreads to the plasma. In the rat studies, tissue samples from bone and nerve could be studied to further assess the role of these two tissues in mediating cross-tissue inflammation given their significant roles in the human CR models. Future work may leverage the insights from this study to define novel biomarkers of transplant rejection and/or to identify better targets for immunosuppression to prevent transplant rejection.

The results described here not only highlight a novel mechanism for transplant rejection but implicate the effect of NR on cross-compartmental inflammation. These studies may have implications for the development of better-targeted therapeutics for inhibiting transplant rejection now that a novel mechanism for CR has been elucidated and inflammatory mediators perpetuating cross tissue inflammation have been identified. Finally, these studies support the further use of spatiotemporal computational models to better understand complex biological responses.

## Data availability statement

The datasets presented in this article are not readily available because of ethical and privacy restrictions. Requests to access the datasets should be directed to the corresponding authors.

## Ethics statement

All human studies were conducted in accordance with the Institutional Review Board. The patients/participants provided their written informed consent to participate in this study. The animal study was reviewed and approved by Institutional Animal Care and Use Committee (IACUC) of the University of Pittsburgh.

## Author contributions

Conceptualization: YV, VG. Methodology: AS, AA, RZ, NG, YV, VG. Software: AS, NG. Investigation: AA, FE-D, FZ, YK, HK, DB, JY. Visualization: AS, RZ, NG. Funding acquisition: YV, VG. Project administration: YV, VG. Supervision: YV, VG. Writing – original draft: AS, AA, RZ, NG, YV, VG. Writing – review and editing: AS, AA, RZ, NG, WB, DT, YV, VG. All authors contributed to the article and approved the submitted version.
