# Supplementary figures and images for: Peripheral nerve repair is associated with augmented cross-tissue inflammation following vascularized composite allotransplantation
Source: Front Immunol. 2023 May 11;14:1151824. doi: 10.3389/fimmu.2023.1151824 (PMC10213935; doi:10.3389/fimmu.2023.1151824)

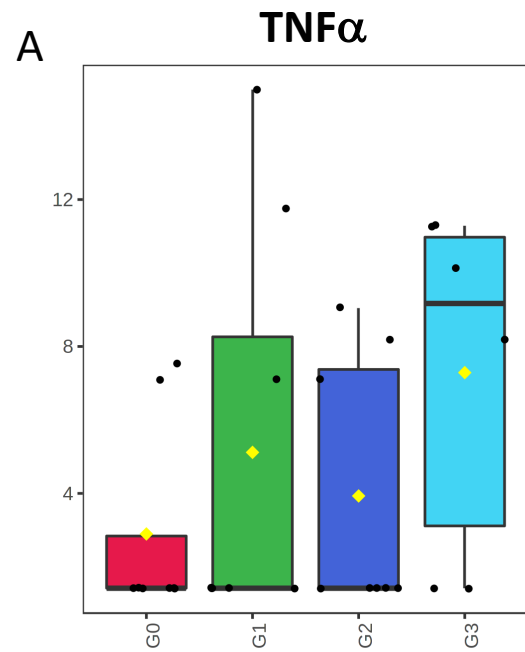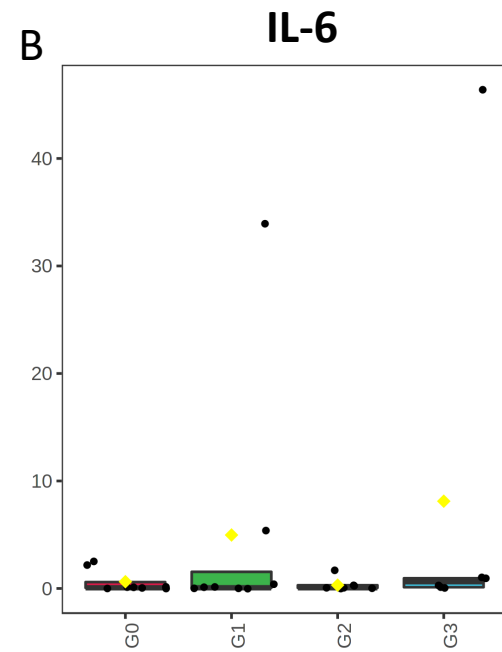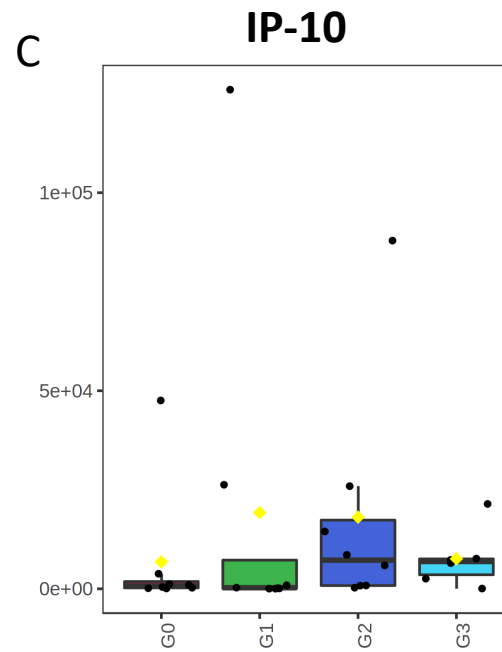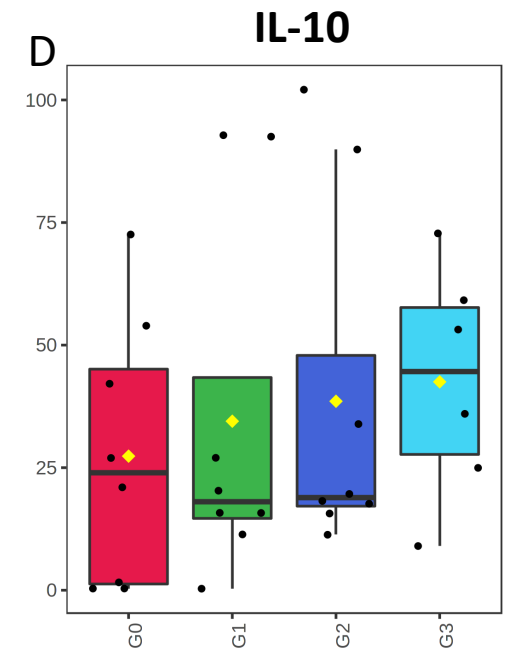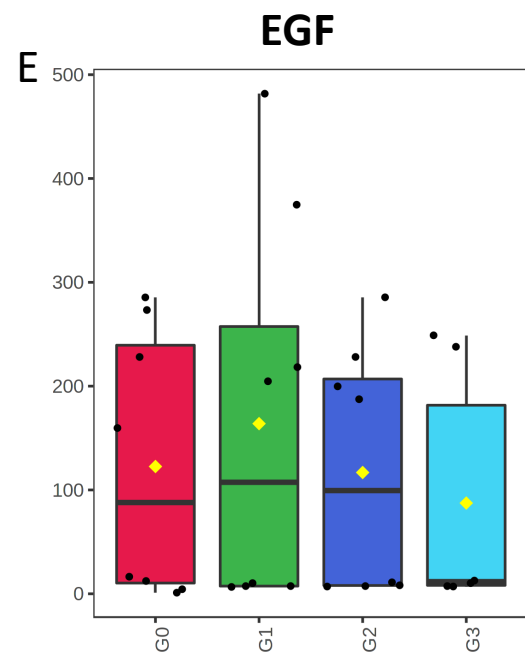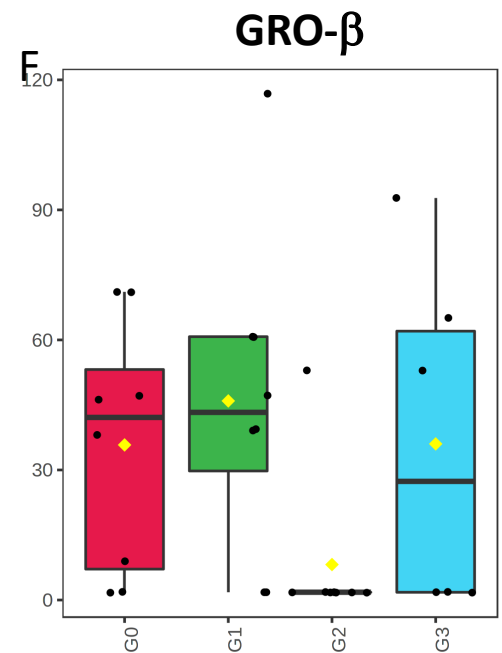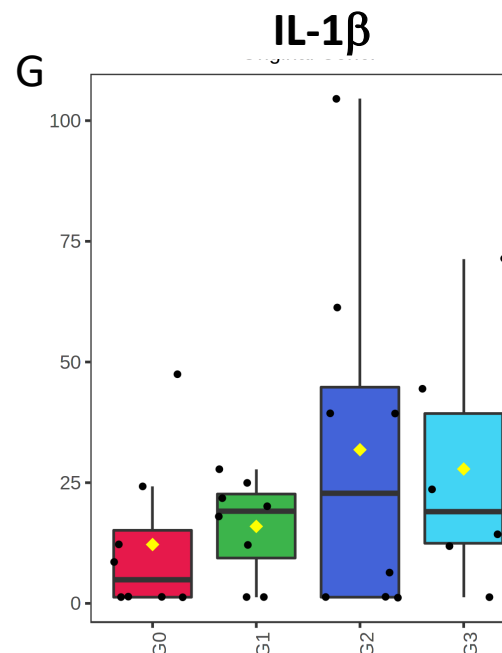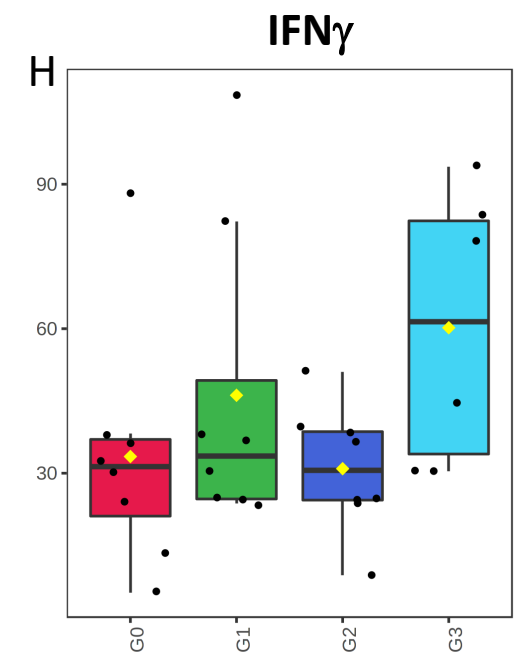

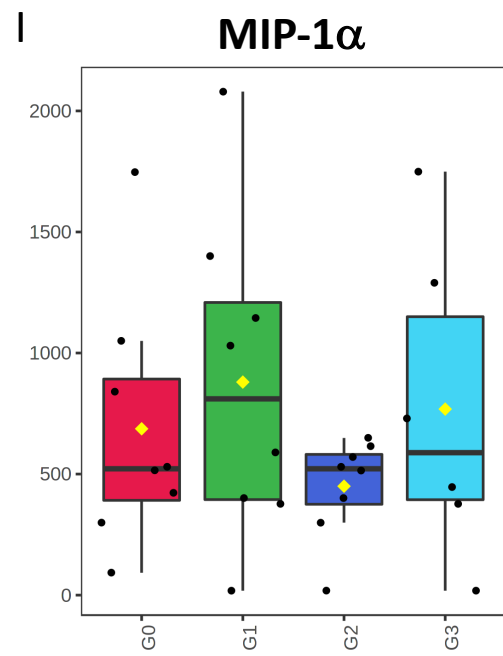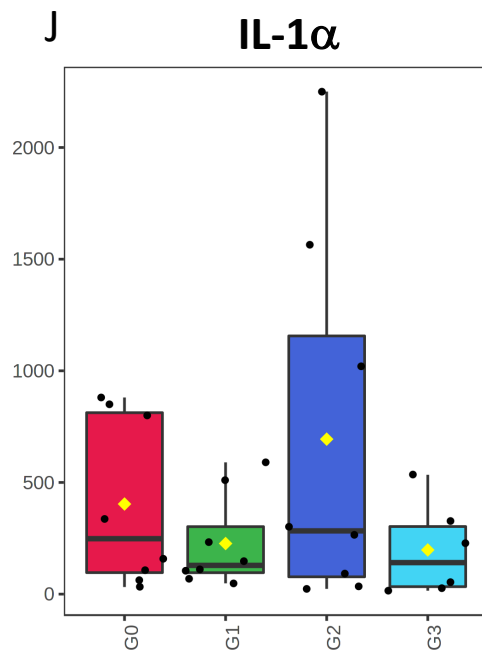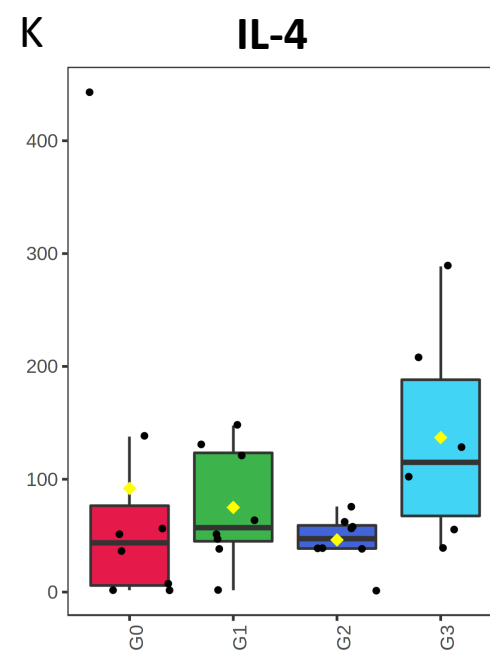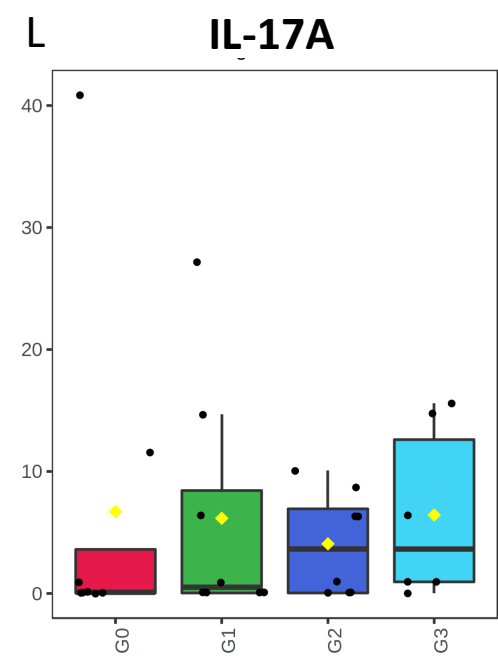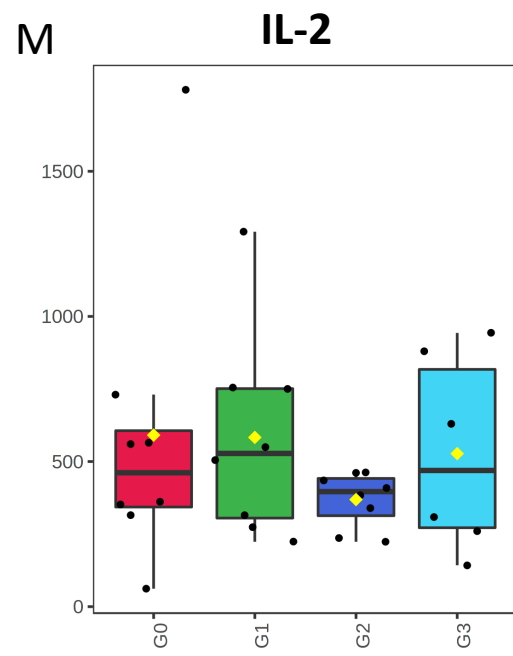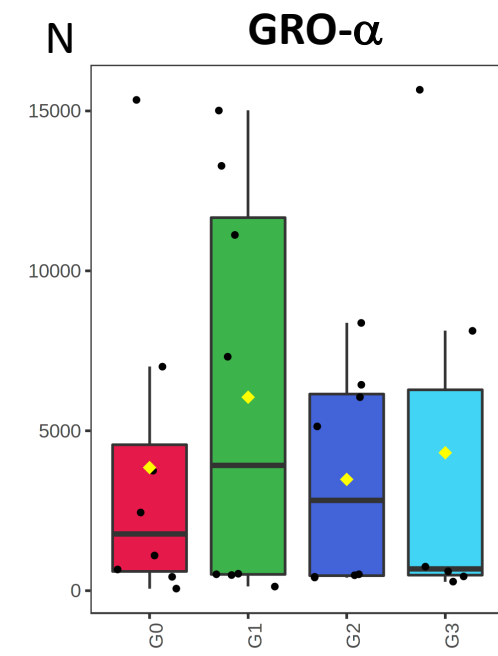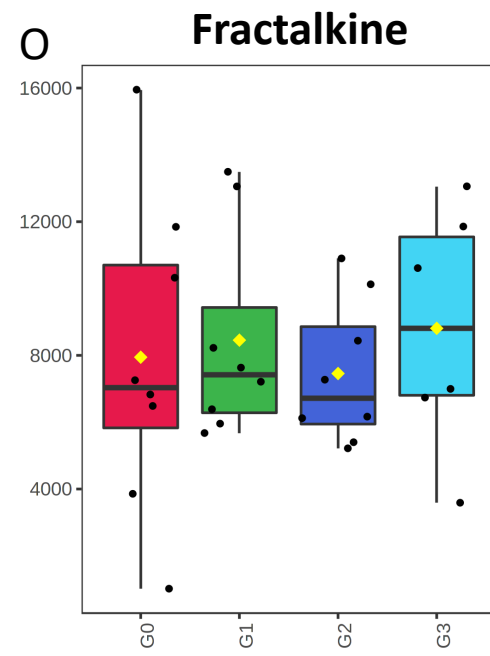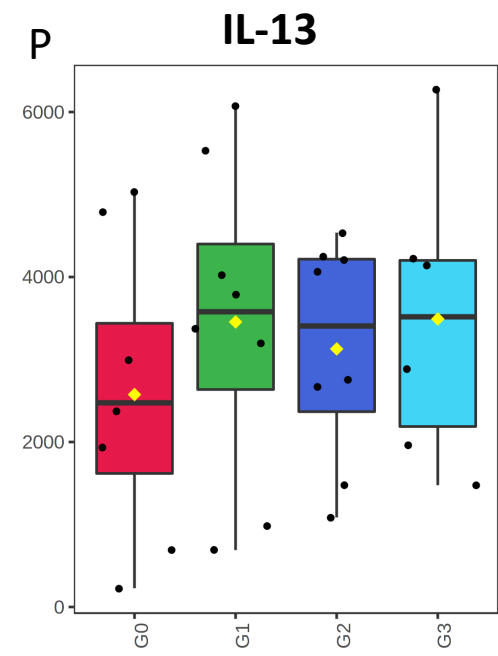

Q

LEPTIN

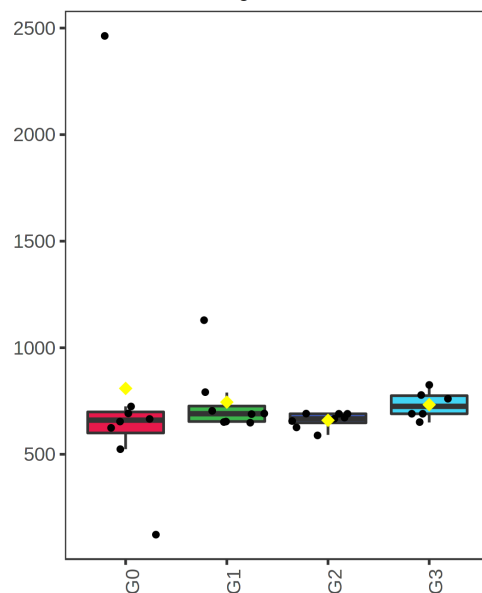

R

ENA-78

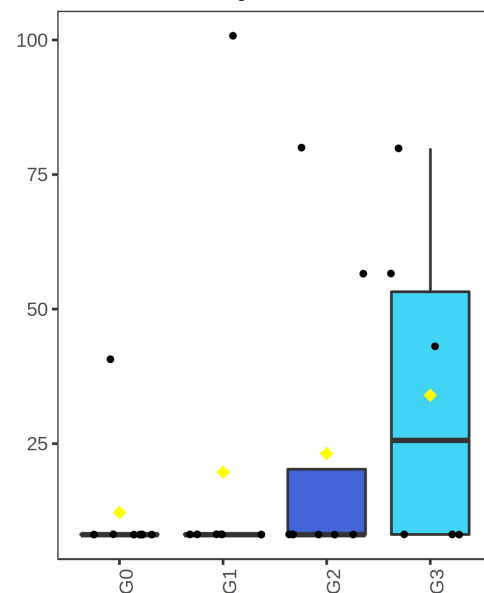

S

IL-5

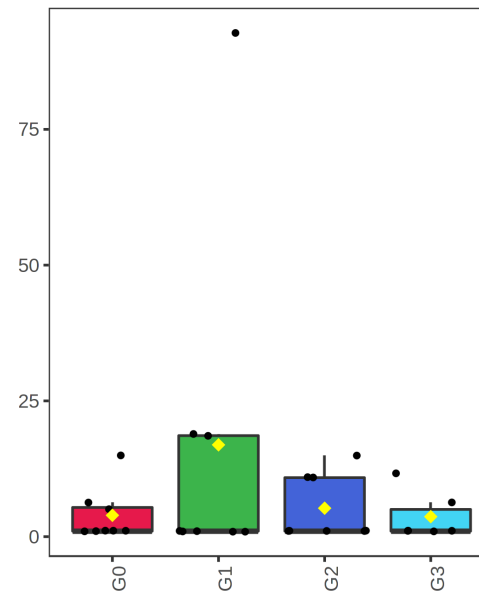

T

GSF

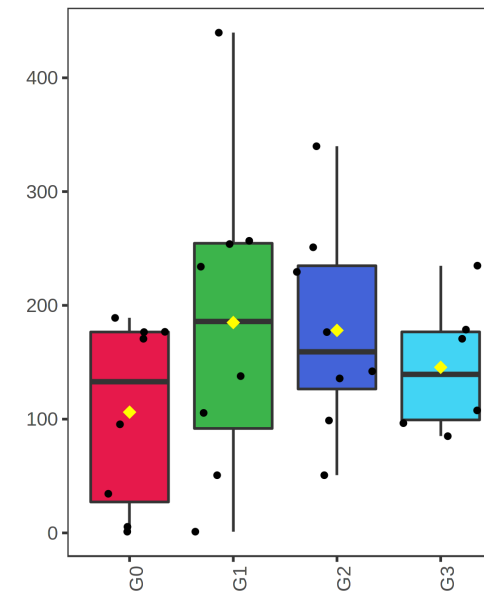

U

IL-12p70

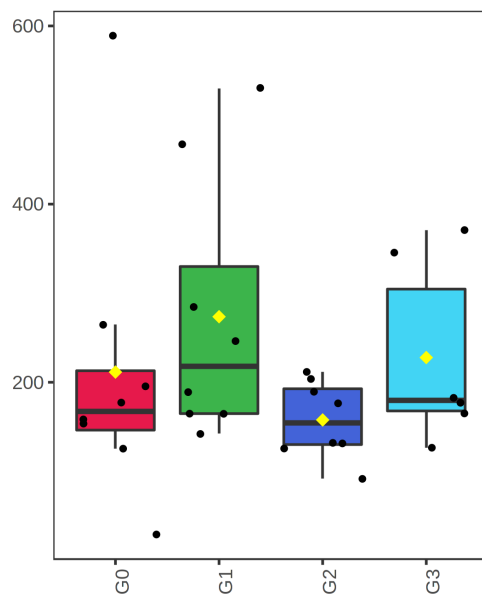

V

MCP-1

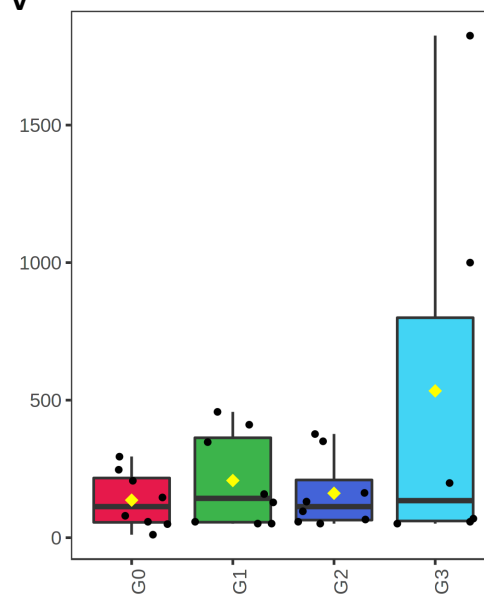

W

GM-CSF

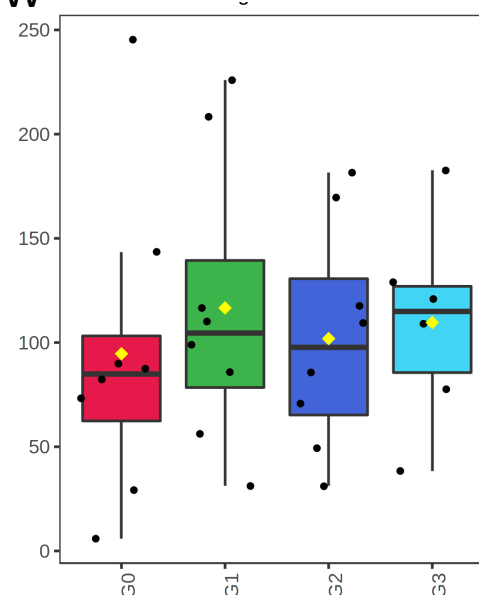

X

Eotaxin

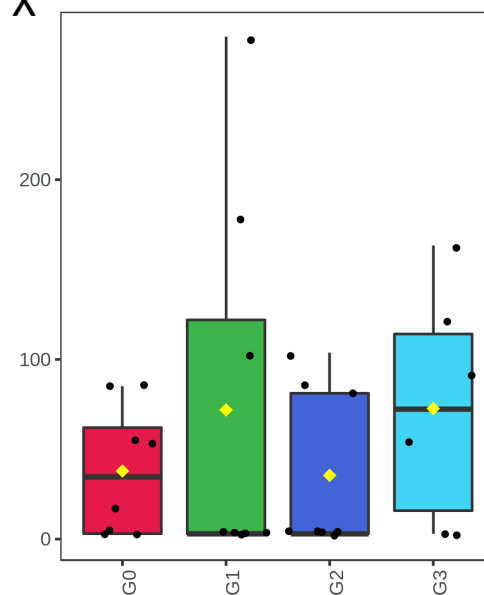

Supplement: Supplementary file 1 [file DataSheet_1.pdf]

**(A)**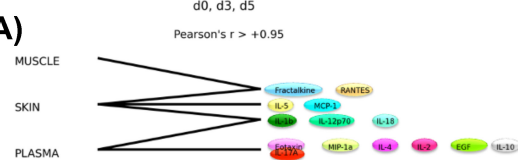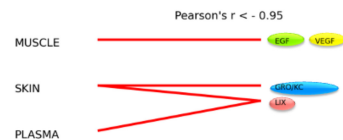**(D)**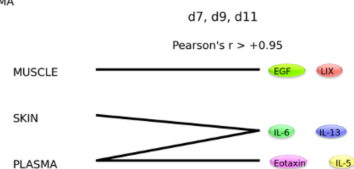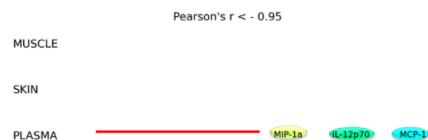**(G)**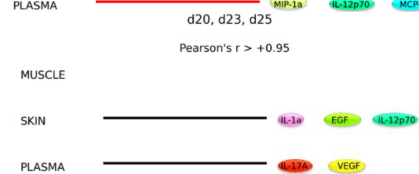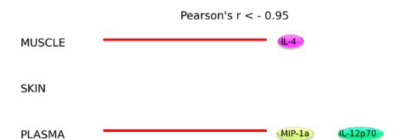**(B)**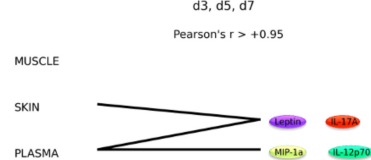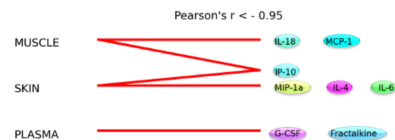**(E)**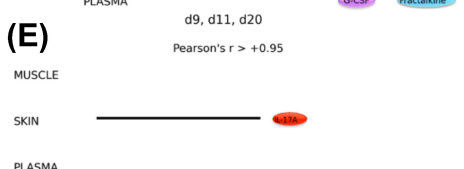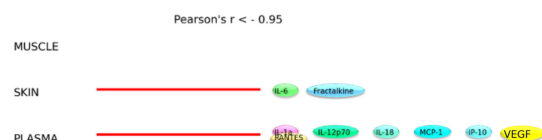**(H)**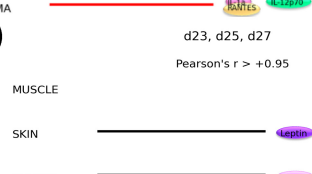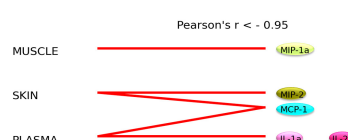**(C)**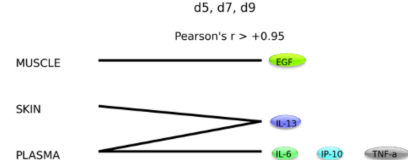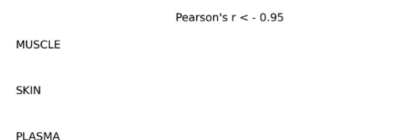**(F)**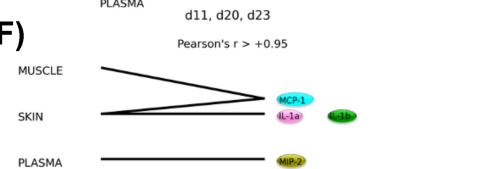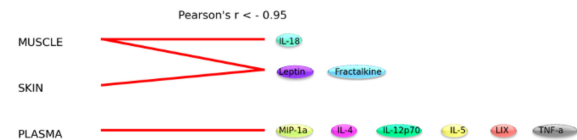**(I)**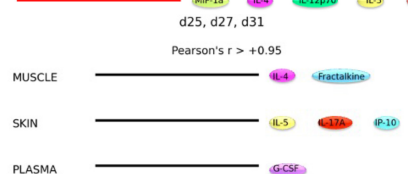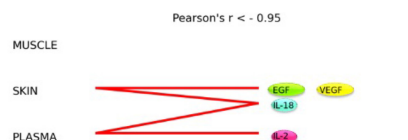

Supplement: Supplementary file 2 [file DataSheet_2.pdf]

**(A)**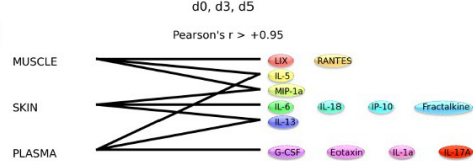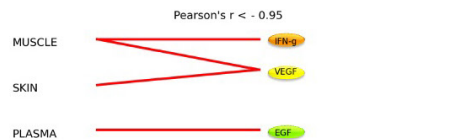**(D)**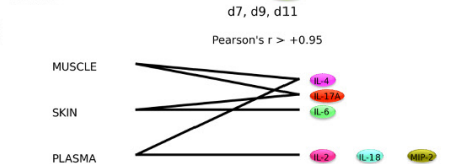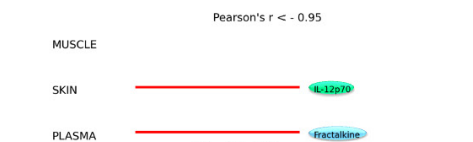**(G)**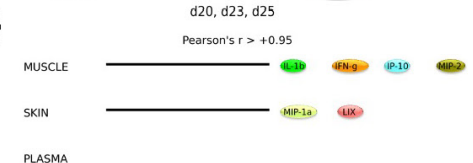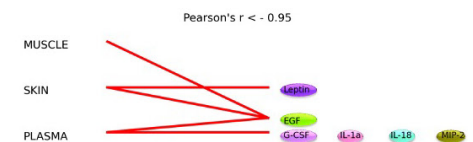**(B)**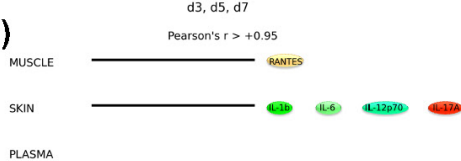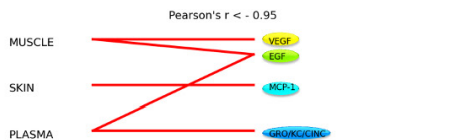**(E)**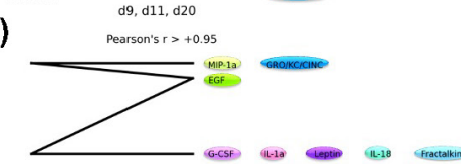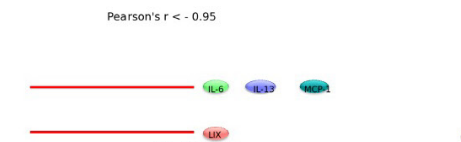**(H)**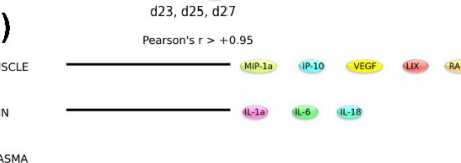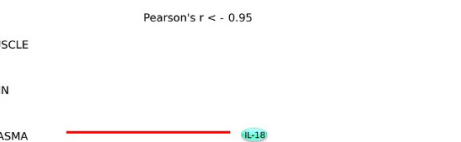**(C)**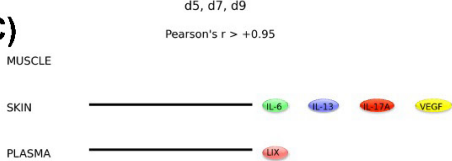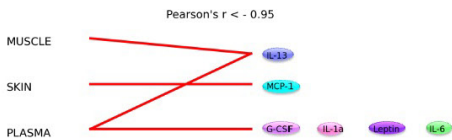**(F)**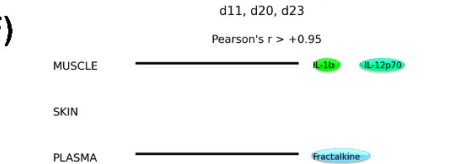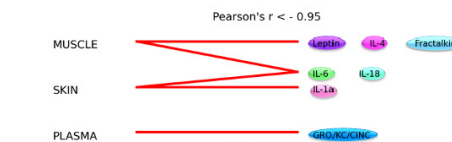**(I)**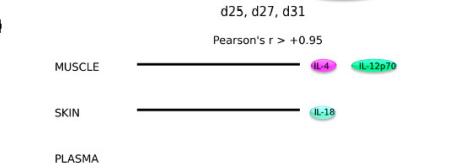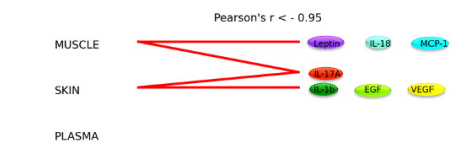

Supplement: Supplementary file 3 [file DataSheet_3.pdf]

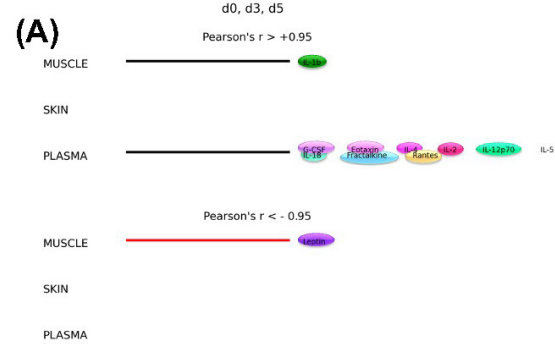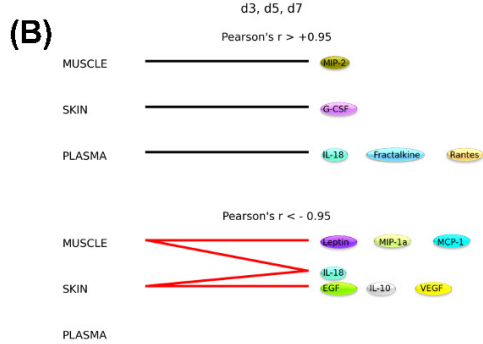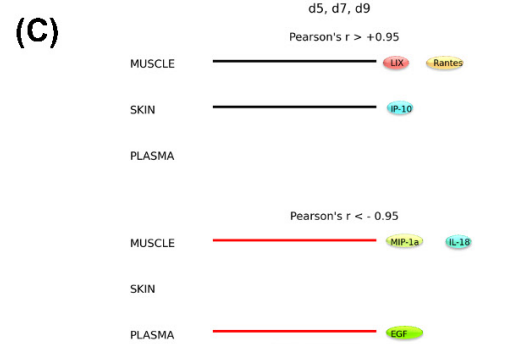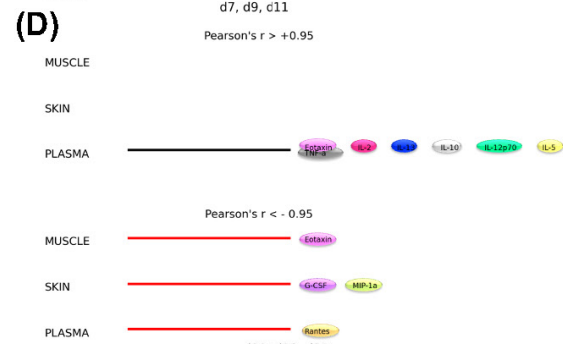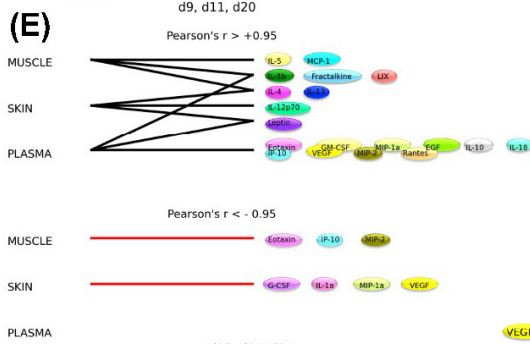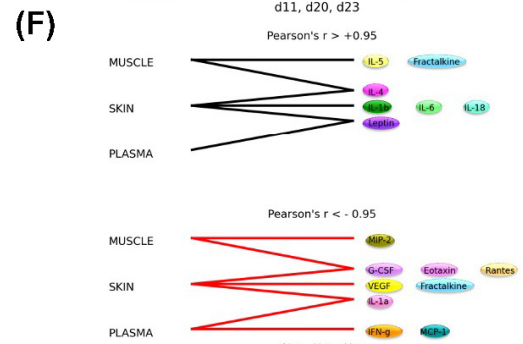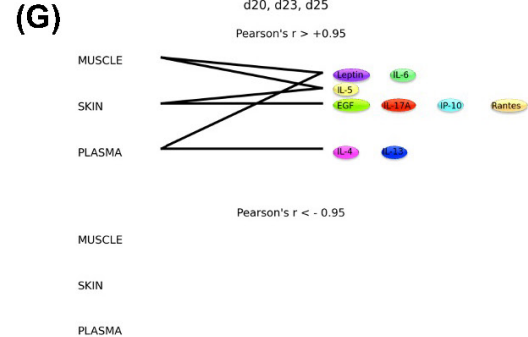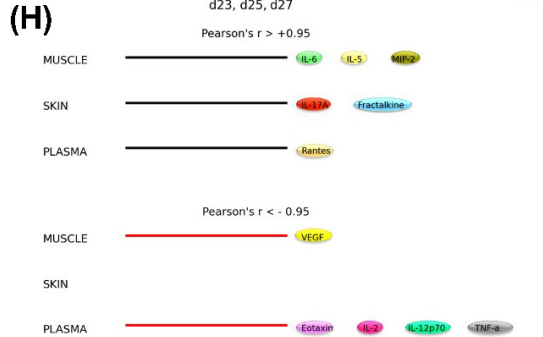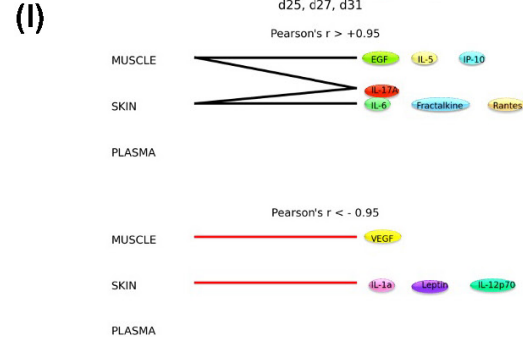

Supplement: Supplementary file 4 [file DataSheet_4.pdf]

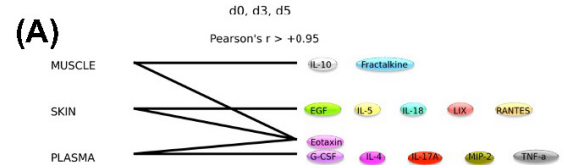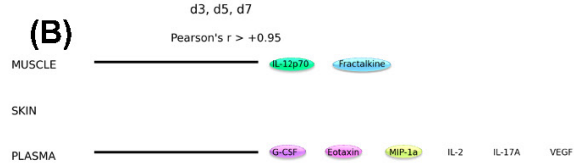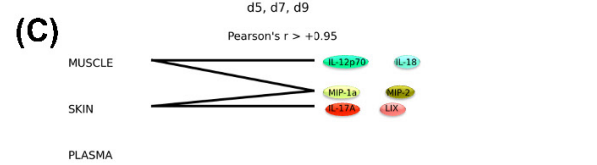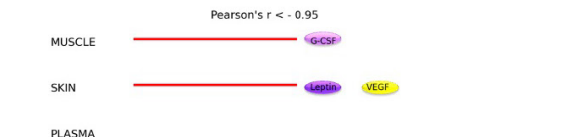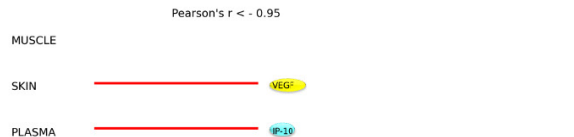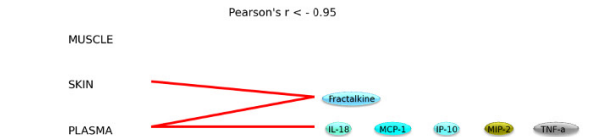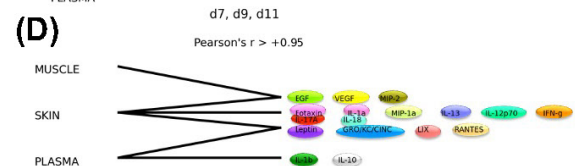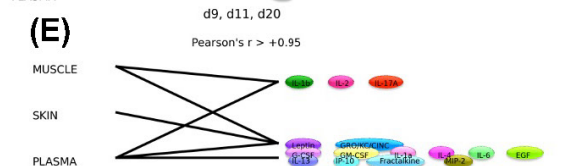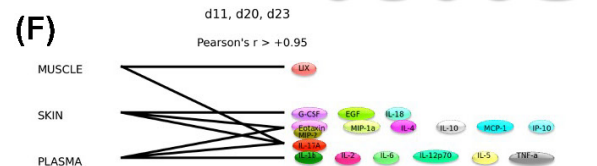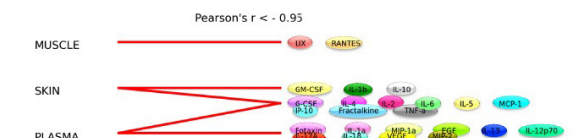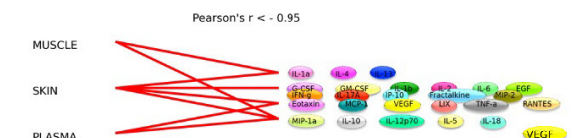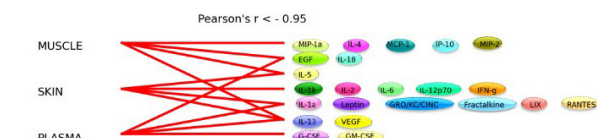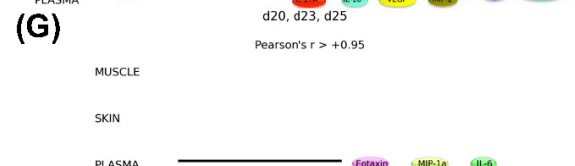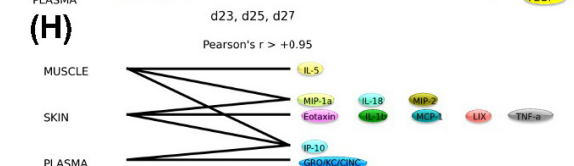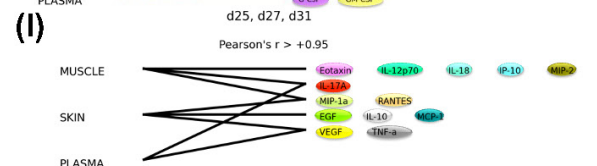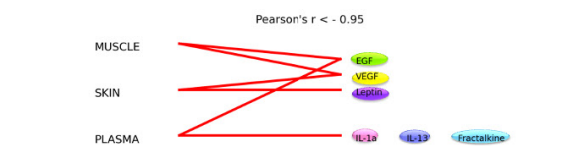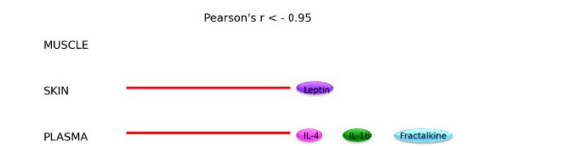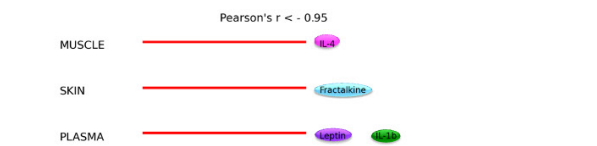

Supplement: Supplementary file 5 [file DataSheet_5.pdf]

1.

**G-CSF**

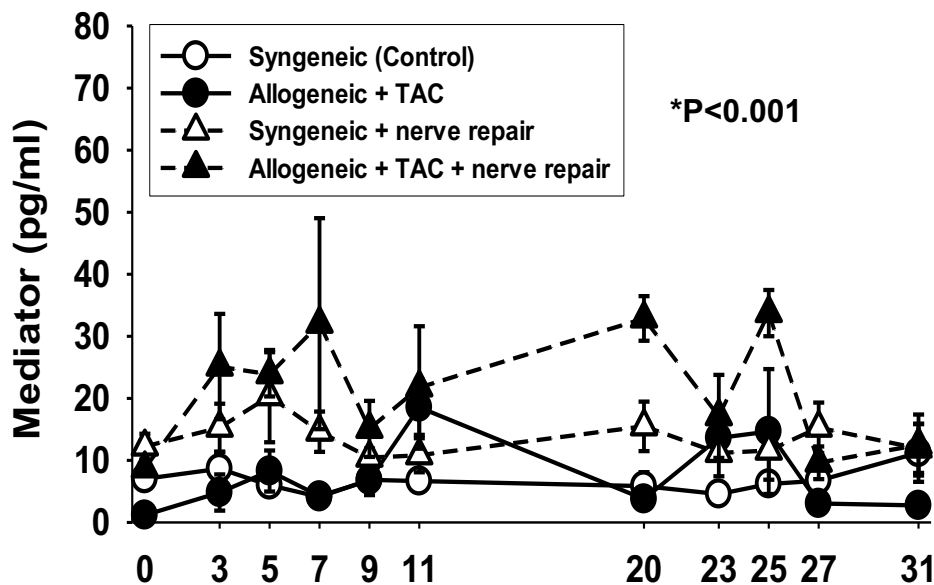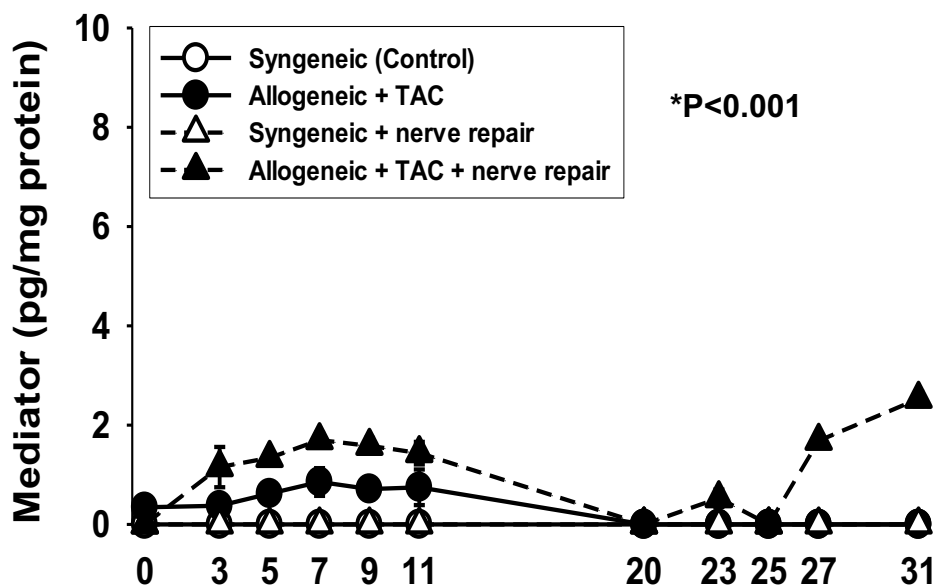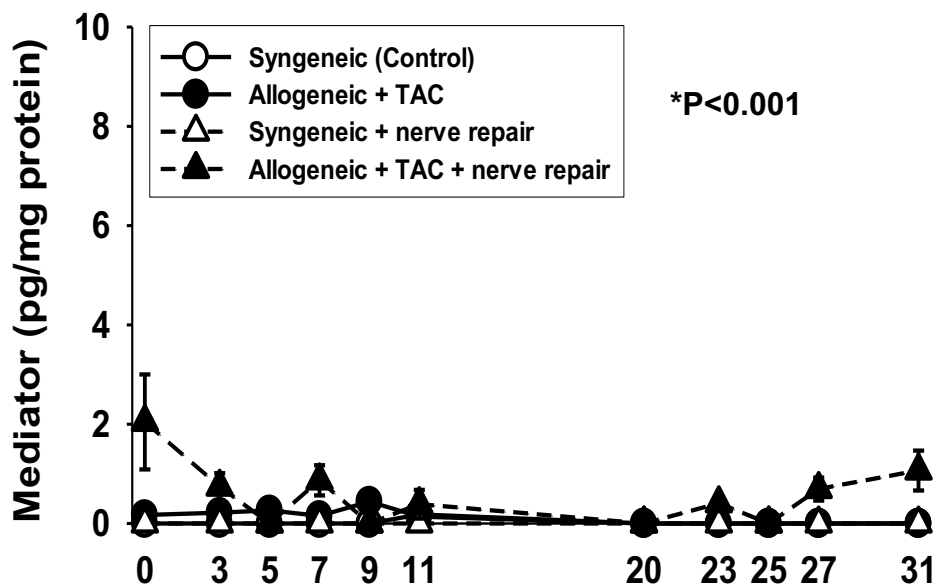

2.

# Eotaxin

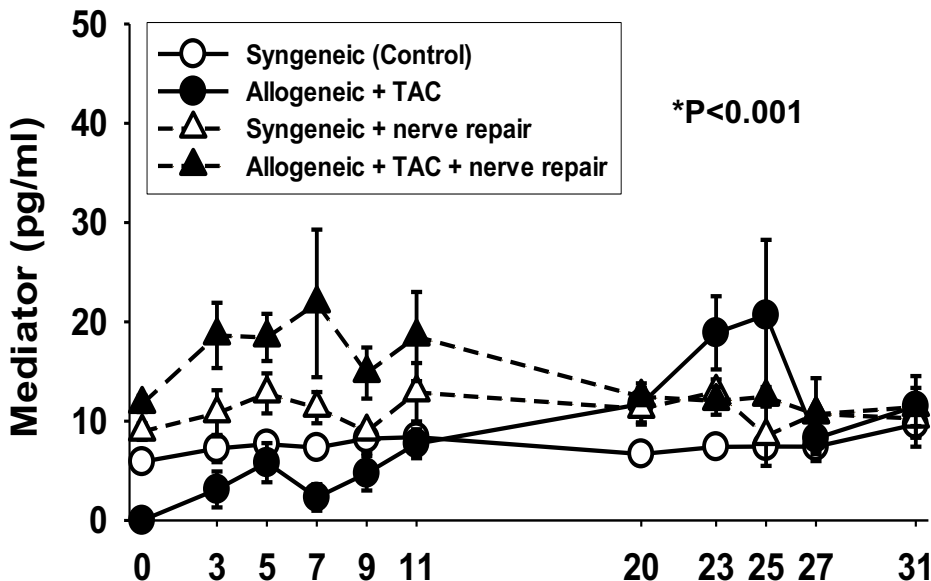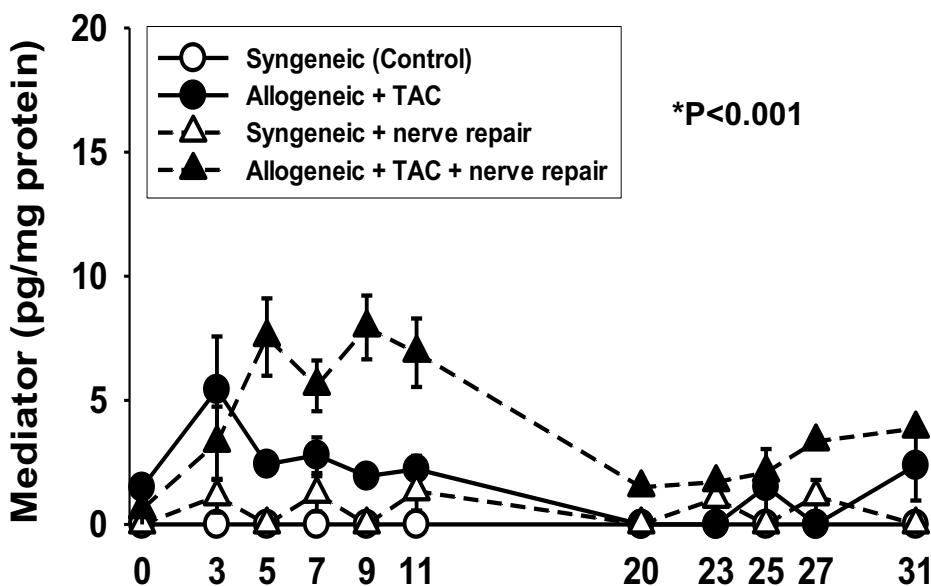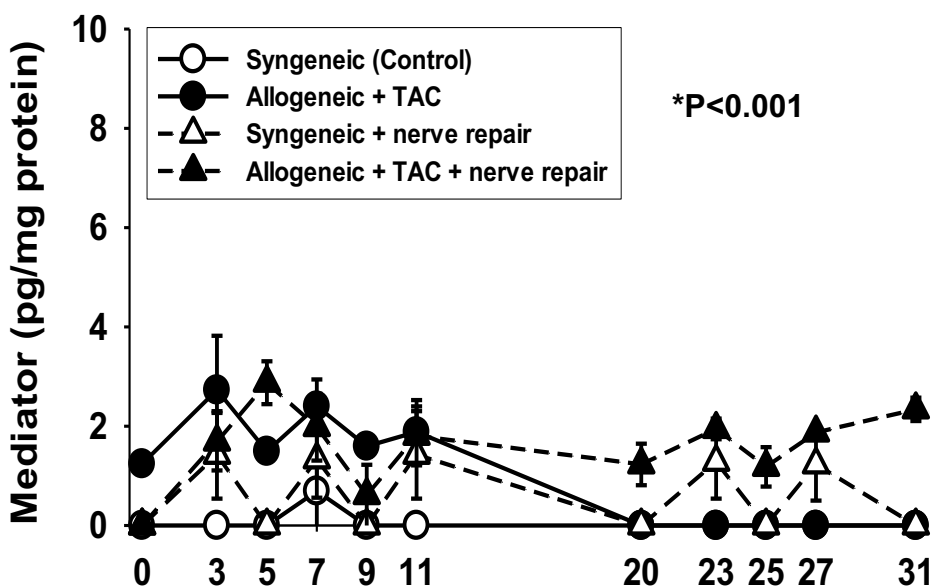

3.

# GM-CSF

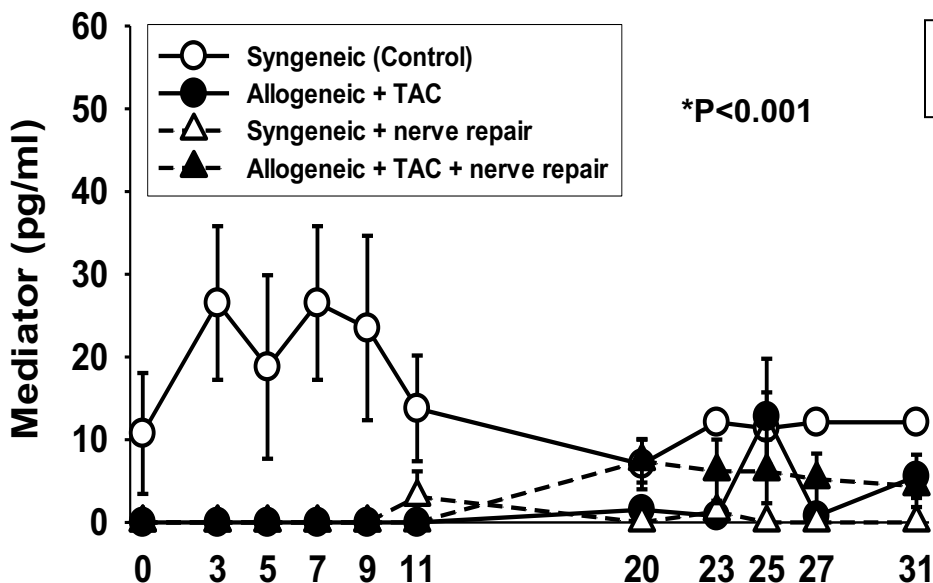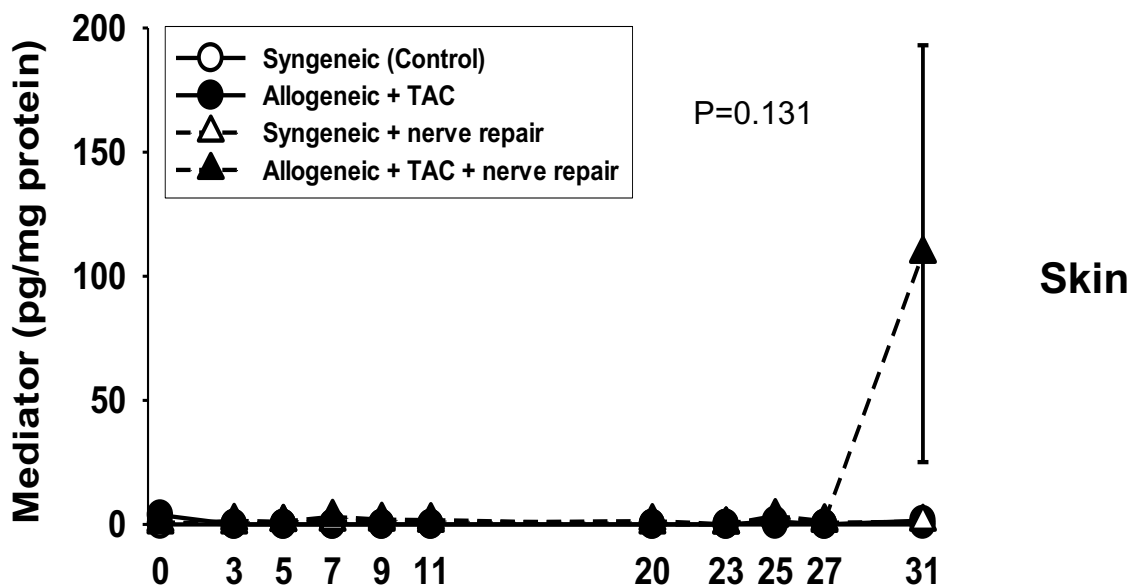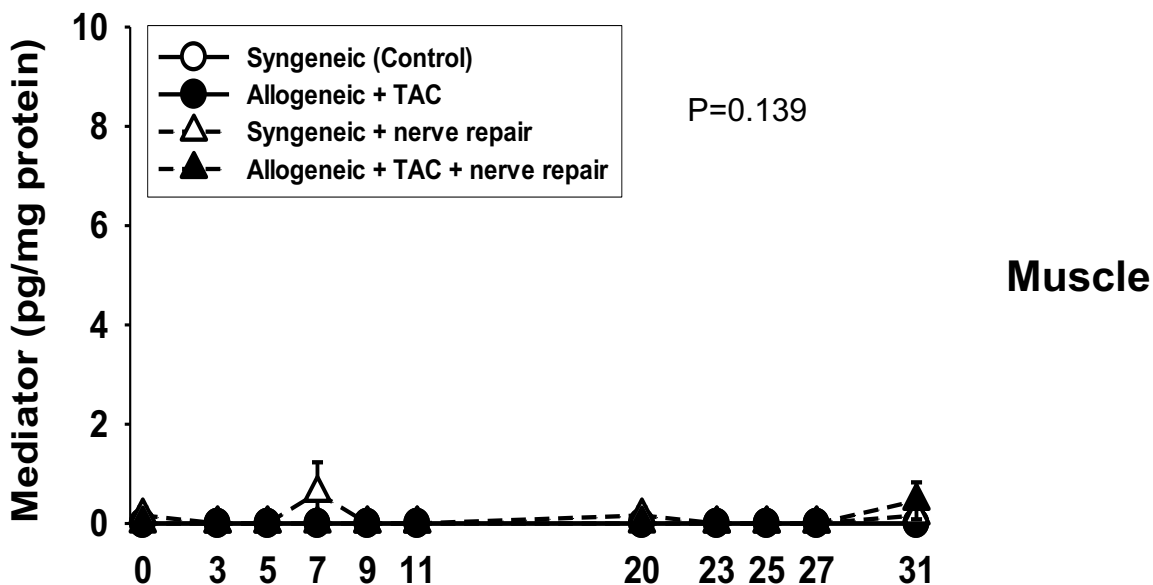

4.

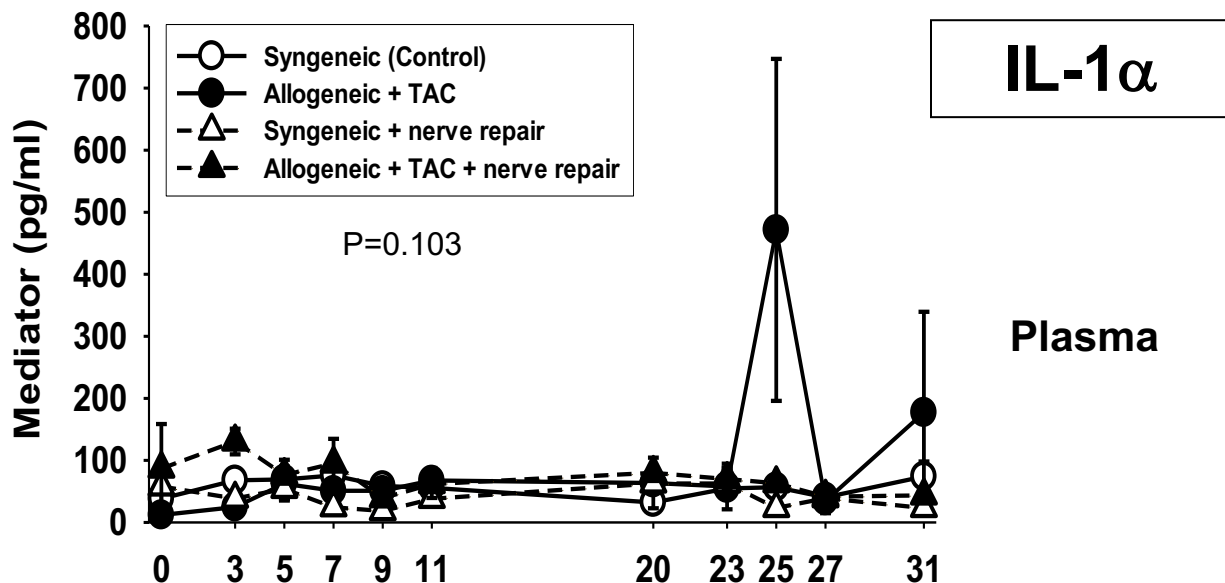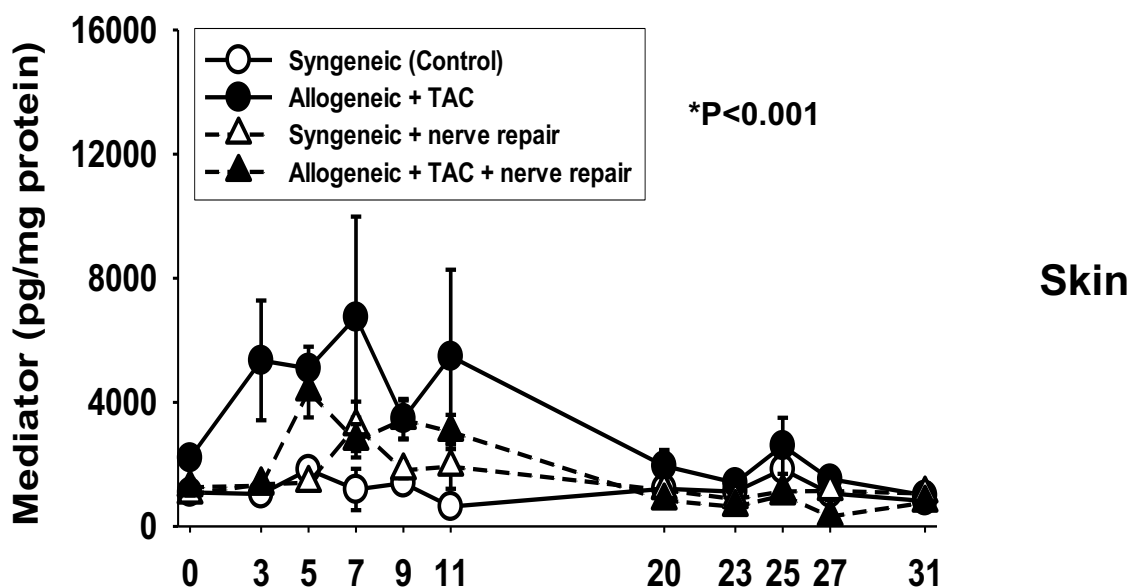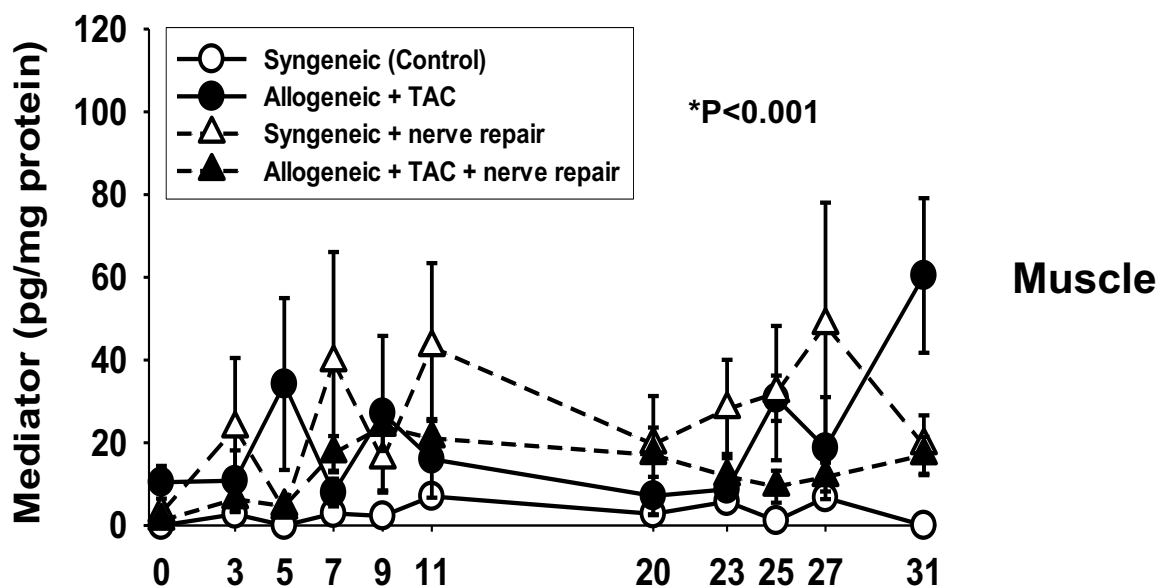

5.

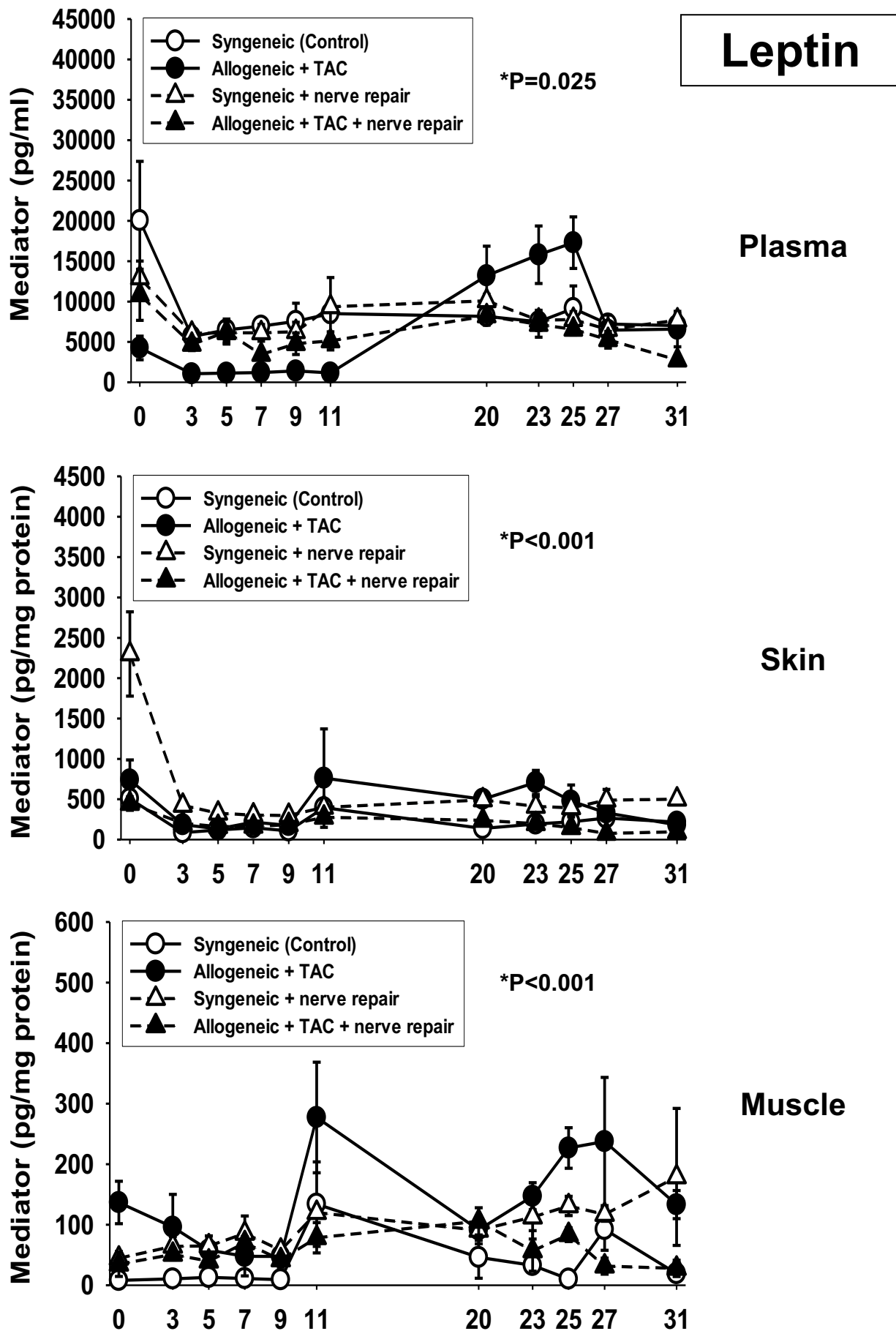

6.

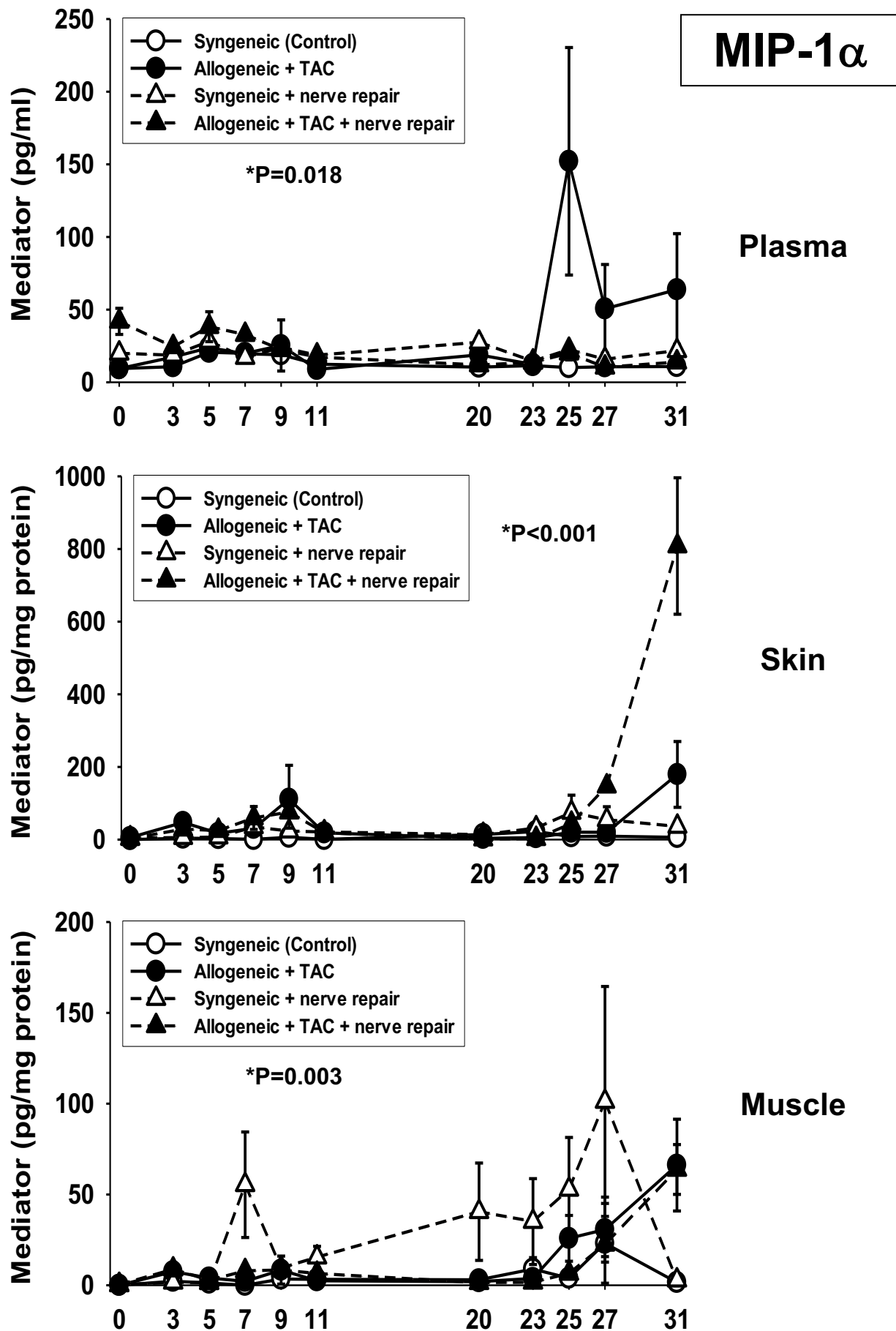

7.

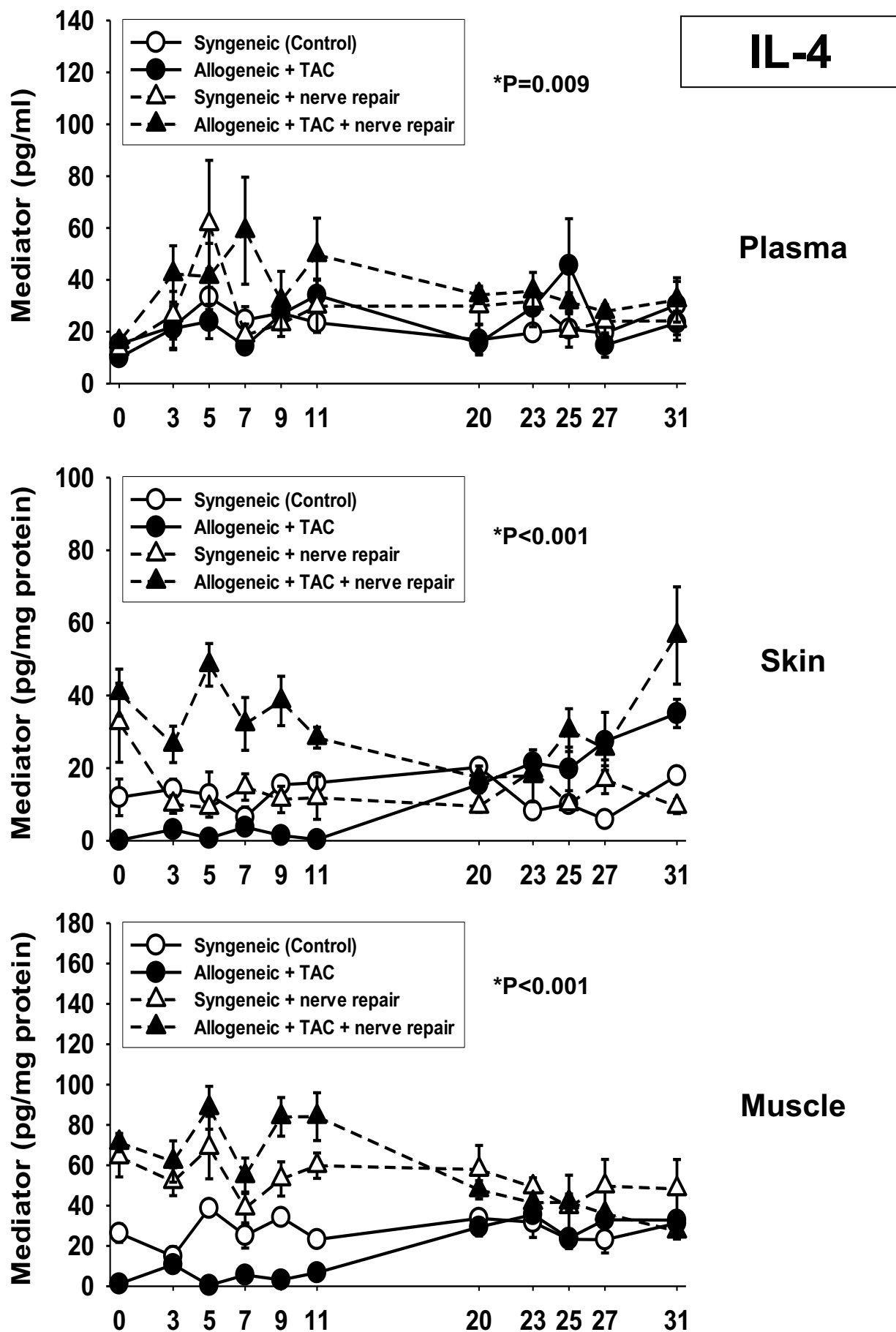

8.

**IL-1 $\beta$**

P=0.576

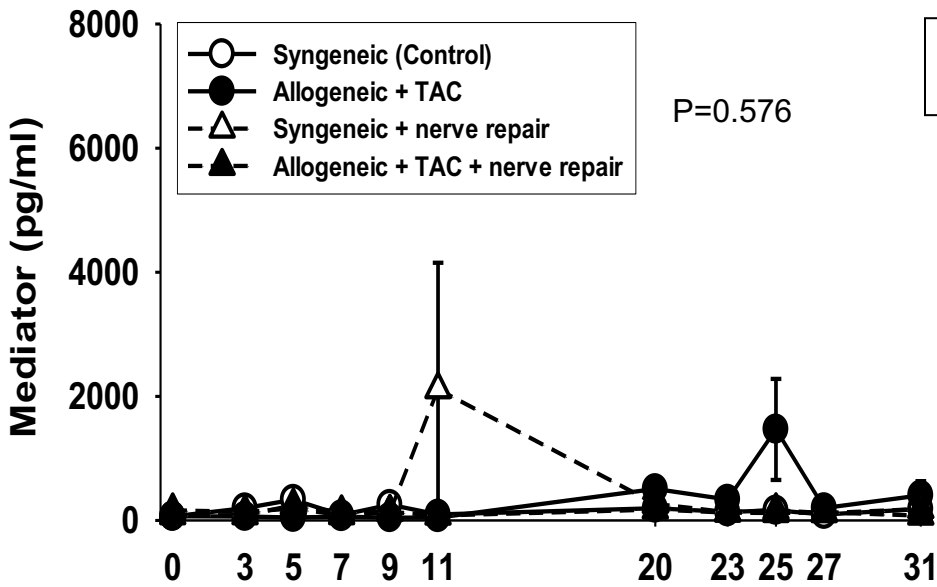

**Plasma**

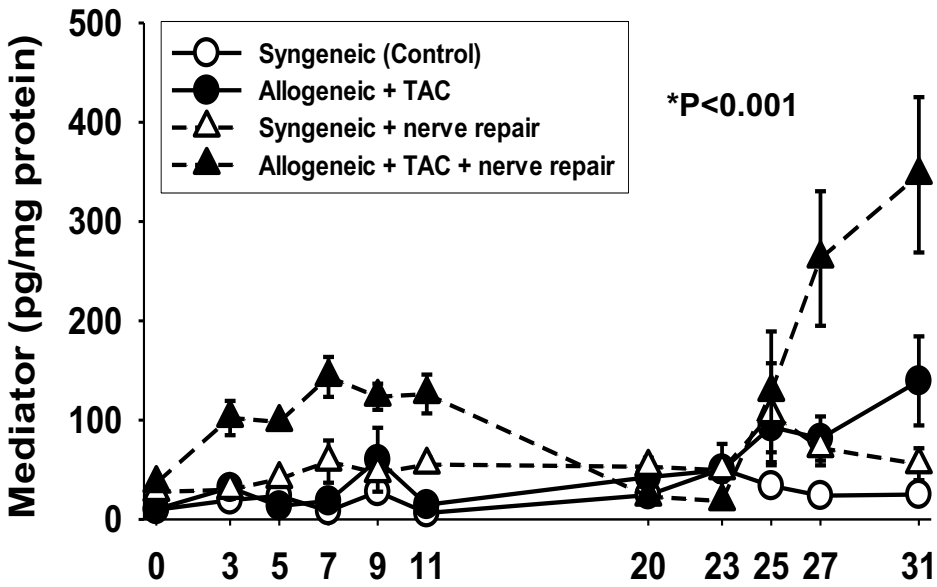

**Skin**

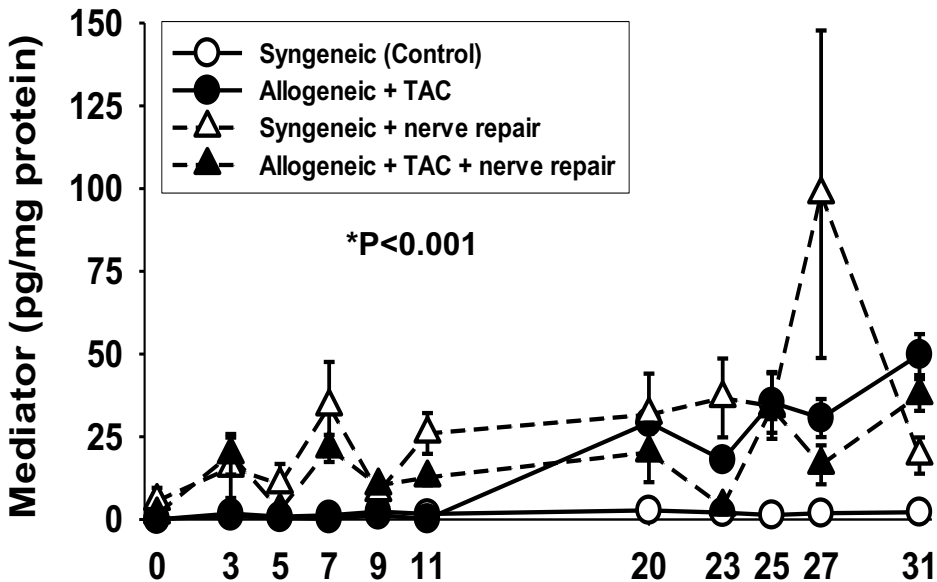

**Muscle**

9.

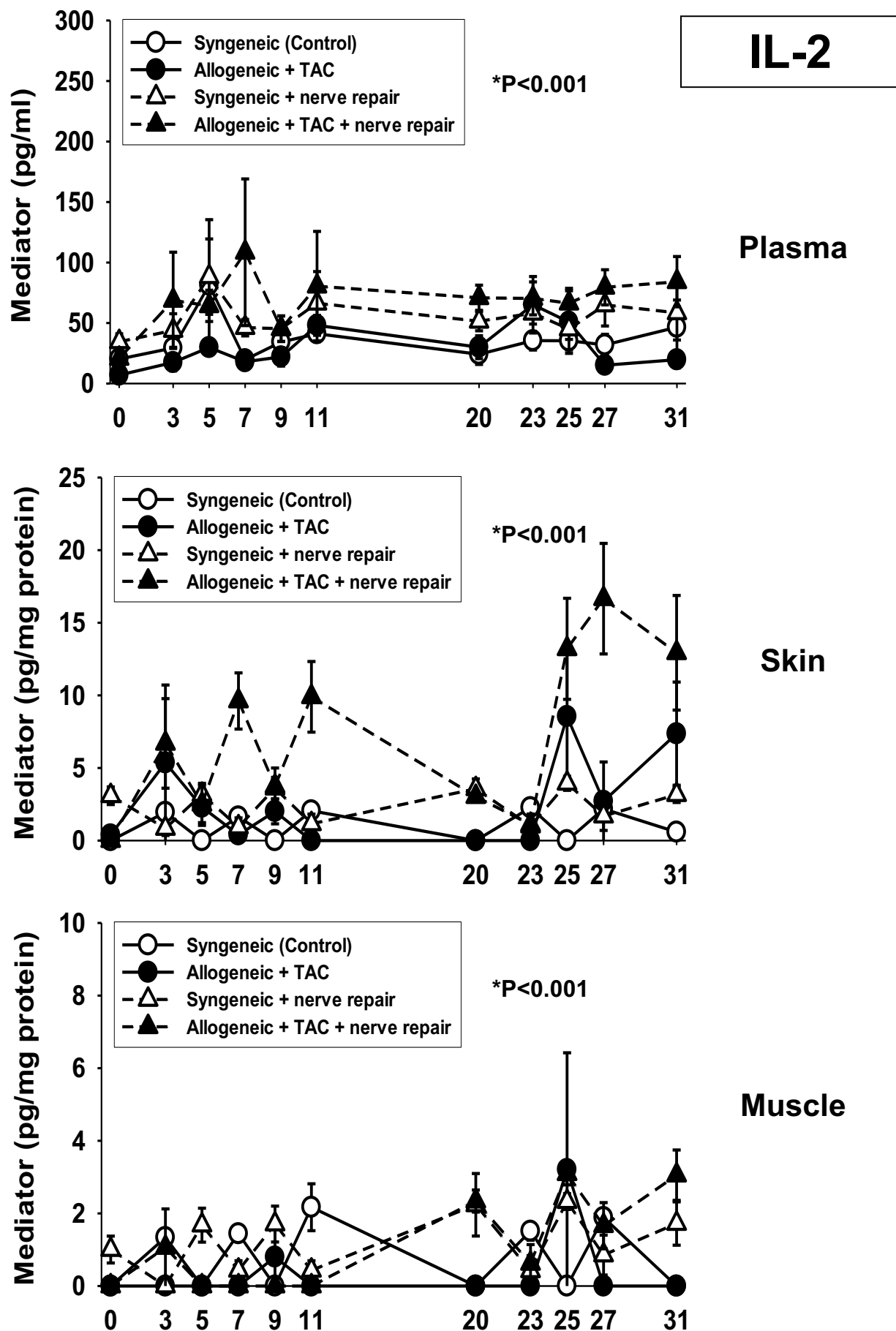

10.

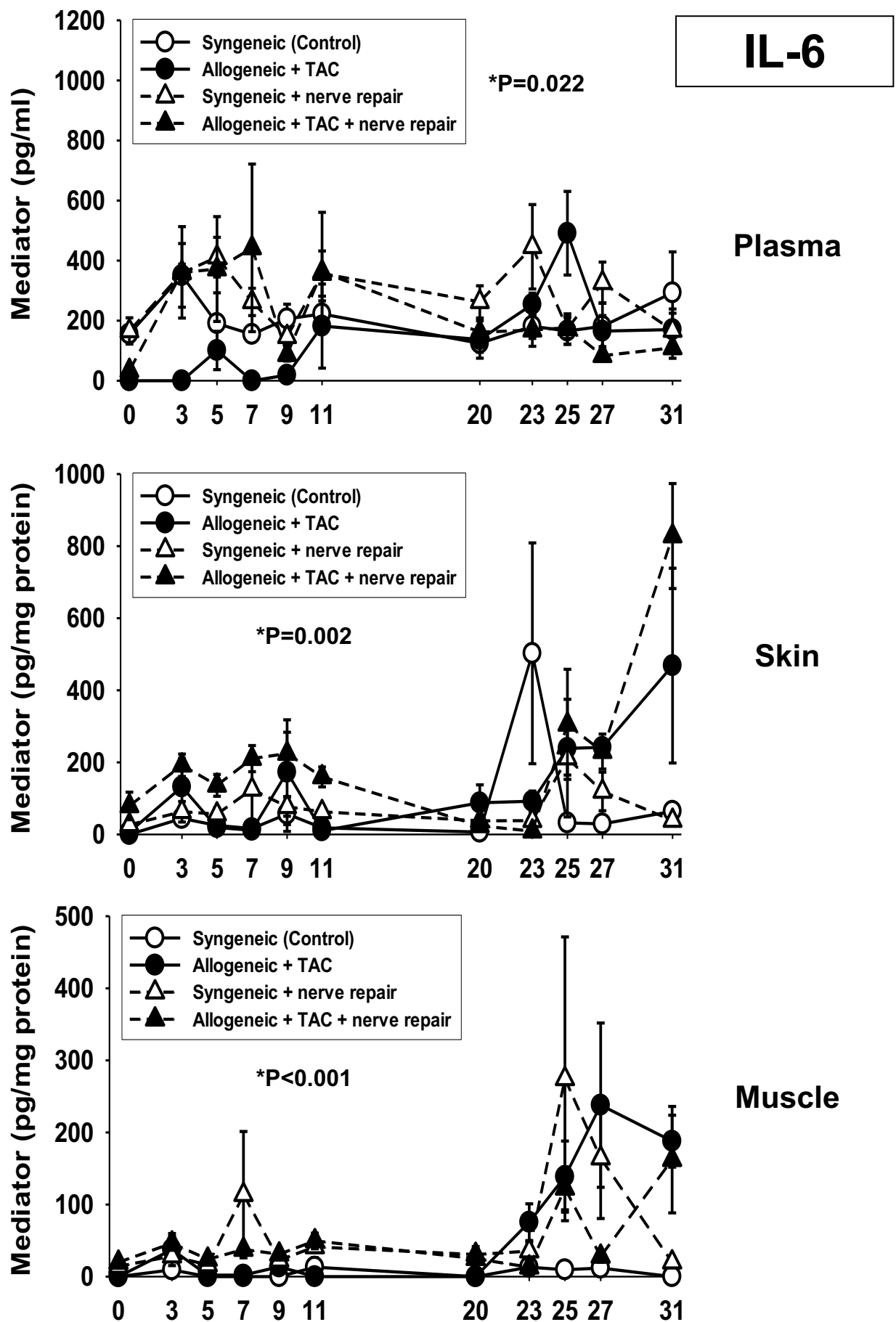

11.

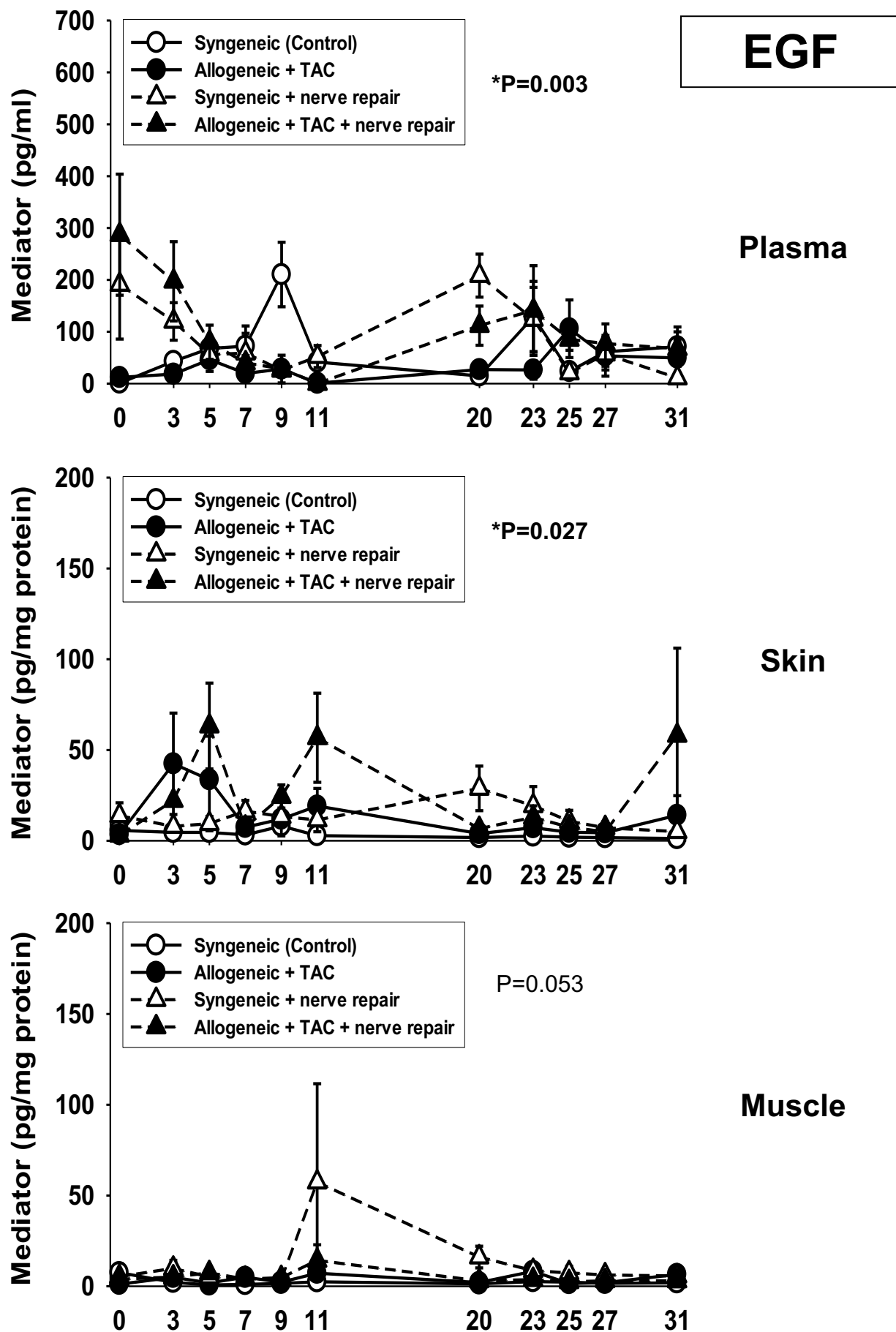

12.

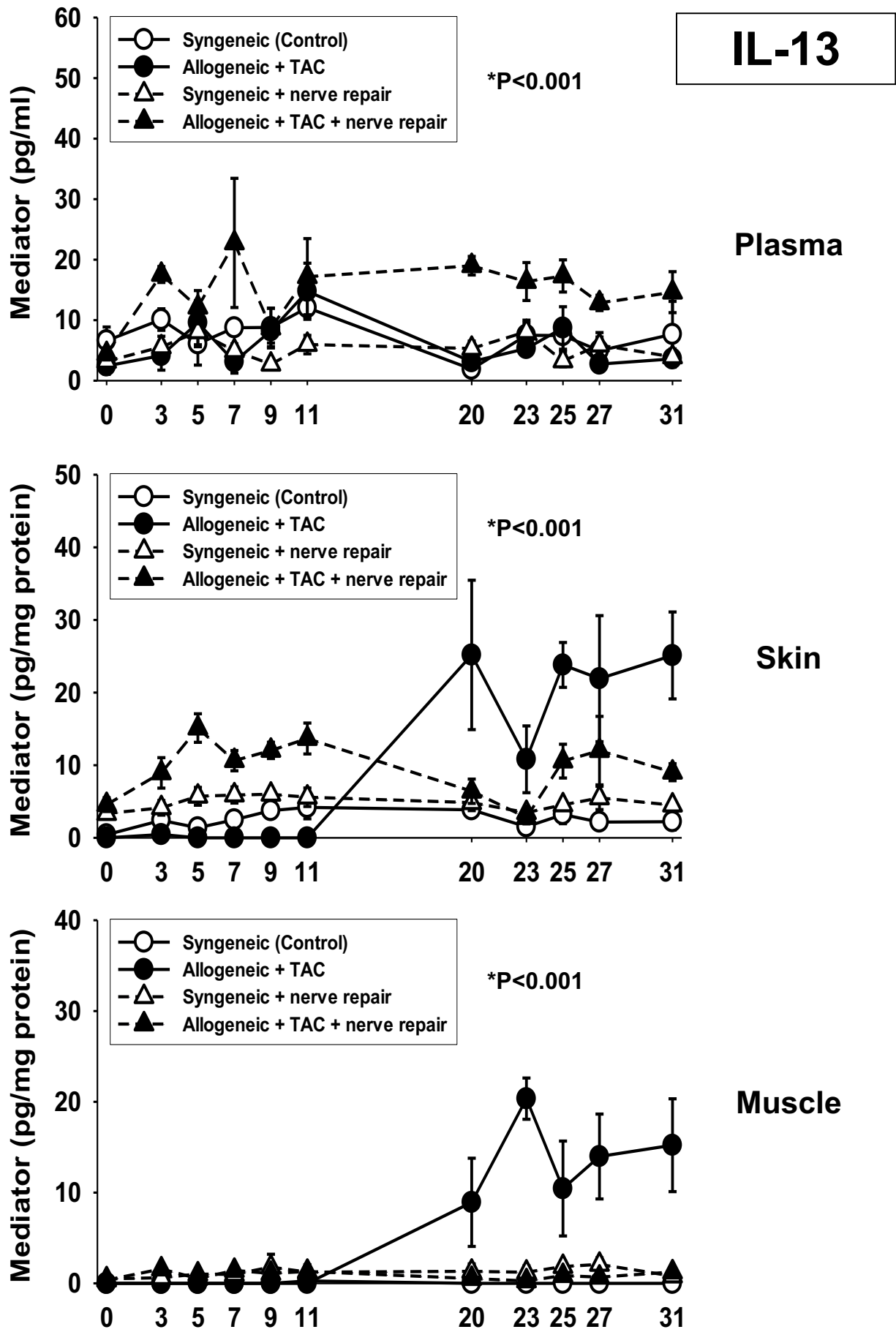

13.

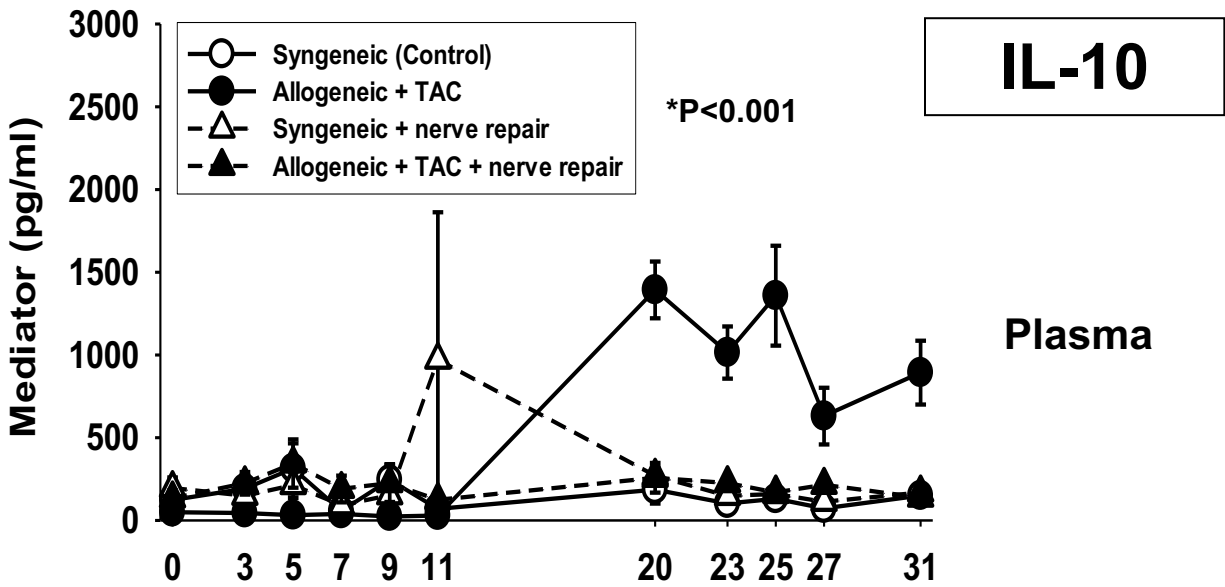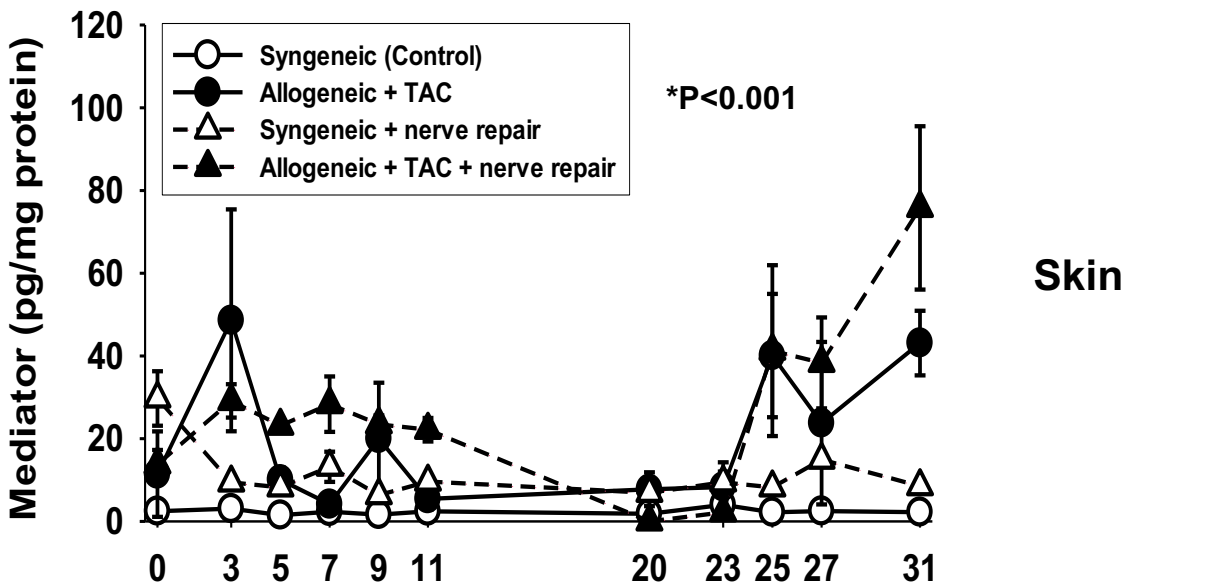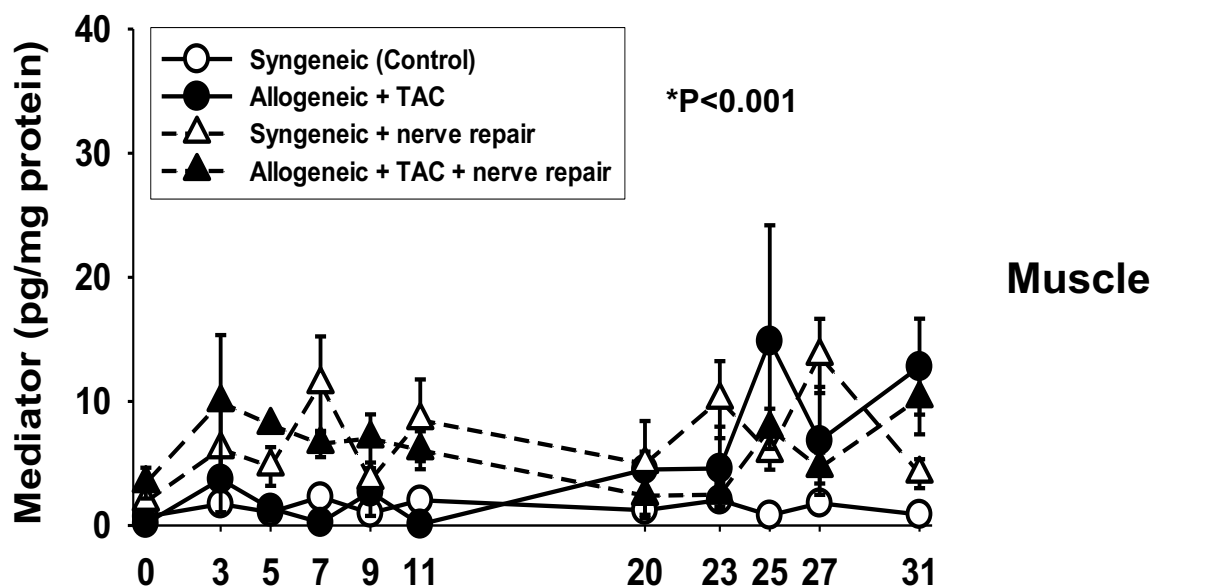

14.

IL-12p70

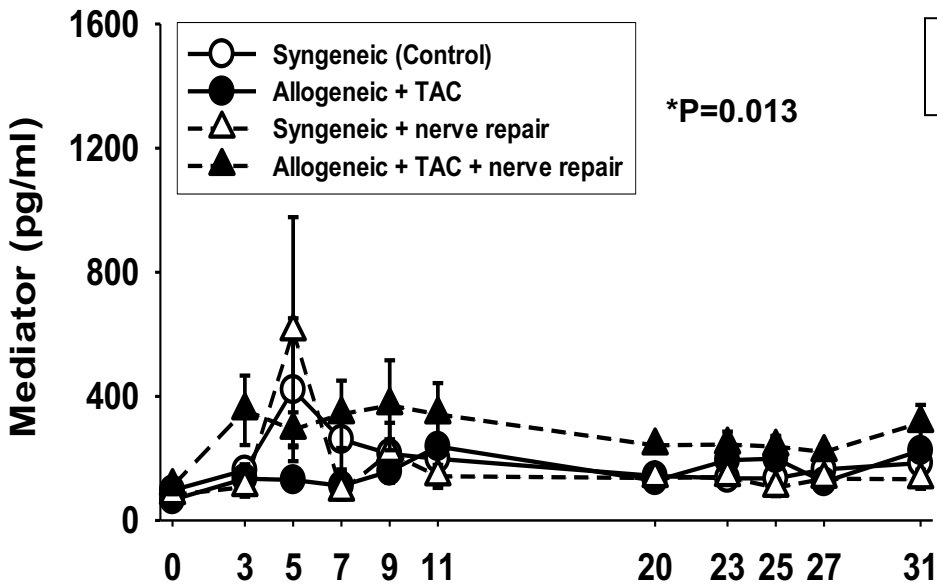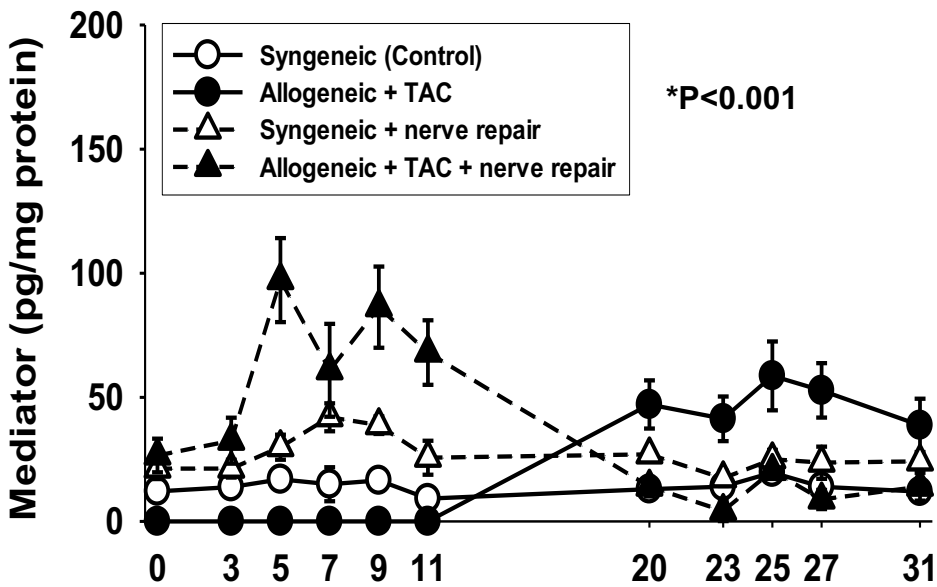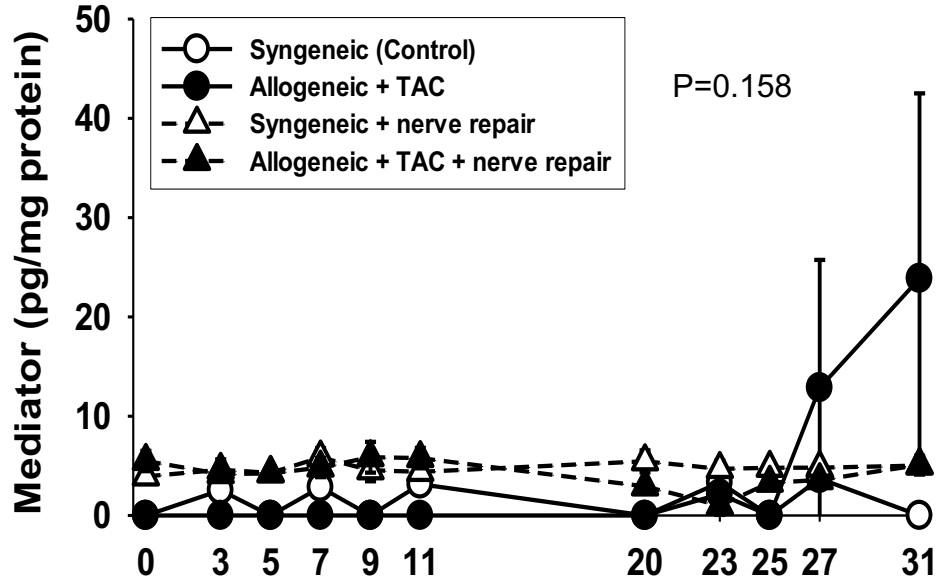

15.

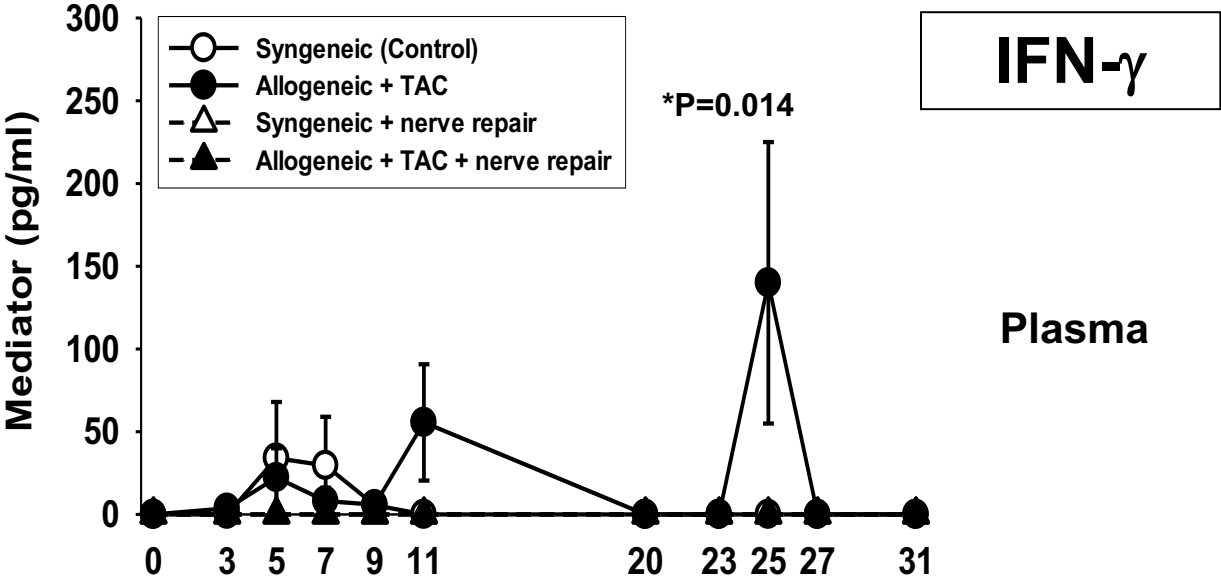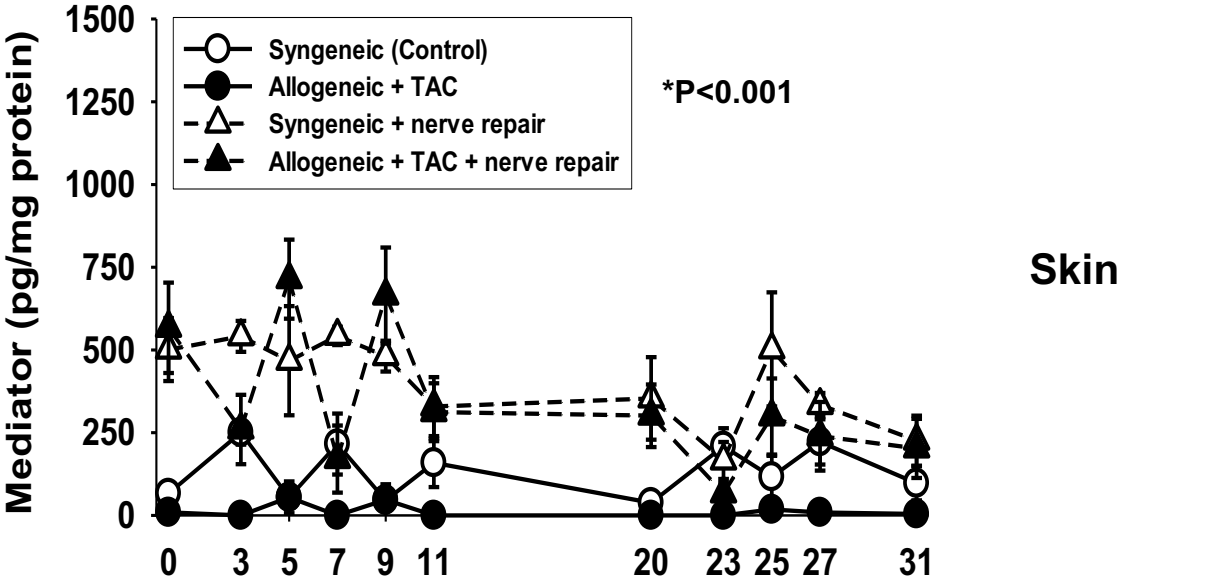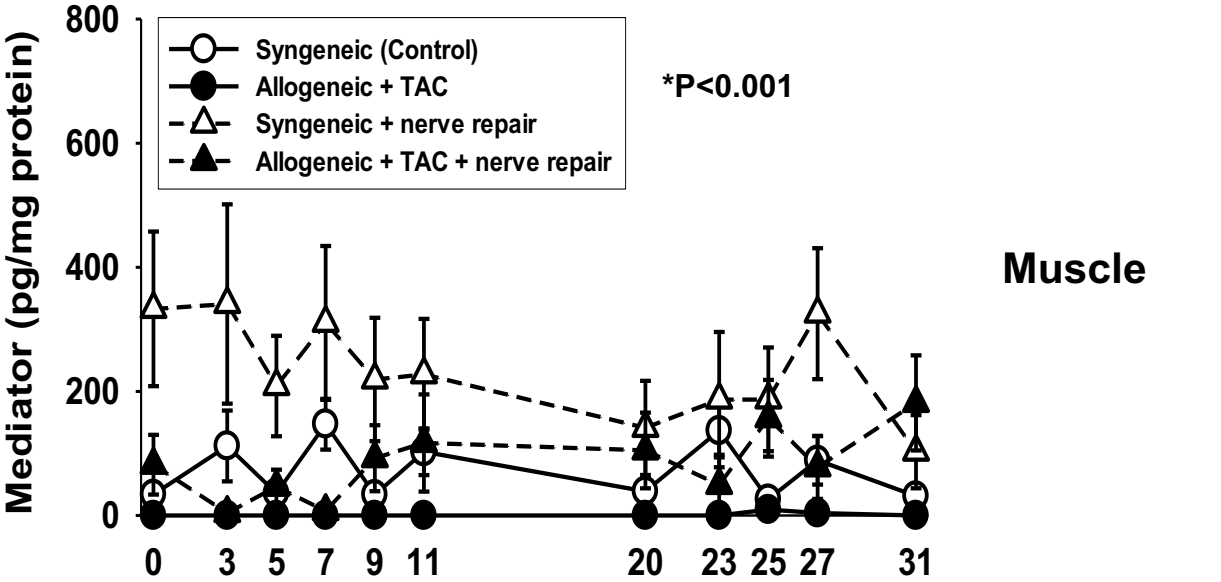

16.

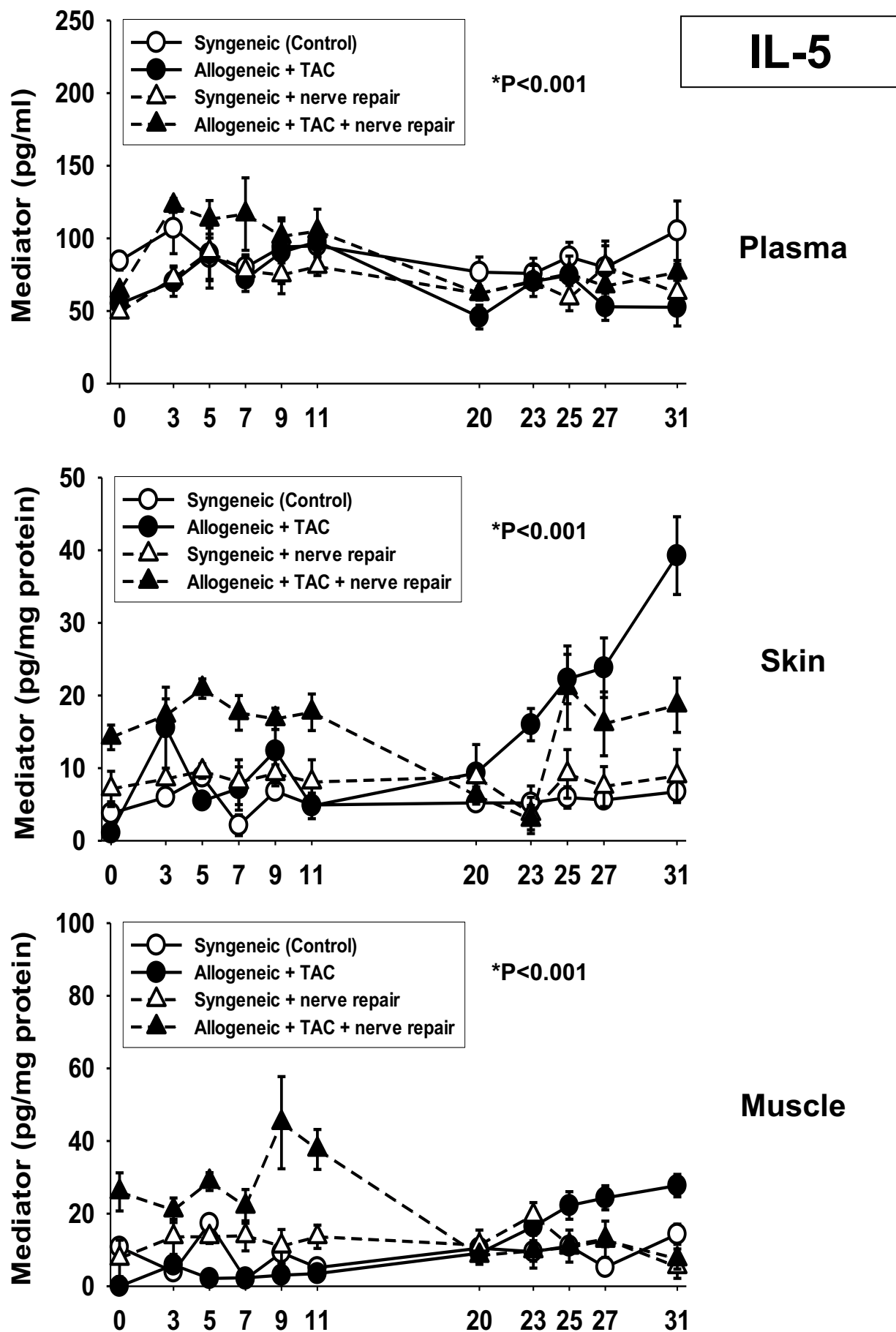

17.

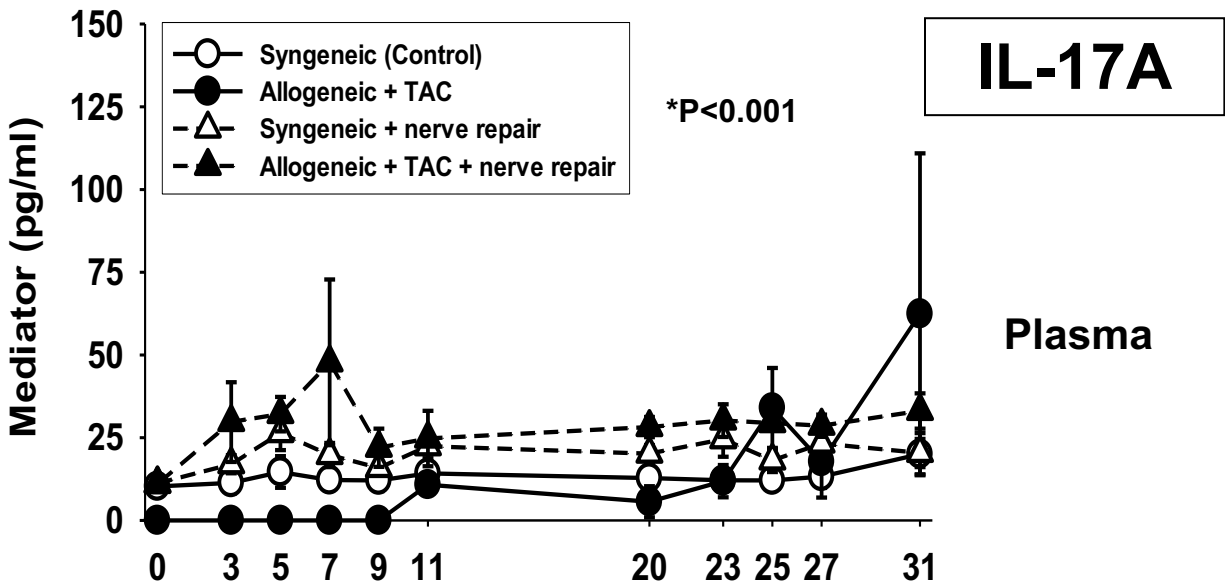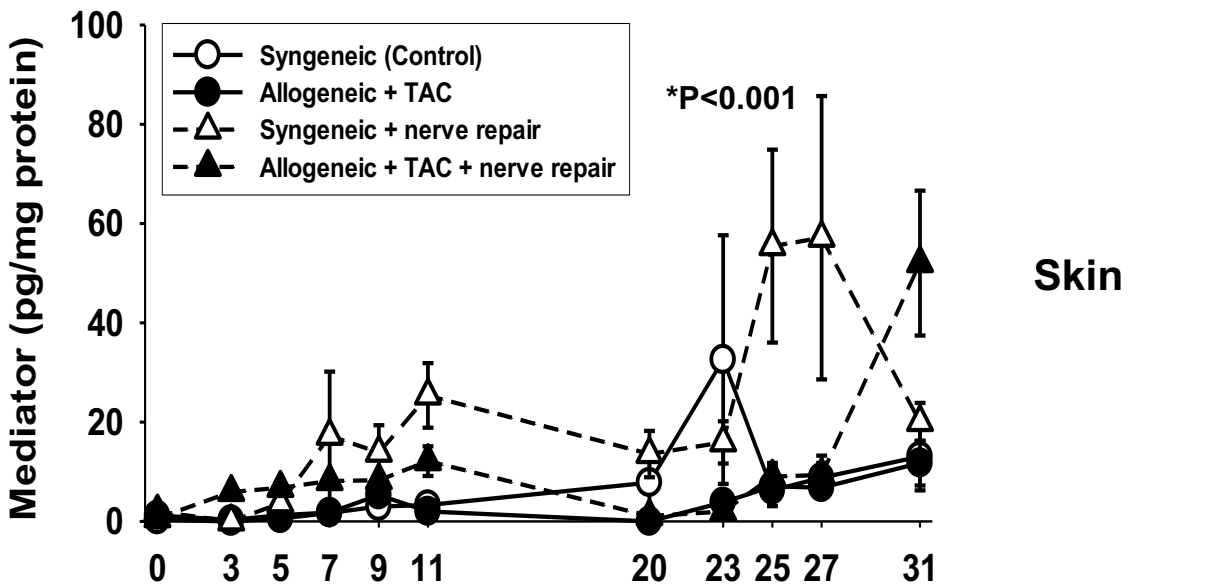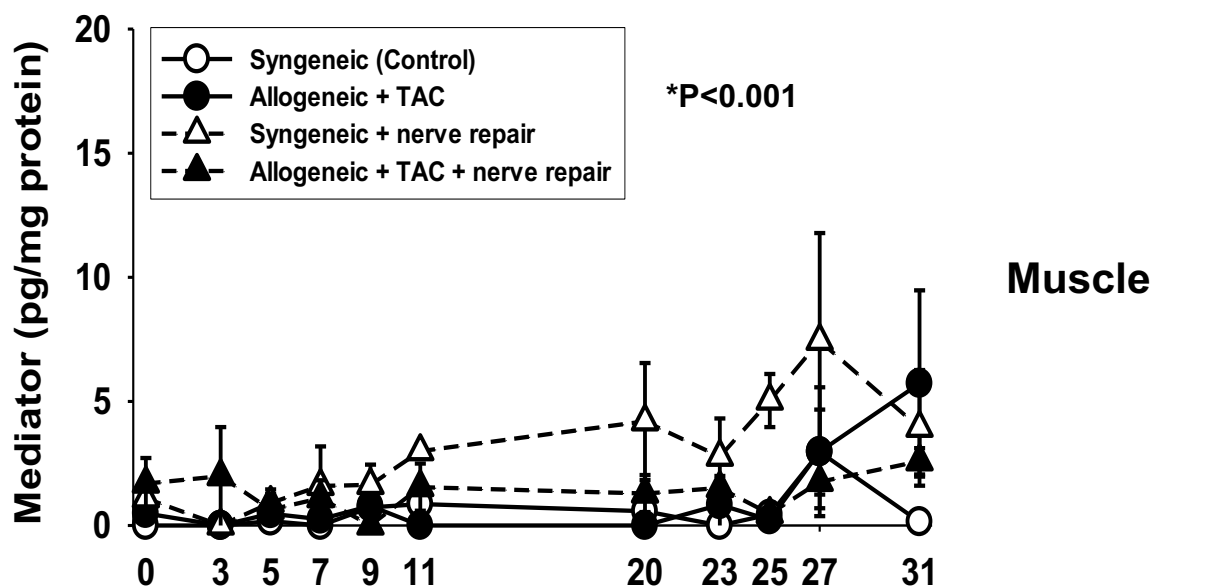

18.

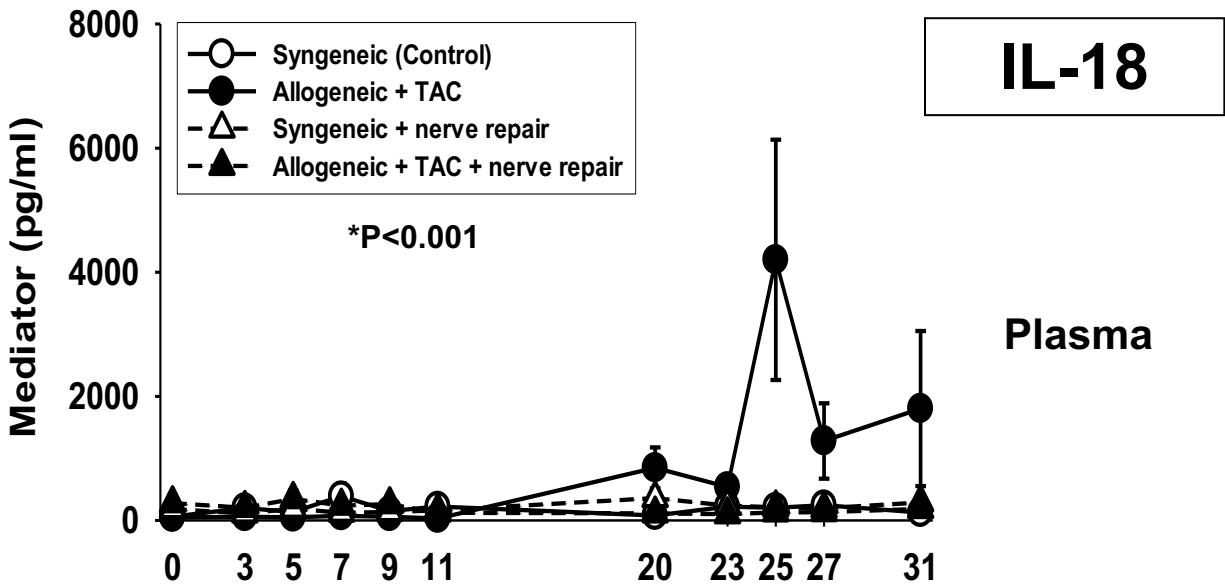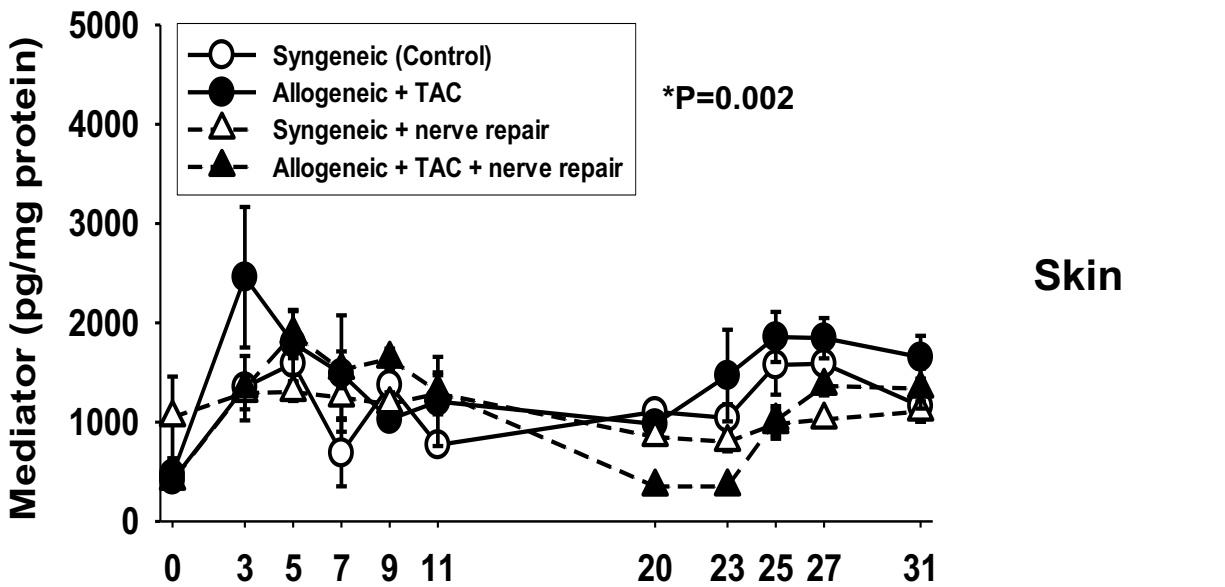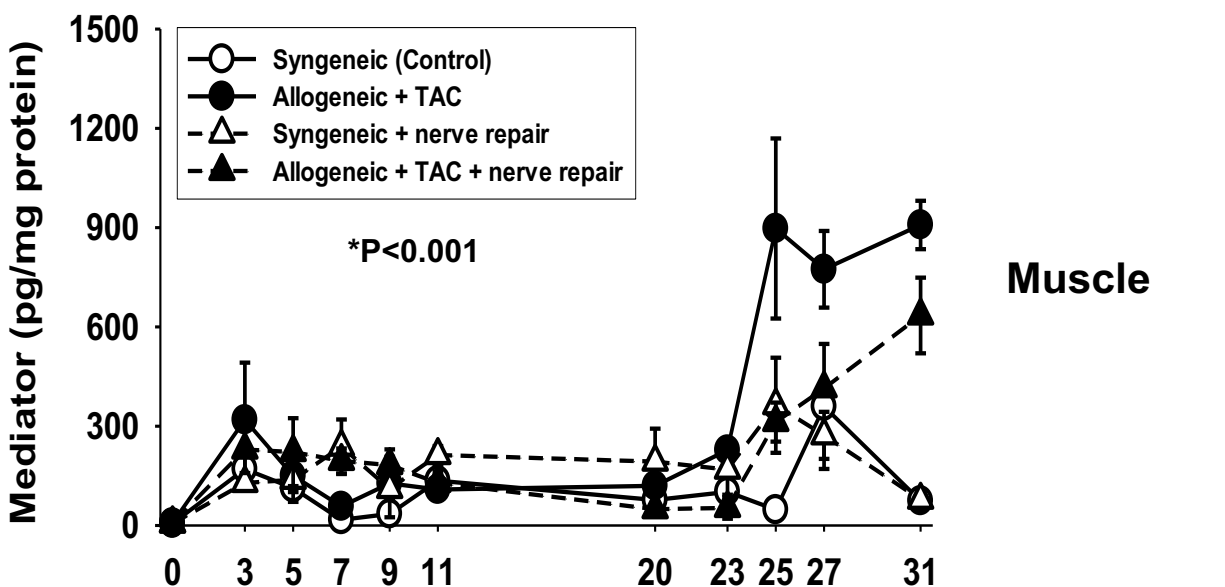

19.

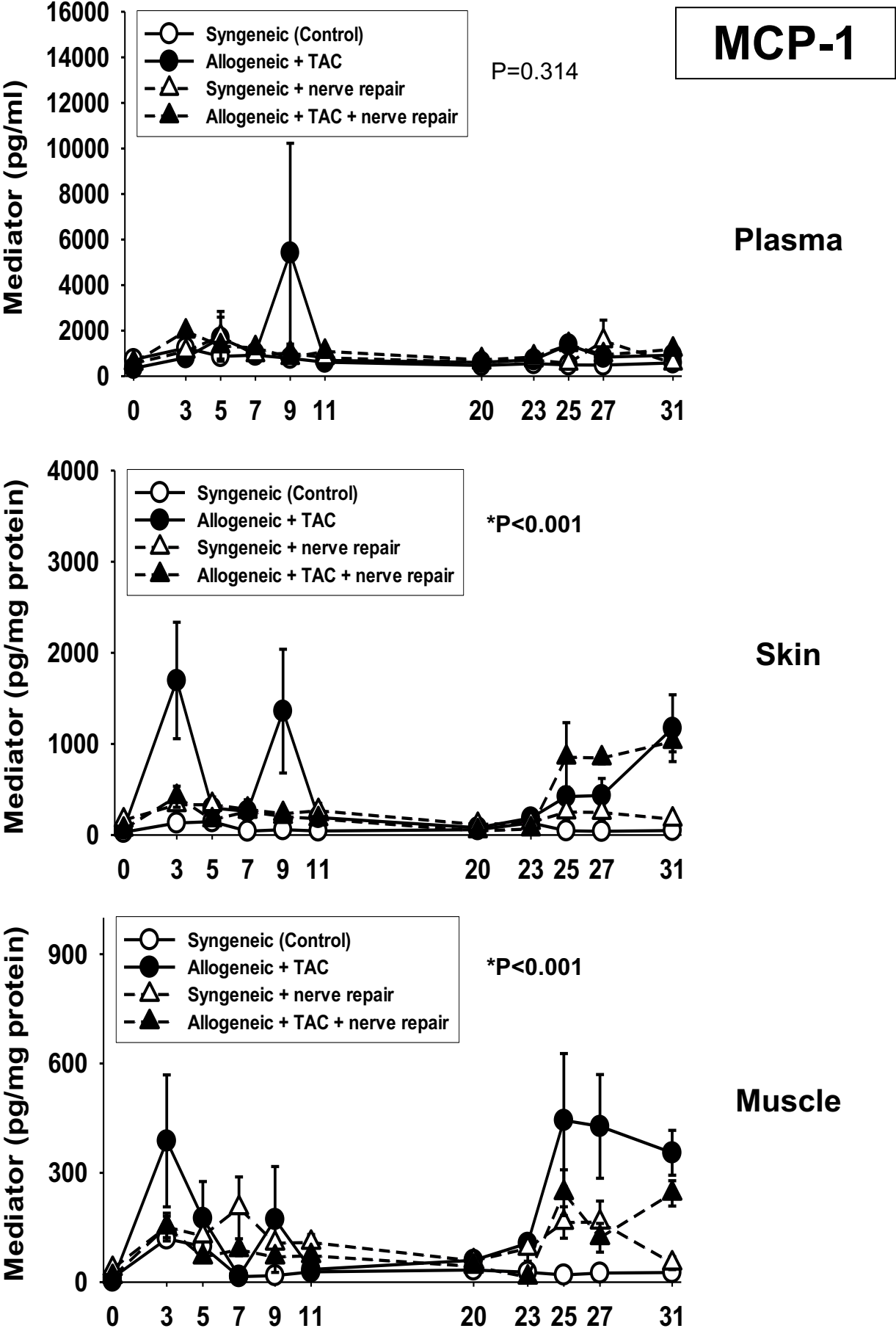

20.

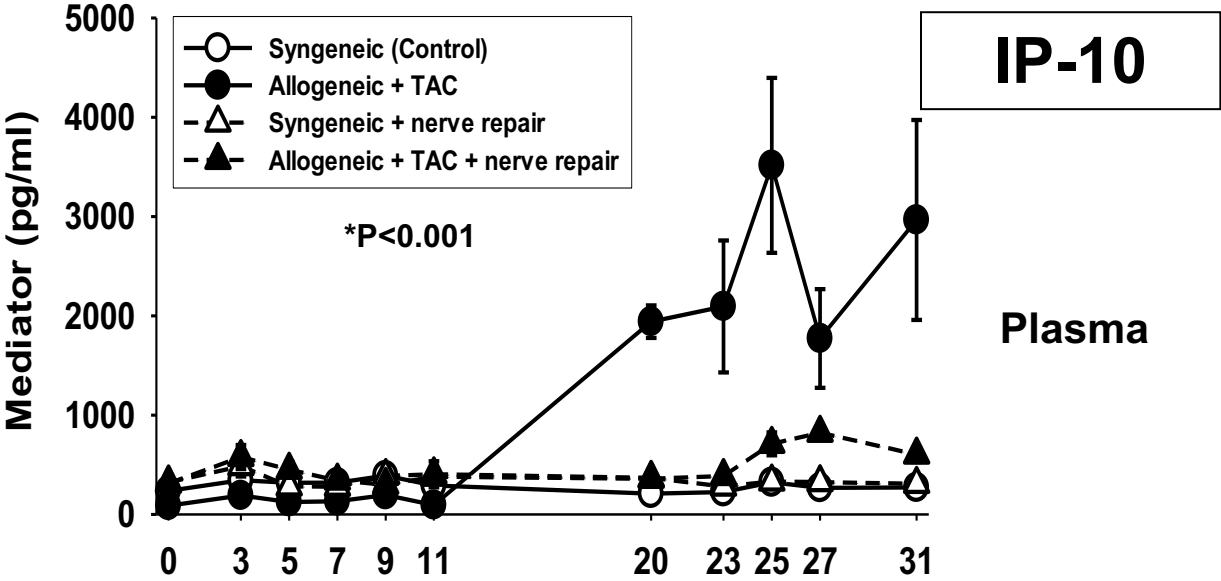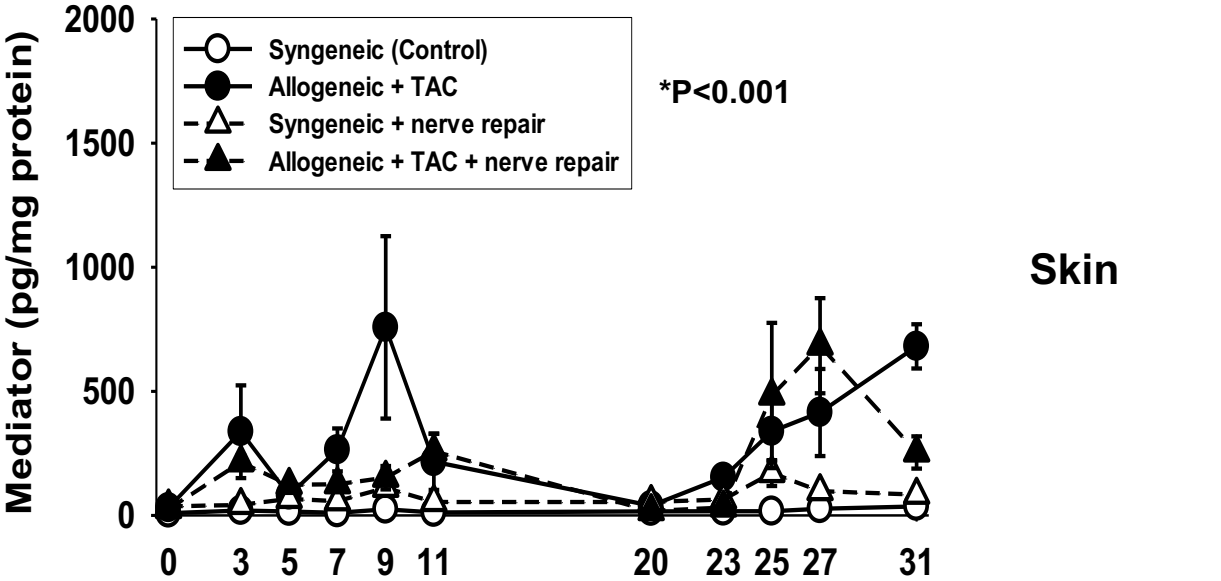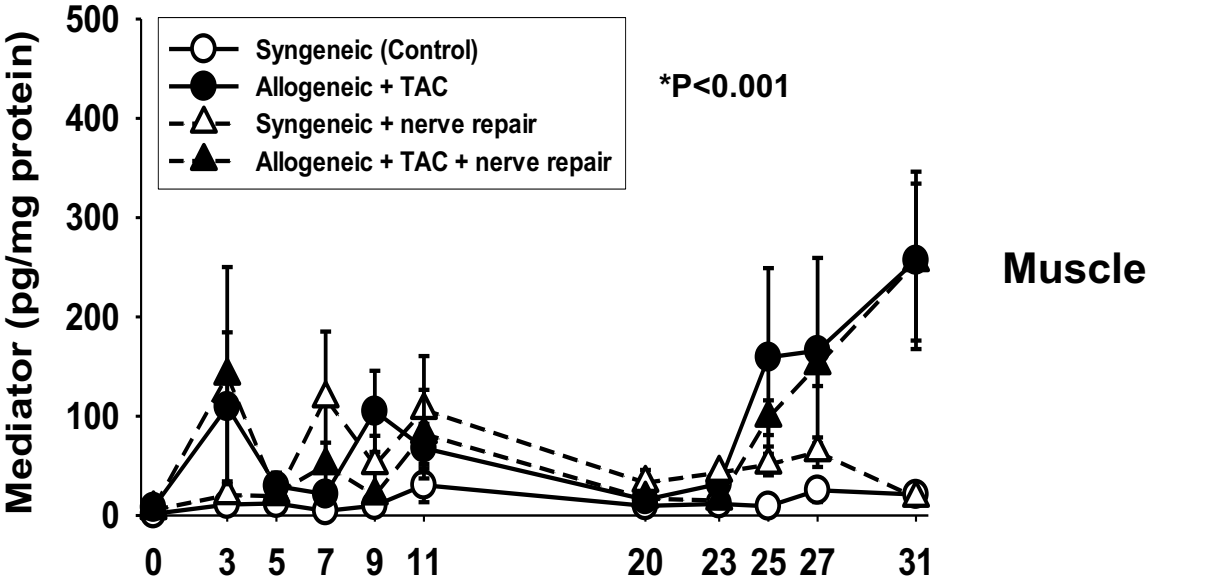

21.

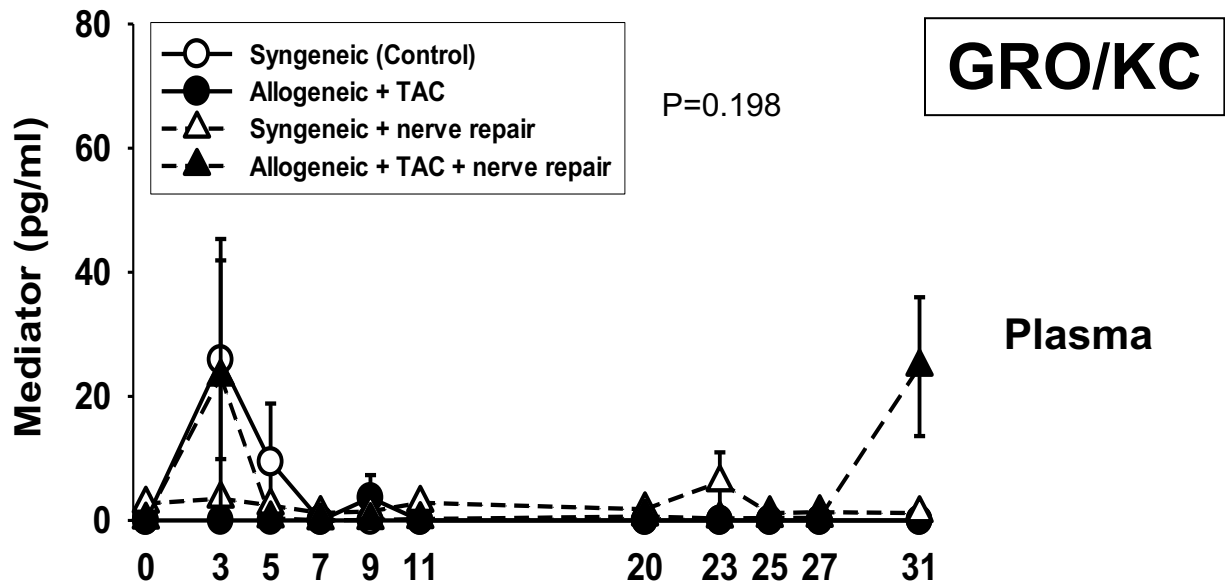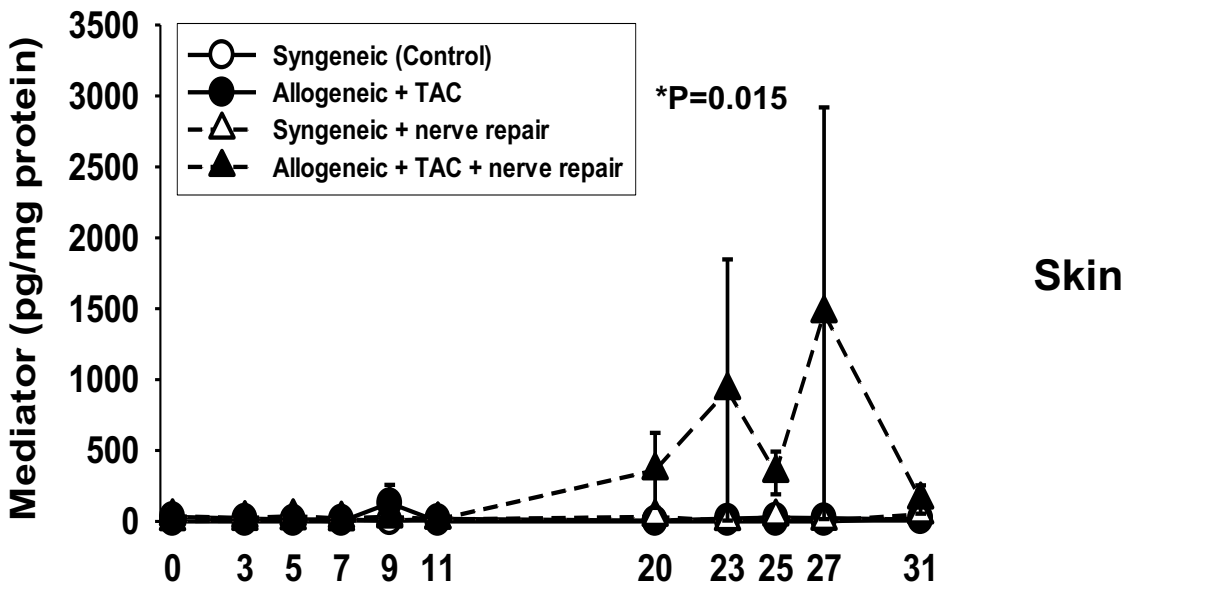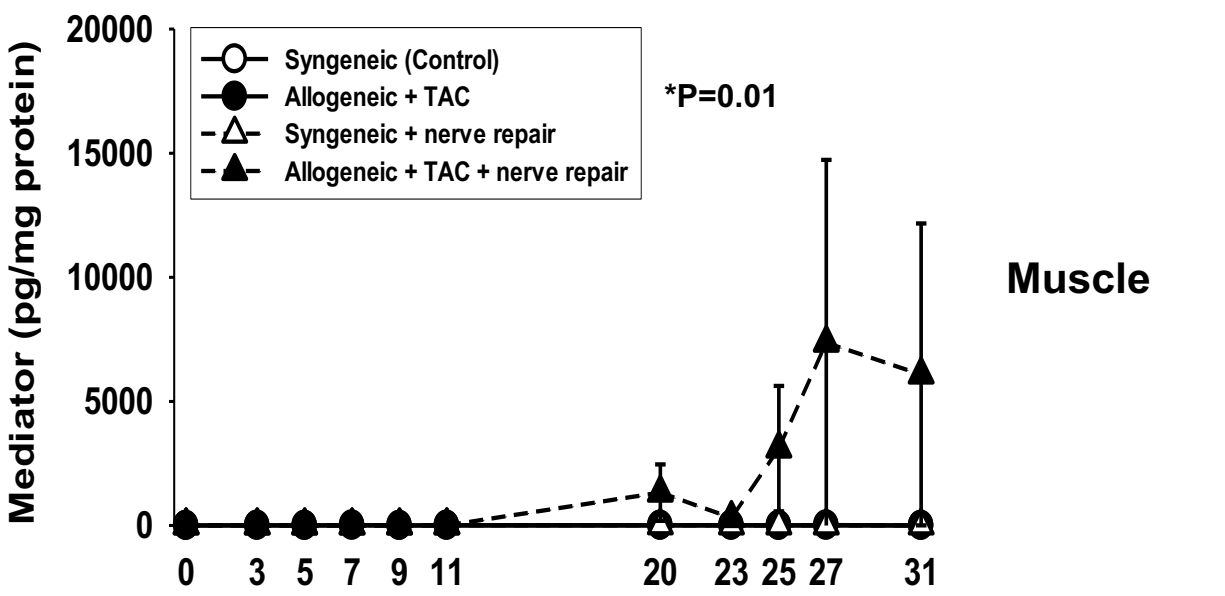

22.

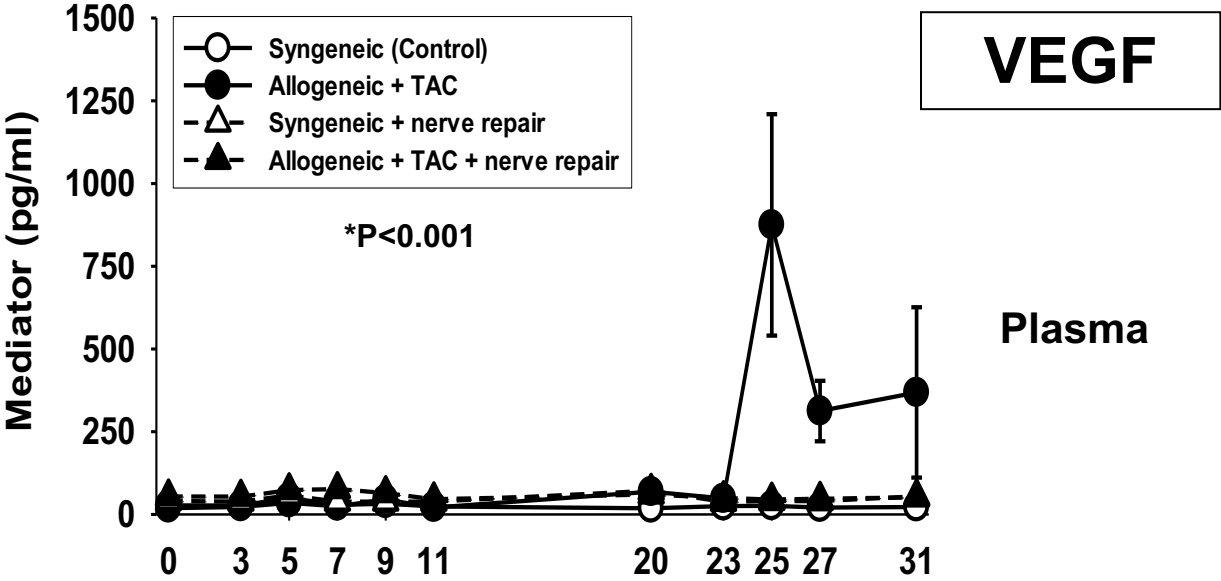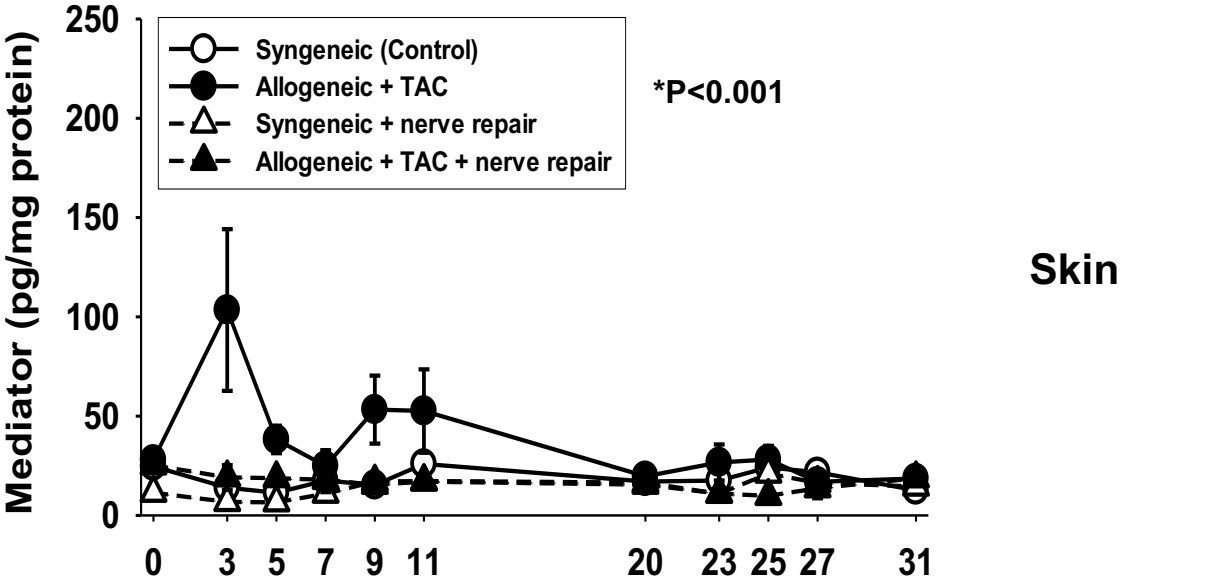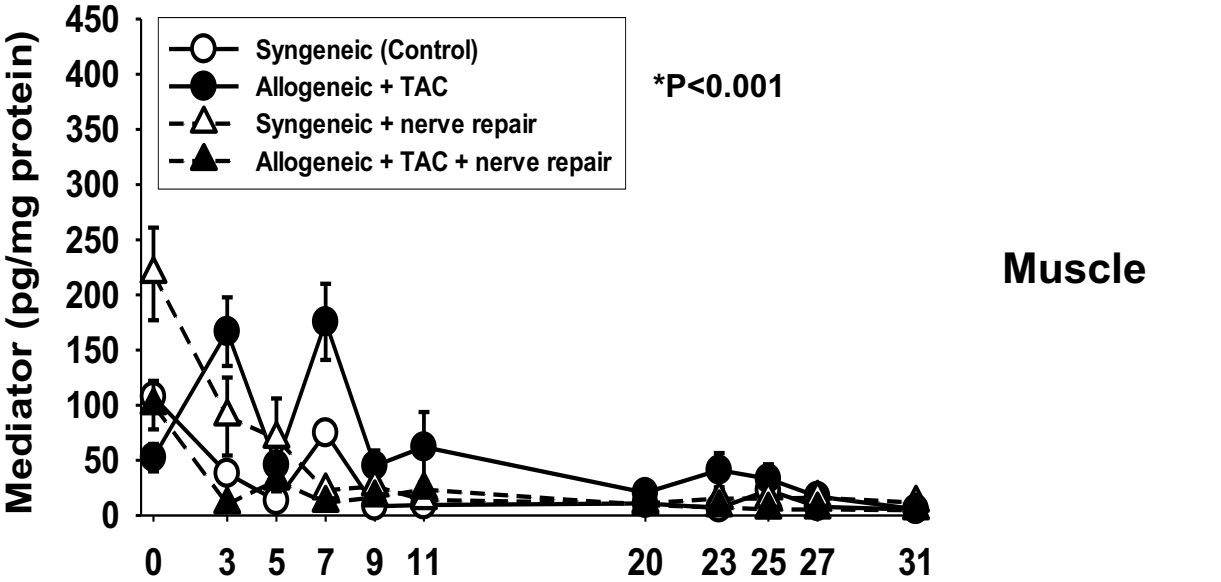

23.

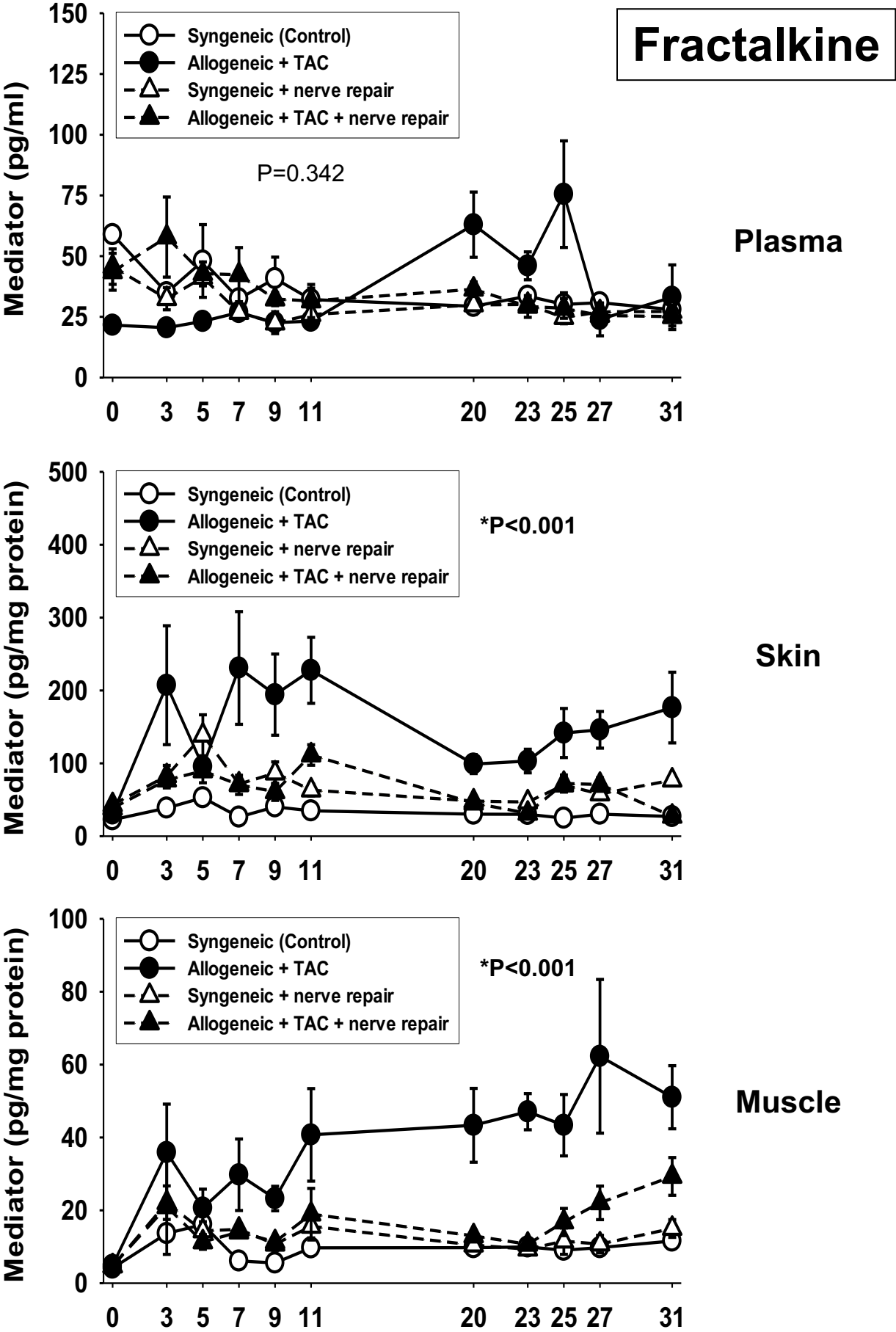

24.

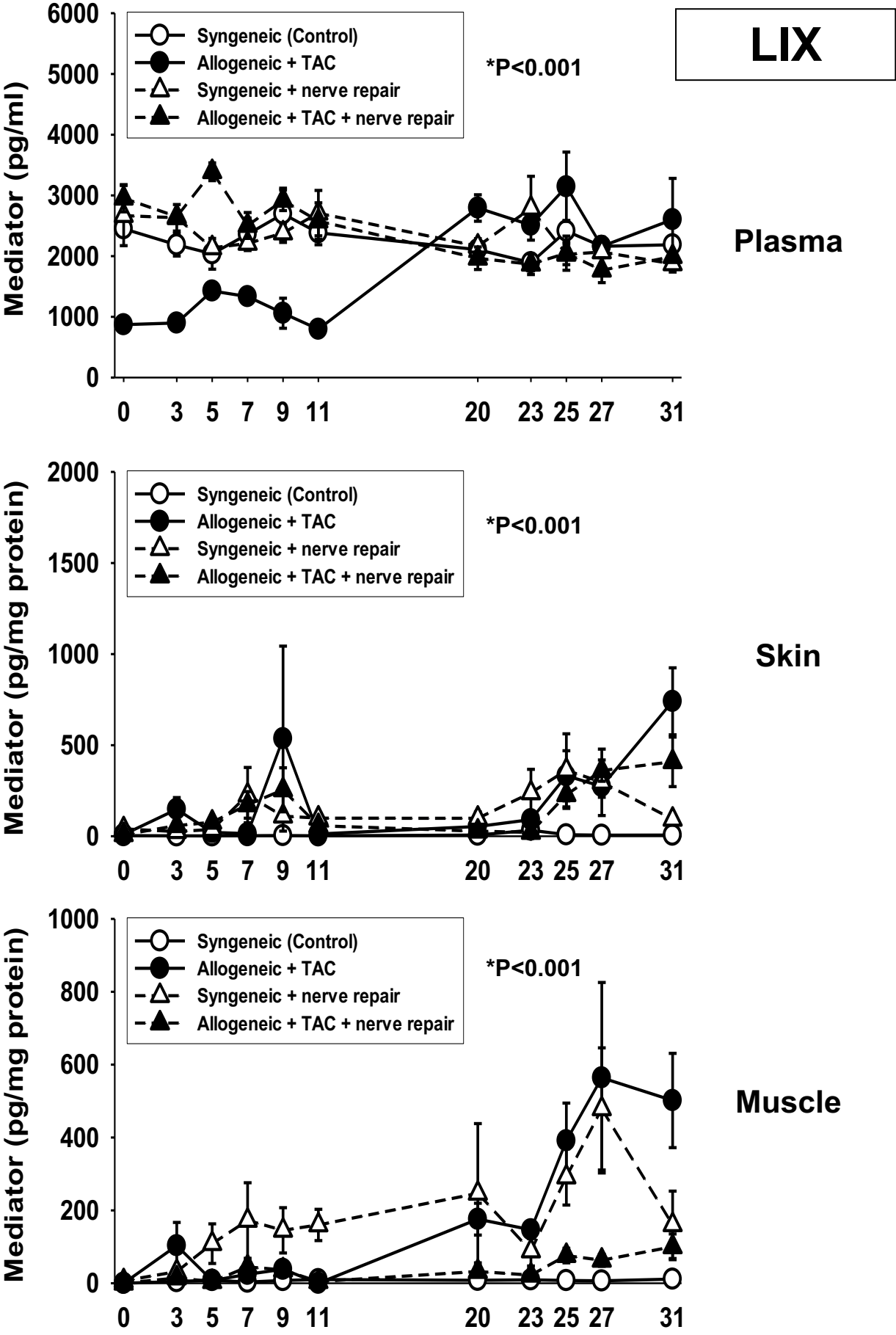

25.

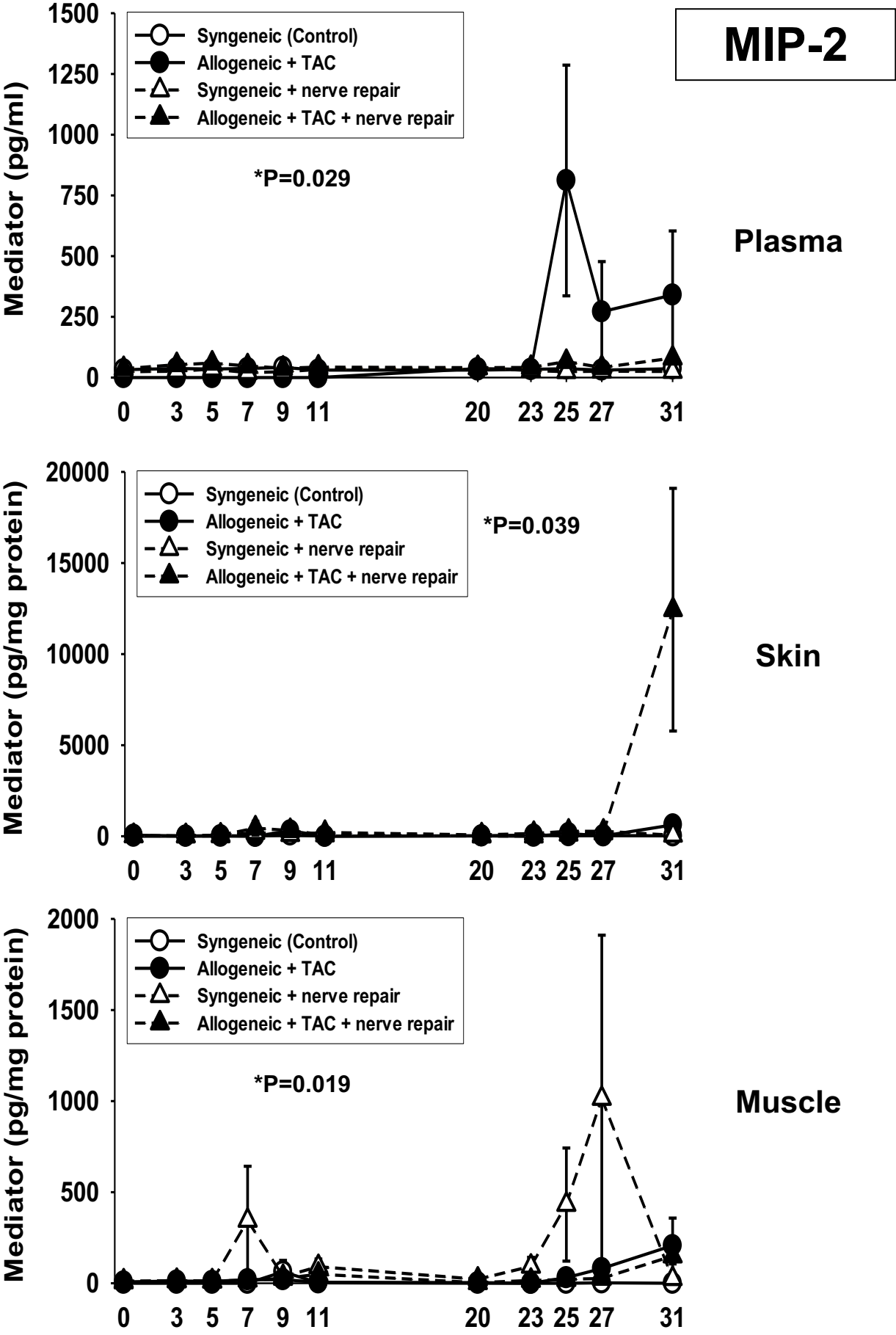

26.

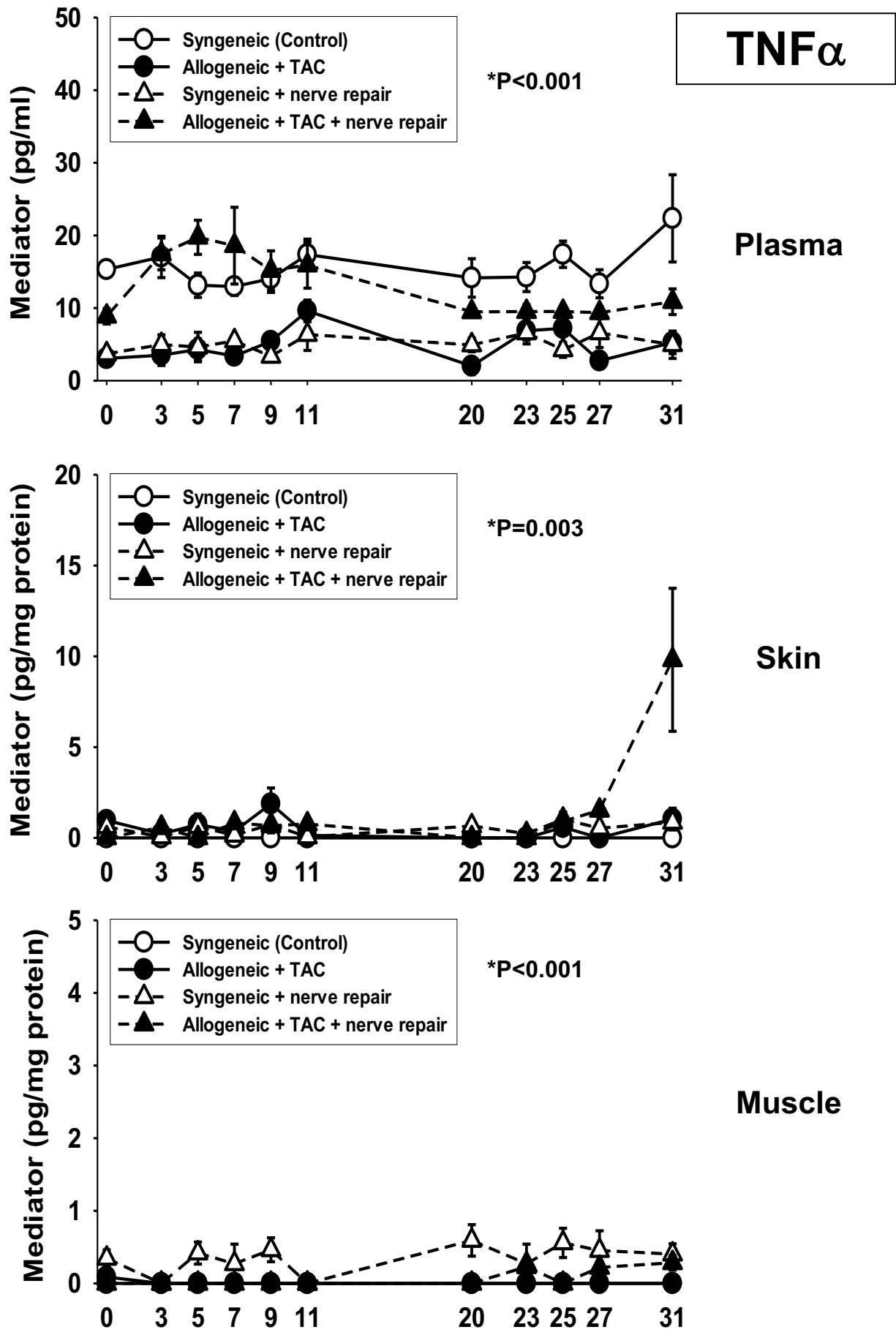

27.

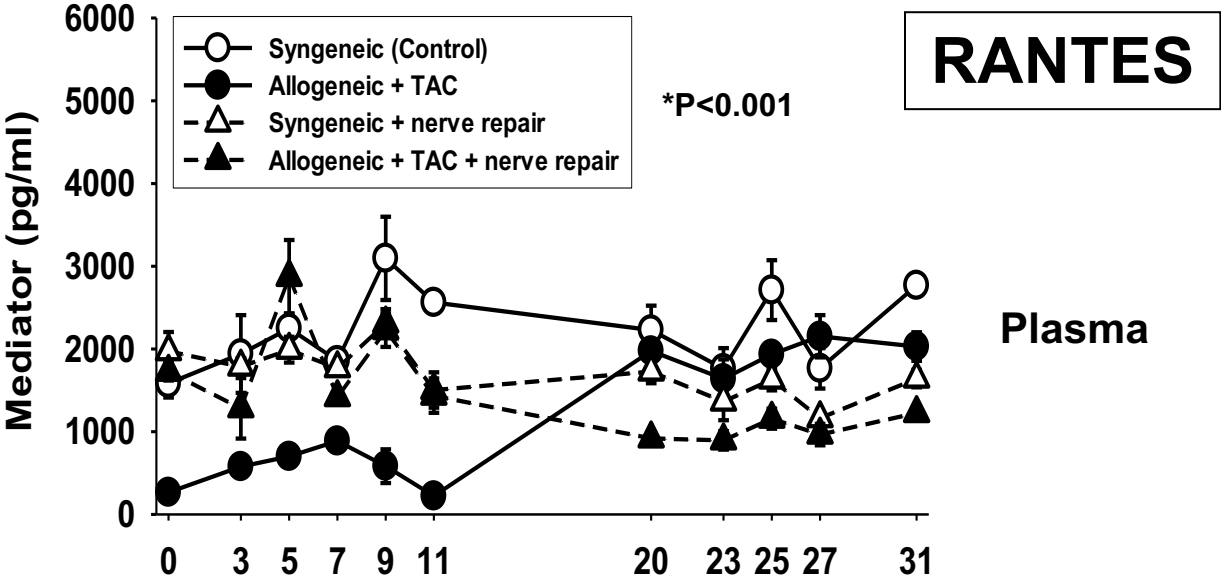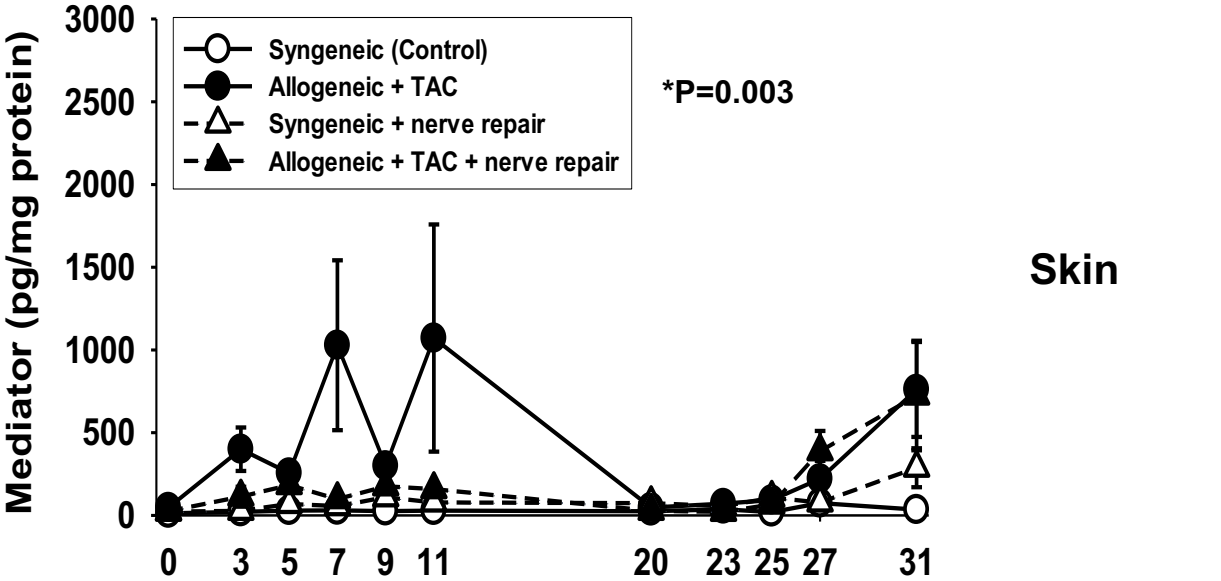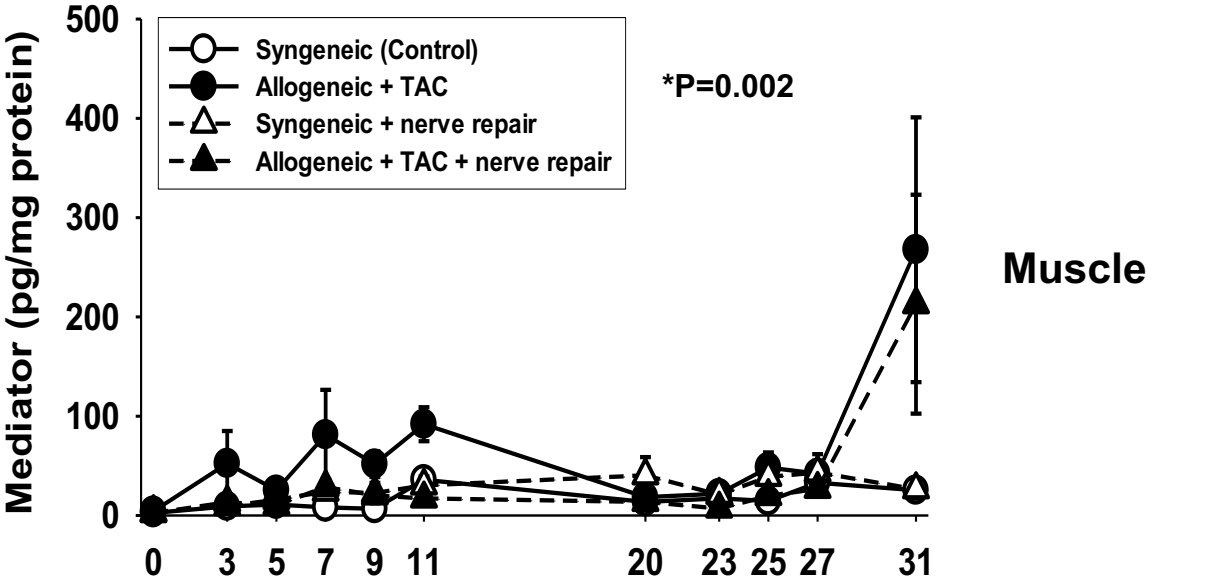

Supplement: Supplementary file 7 [file DataSheet_7.pdf]

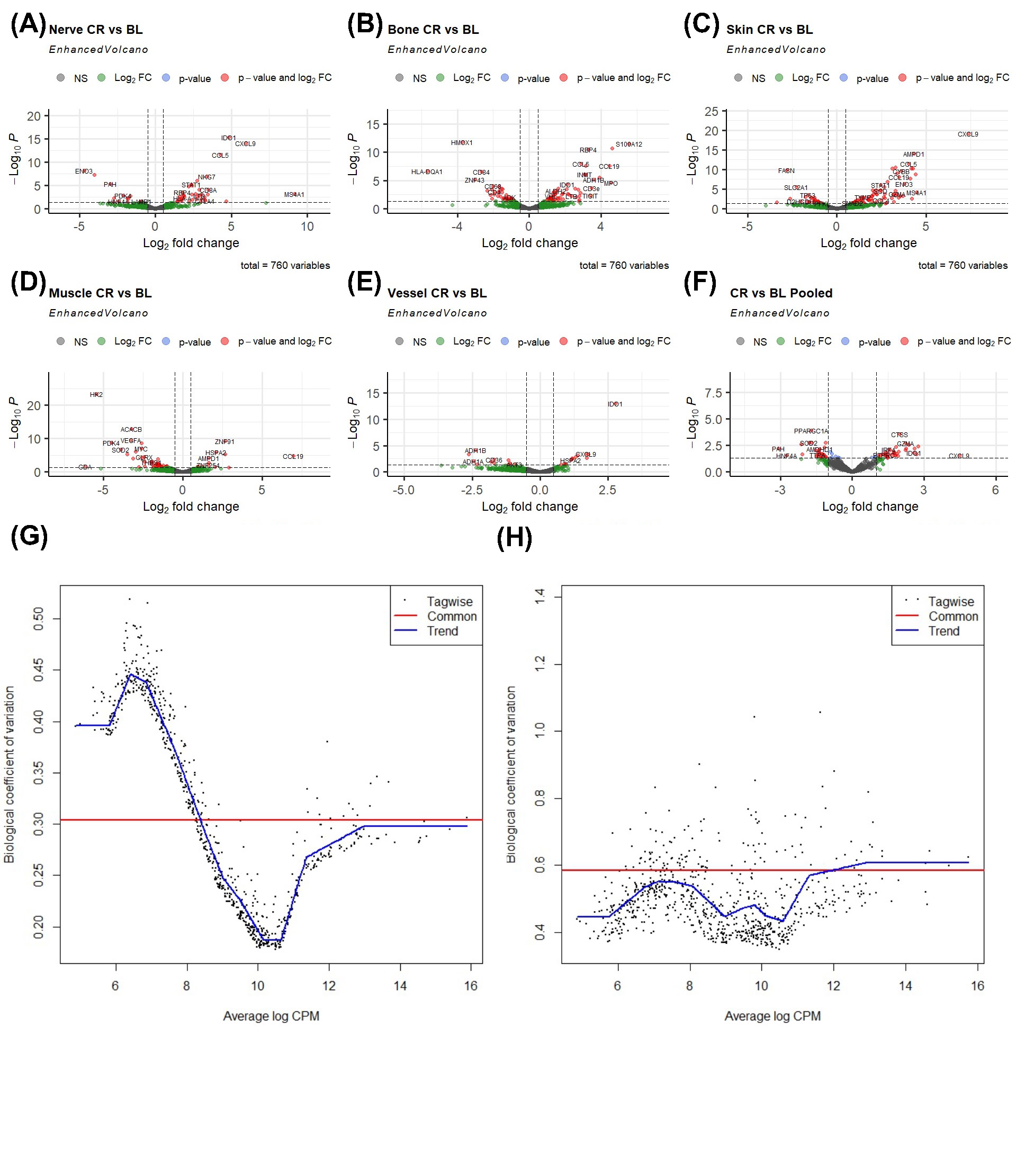

Supplement: Supplementary file 8 [file Image_1.jpeg]

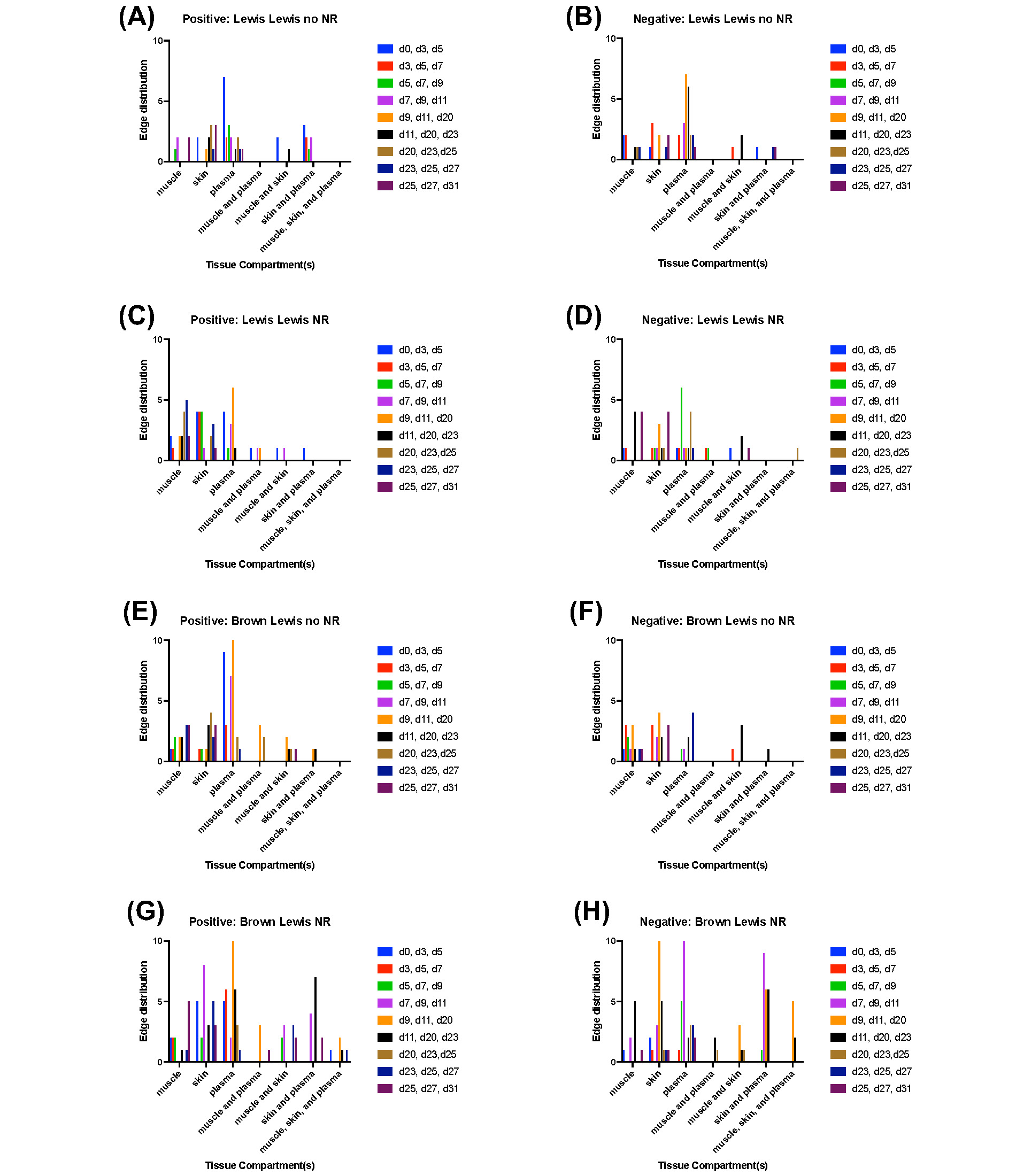

Supplement: Supplementary file 9 [file Image_2.jpeg]
