# Supplementary material for: Peripheral nerve repair is associated with augmented cross-tissue inflammation following vascularized composite allotransplantation
Source: Front Immunol. 2023 May 11;14:1151824. doi: 10.3389/fimmu.2023.1151824 (PMC10213935; doi:10.3389/fimmu.2023.1151824)

1.

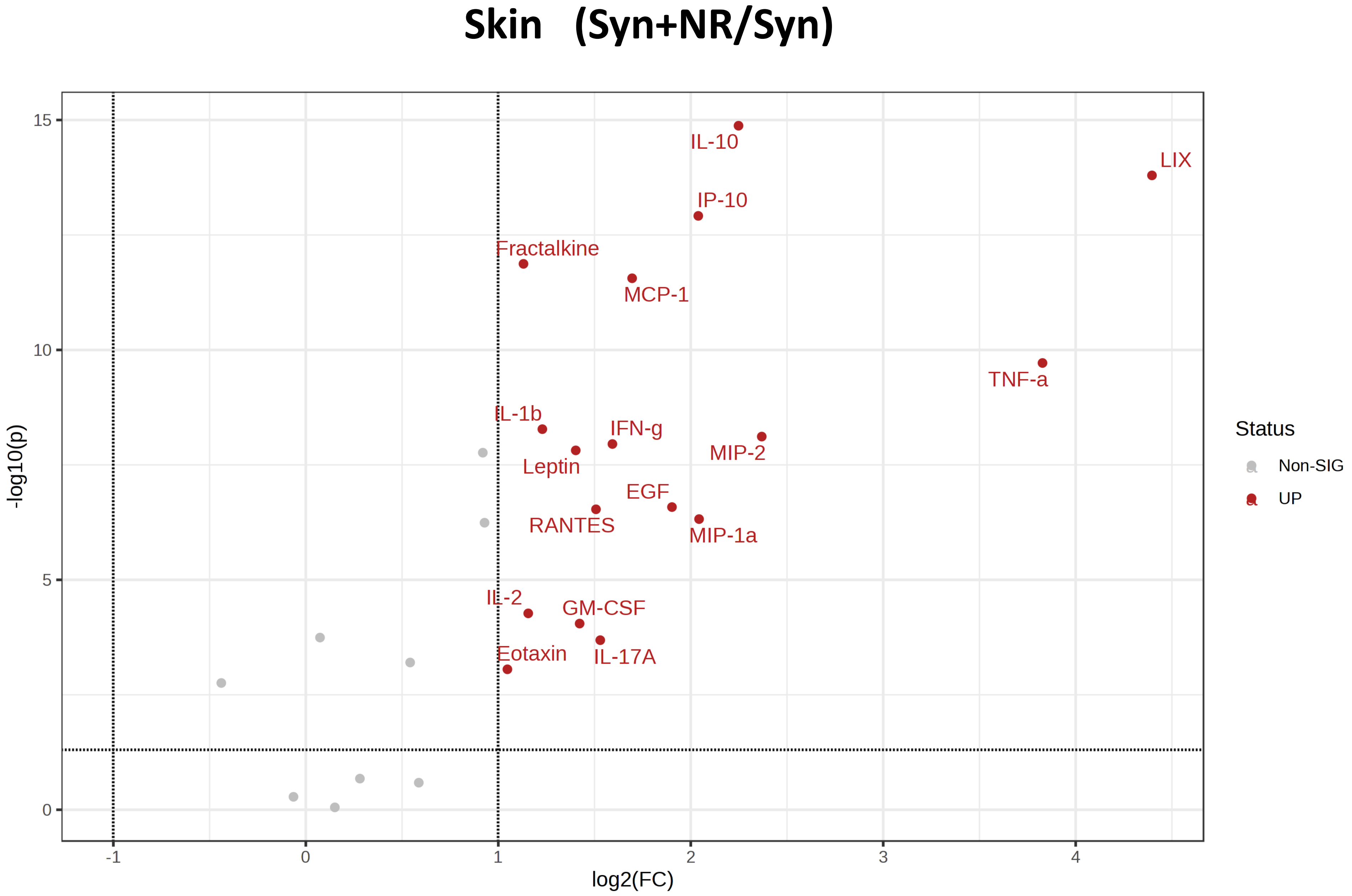
Skin (Syn+NR/Syn)
IL-10
LIX
IP-10
Fractalkine
MCP-1
TNF-a
IL-1b
IFN-g
MIP-2
Leptin
EGF
RANTES
MIP-1a
IL-2
GM-CSF
Eotaxin
IL-17A
15
10
5
0
-1
0
1
2
3
4
-log10(p)
log2(FC)
Status
Non-SIG
UP

2.

# Muscle (Syn+NR/Syn)

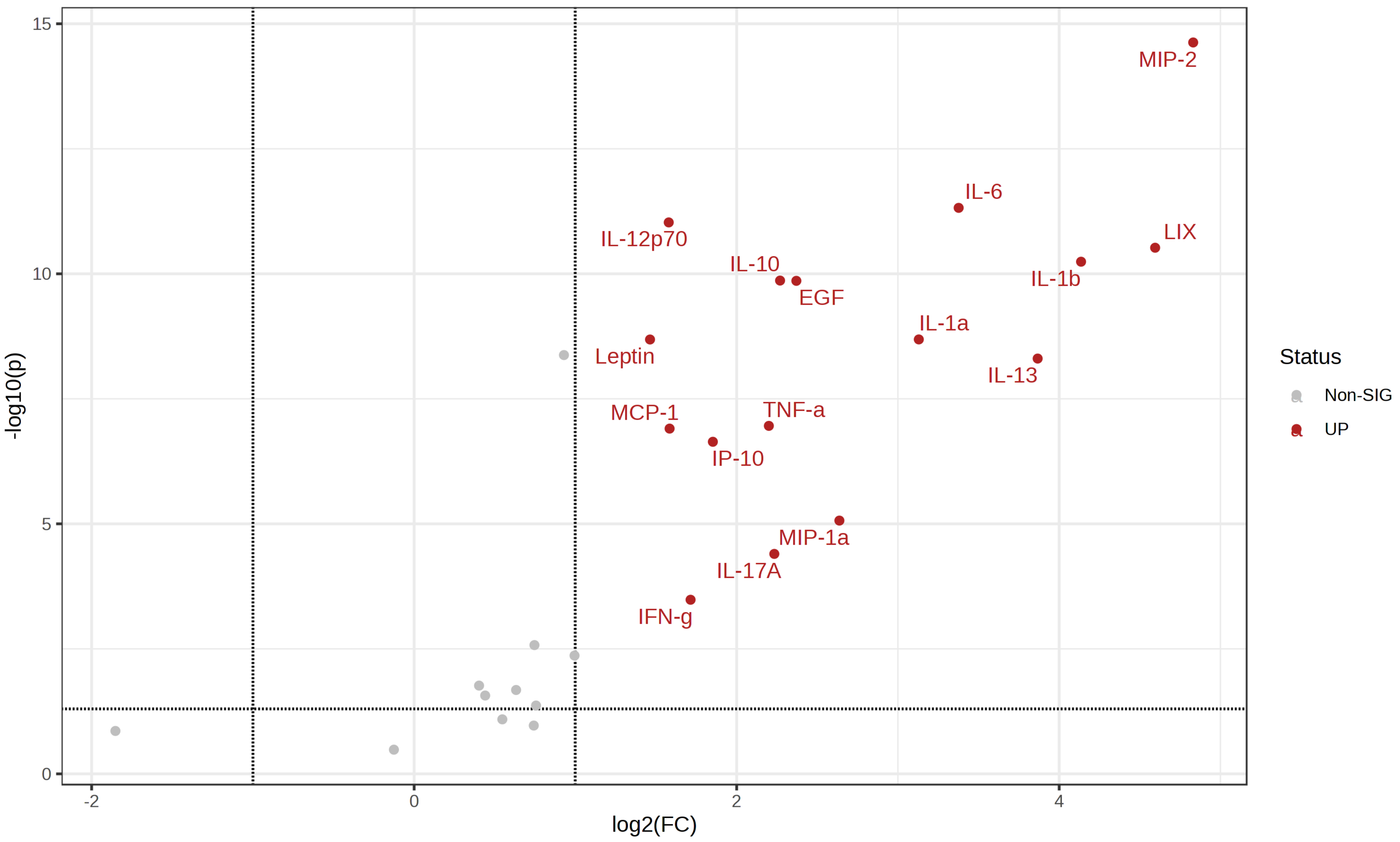

3.

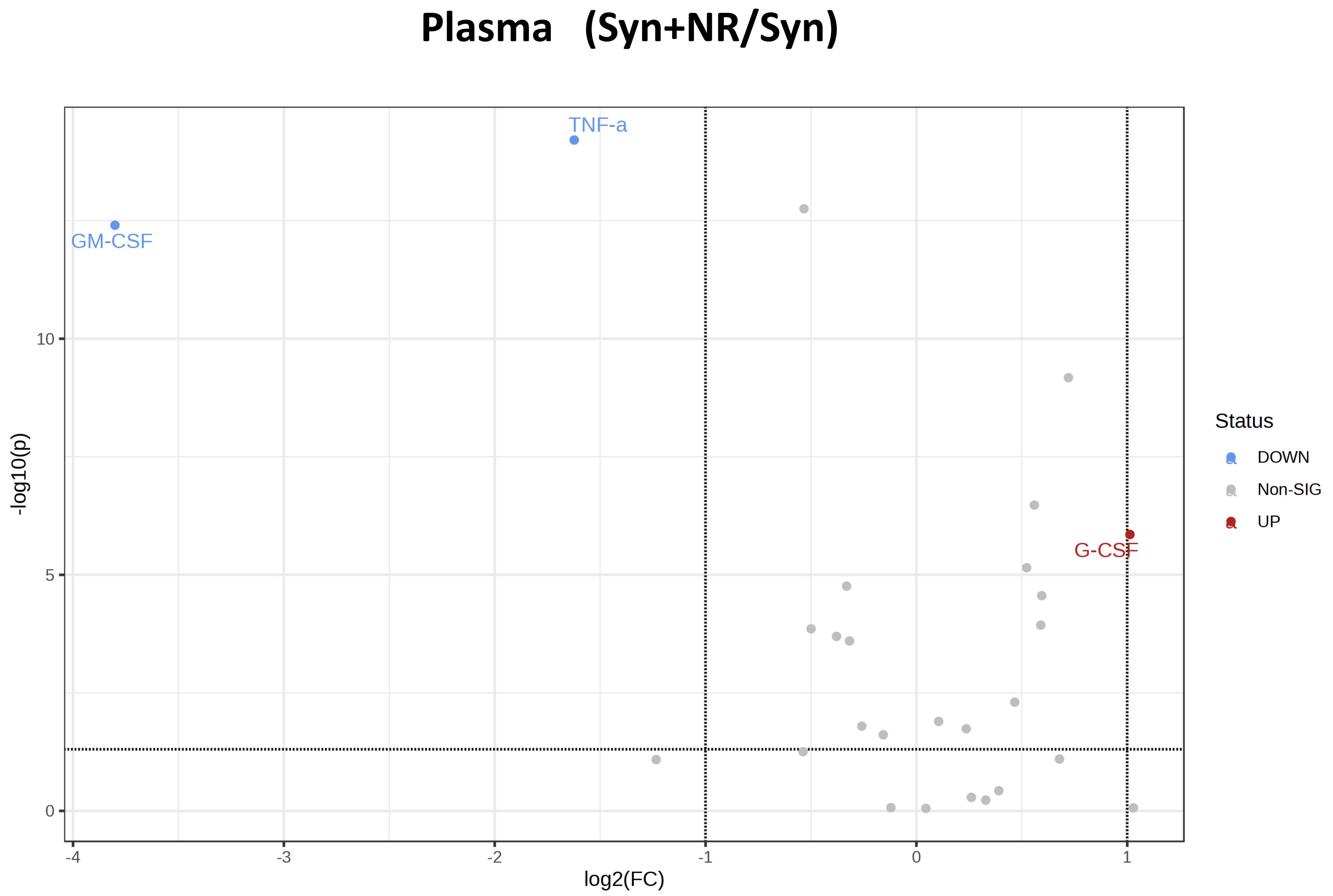

Plasma (Syn+NR/Syn)
TNF-a
GM-CSF
G-CSF
10
5
0
-log10(p)
-4
-3
-2
-1
0
1
log2(FC)
Status
DOWN
Non-SIG
UP

4.

# Skin (Allo+TAC+NR/Allo+TAC)

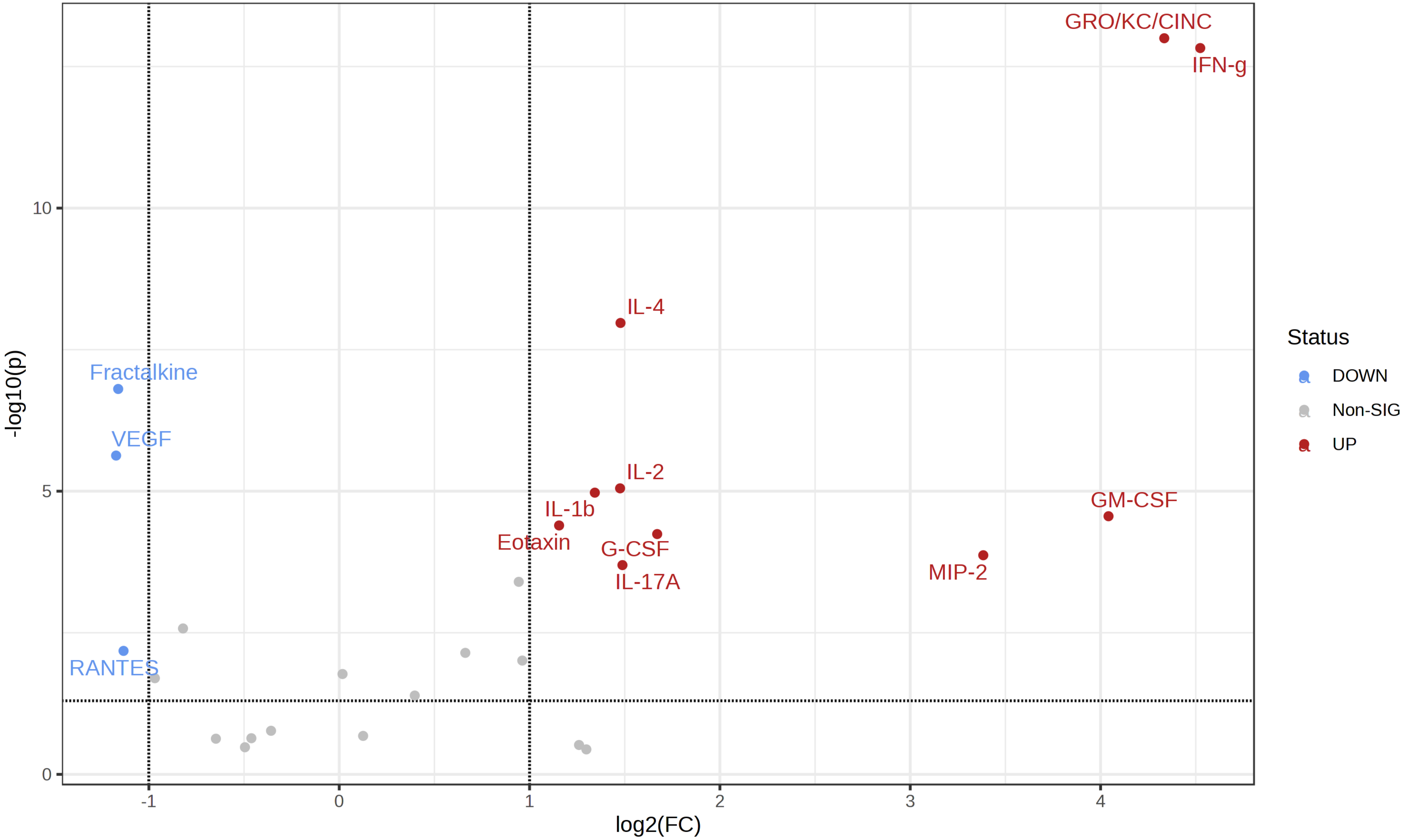

5.

# Muscle (Allo+TAC+NR/Allo+TAC)

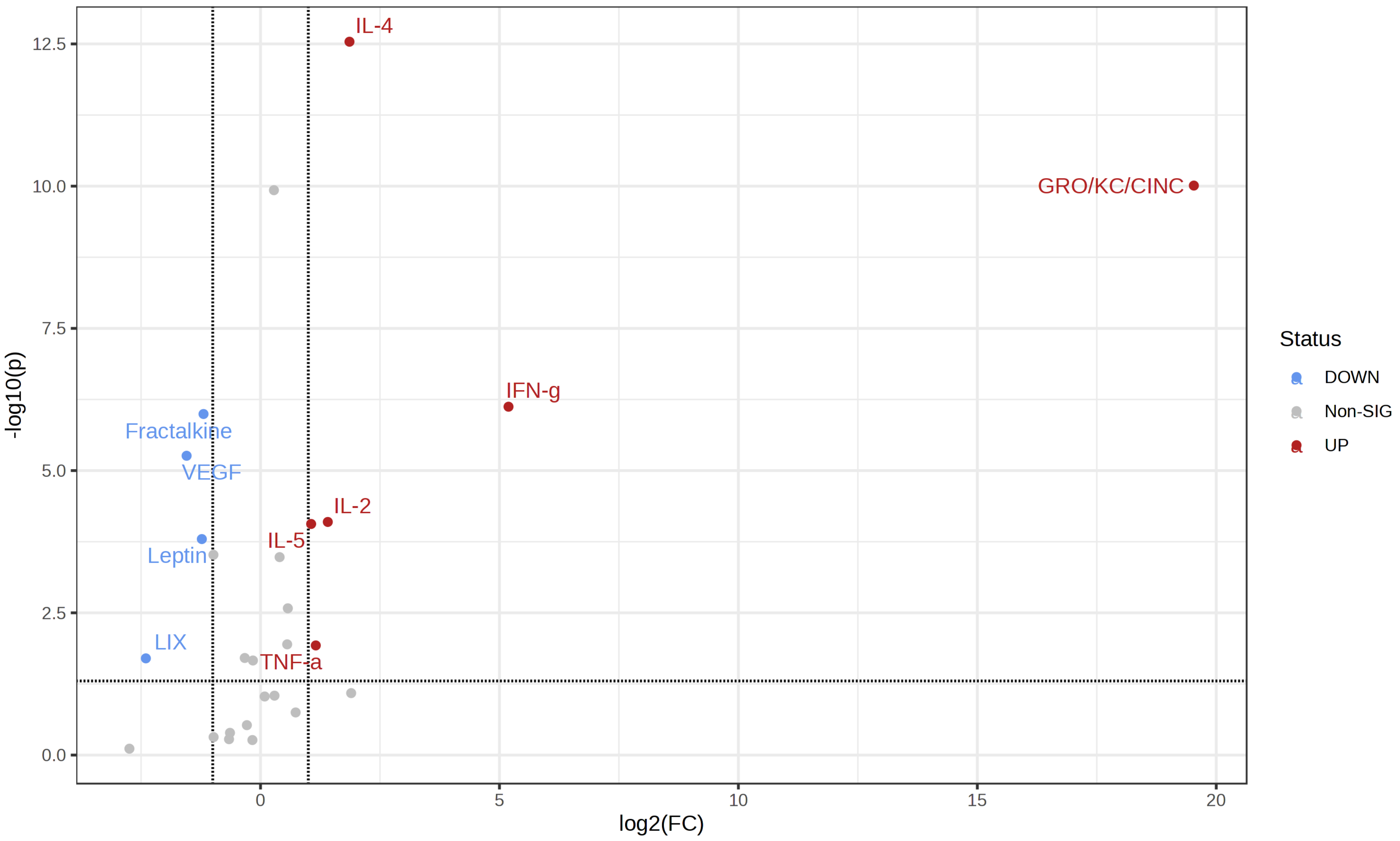

6.

# Plasma (Allo+TAC+NR/Allo+TAC)

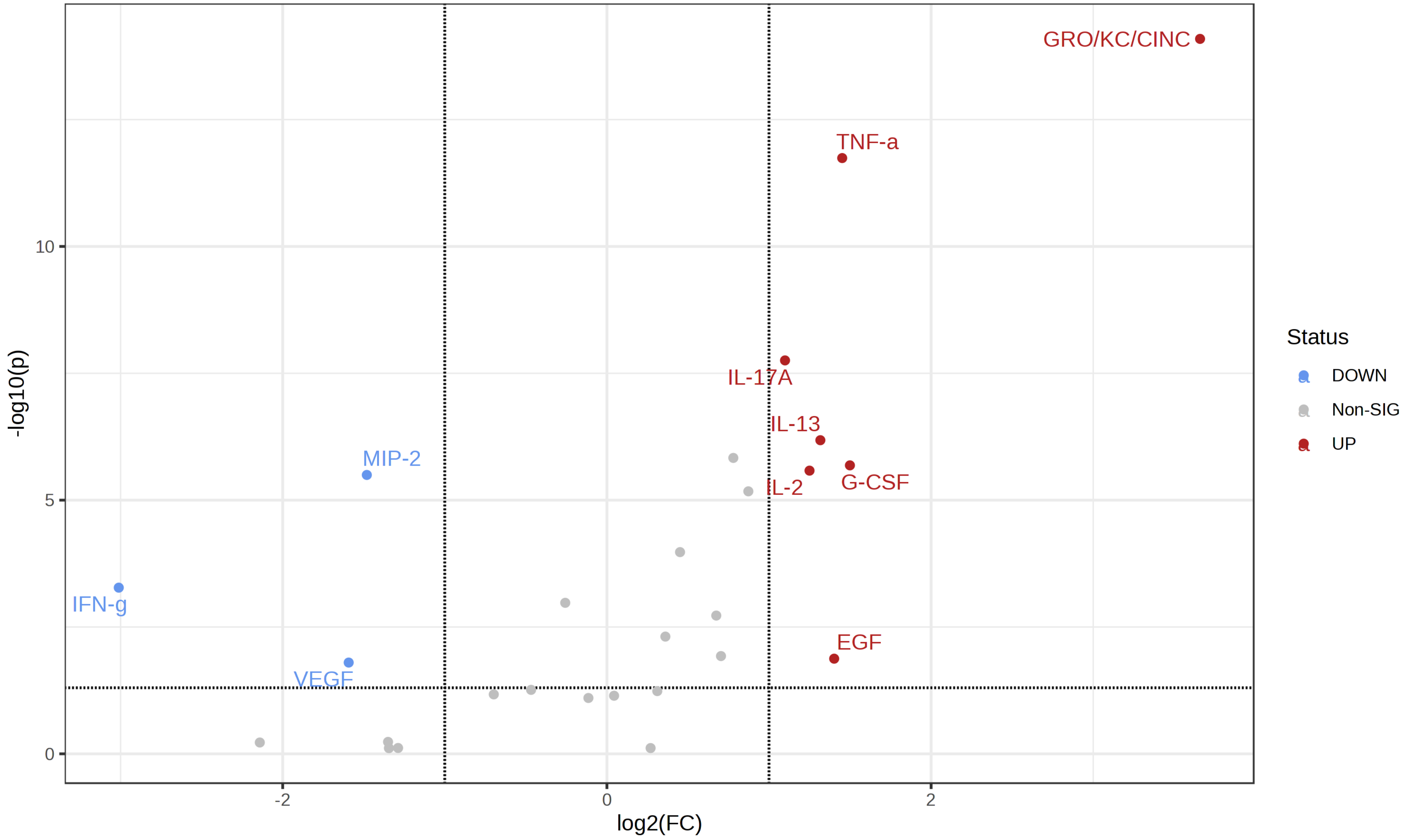

Supplement: Supplementary file 6 [file DataSheet_6.pdf]
